# Traceless Thioacid-Mediated Radical Cyclization of 1,6-Dienes

**DOI:** 10.1021/acs.joc.3c00824

**Published:** 2023-07-07

**Authors:** Dylan M. Lynch, Mark D. Nolan, Conor Williams, Leendert Van Dalsen, Susannah H. Calvert, Fabrice Dénès, Cristina Trujillo, Eoin M. Scanlan

**Affiliations:** † Trinity Biomedical Sciences Institute, Trinity College Dublin, 152-160 Pearse Street, Dublin 2, Ireland; ‡ Université de Nantes, CEISAM UMR CNRS 6230 UFR des Sciences et des Techniques, 2 rue de la Houssinière BP, 92208 − 44322 Cedex 3 Nantes, France

## Abstract

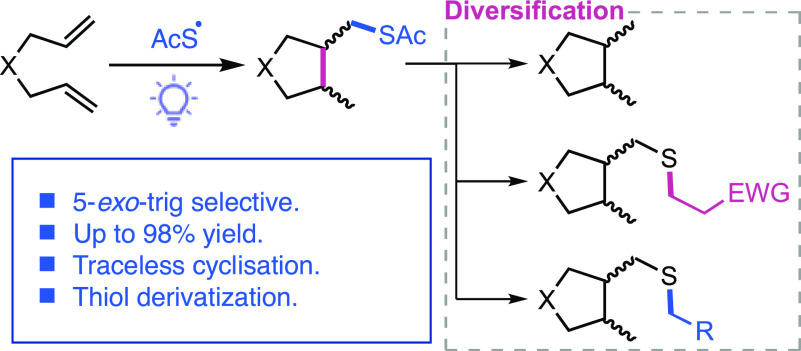

Five-membered ring systems are ubiquitous throughout natural products and synthetic therapeutics, and thus, efficient methods to access this essential scaffold are required. Herein, we report the thioacid-mediated, 5-*exo*-trig cyclization of various 1,6-dienes, with high yields of up to 98%. The labile thioester functionality can be exploited to generate a free thiol residue which can be used as a functional handle or removed entirely to provide the traceless cyclized product.

## Introduction

**Figure 1:**
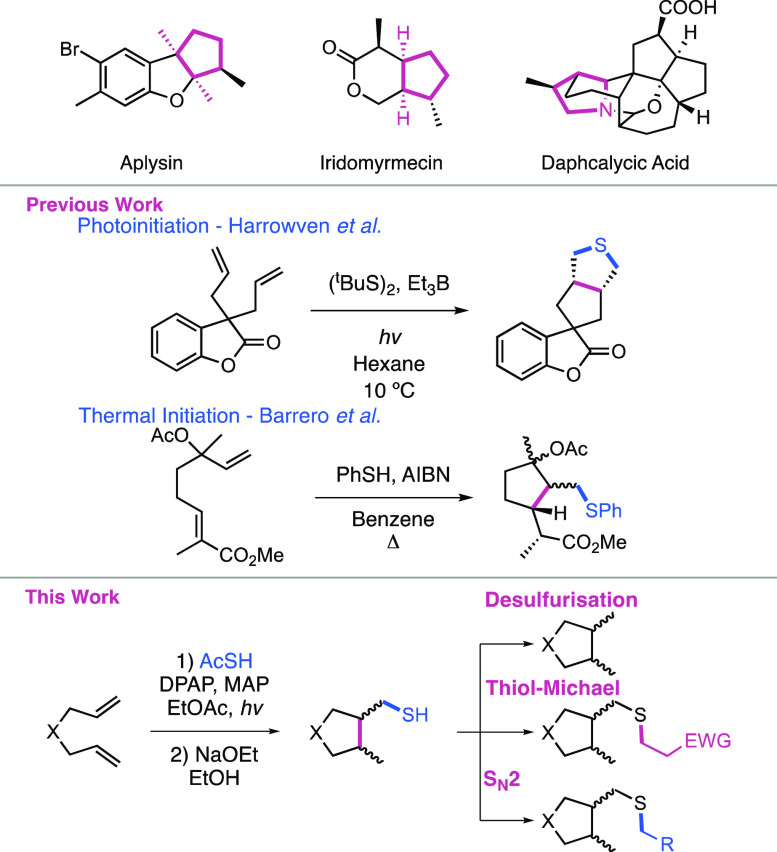
Radical-mediated 1,6-diene cyclization.

Cyclopentane, pyrrolidine, and tetrahydrofuran rings are ubiquitous scaffolds found across natural products and therapeutics and are therefore of considerable synthetic interest. In 2021, all of the “top-ten” small molecule pharmaceuticals, with cumulative sales of over $83.5 BN, contained at least one five-membered ring.^1^ Free-radical mediated cyclization reactions are widely utilized for the synthesis of functionalized ring systems with the cyclization of 1,6-di-unsaturated precursors attracting significant recent interest,^2−6^ in particular for applications in natural product synthesis.^2,7−9^ Thiyl radicals have received considerable attention as reactive intermediates with diverse applications in the fields of organic chemistry and chemical biology, as well as in polymer science.^10−15^ Cyclization of 1,6-dienes (Figure 1) through a cascade process initiated by thiyl radical addition to an alkene was first reported by Kuehne and Damon in 1977.^16^ Since then, a variety of conditions for this radical cascade reaction have been investigated. However, utilization of thioacids to furnish cyclic thioester derivatives suitable for further modification or concomitant desulfurization has not previously been reported. Moreover, detailed discussion on the scope and establishment of limitations remains elusive. Herein, we report a rapid and mild, 1,6-diene cyclization reaction propagated by sulfur-centered radicals, employing AcSH as the radical source. Furthermore, we investigate the impact of varying alkene substitution on the cyclization to provide insight into the limitations of such reaction-types, as well as computational insight into the origin of *cis*-selectivity. Finally, we exploit the thioacetate group present in the products of this reaction for further functionalization, including desulfurization, Michael addition followed by oxidation to sulfones, and S_N_2 chemistry.

## Results and Discussion

Our initial investigation focused on diallylmalonate **1** in the presence of thioacetic acid (AcSH) under UV-initiated conditions (Table 1). As expected, in the absence of UV irradiation and under an inert atmosphere, no cyclization was observed (entries 1 and 2). Irradiation in EtOAc in the presence of 2,2-dimethoxy-2-phenylacetophenone (DPAP) and 4-methoxyacetophenone (MAP) gave quantitative conversion to the cyclized product (entry 3). Use of either DPAP only or MAP only gave slightly reduced yields. Irradiation at 365 nm in the absence of DPAP and MAP furnished the cyclized product, albeit in reduced yield with a longer reaction time of 3 h (entry 6). Application of O_2_-initiated conditions previously utilized within the group^17,18^ gave reasonable conversion (entry 5). Blue LED photoactivation conditions were investigated with a range of initiators (entries 8–10) with Eosin Y and 9*H*-thioxanthen-9-one emerging as optimal initiators under these conditions (entry 8 and 10). Addition of TEMPO prevented any product formation, confirming the radical nature of the process. NMR timescale experiments over 2 h showed that this was sufficient time for consumption of the starting material while the control reaction in dark conditions showed no appreciable consumption of the alkene over 2 h.

**Table 1:**
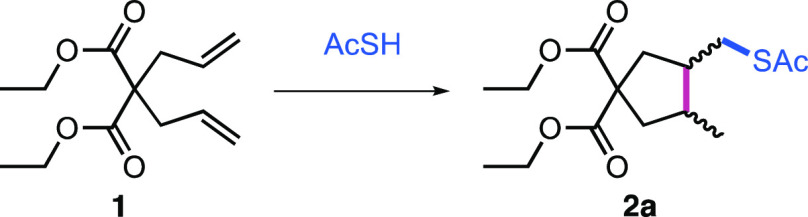
Optimization of Reaction Conditions^a^

entry	additive	solvent	λ (nm)	conversion (%)
1	DPAP/MAP	CDCl_3_		0
2^b^		CDCl_3_		0
3	DPAP/MAP	EtOAc	365	99
4	DPAP	CDCl_3_	365	96
5	MAP	CDCl_3_	365	94
6^c^		CDCl_3_	365	70
7	atm. O_2_	EtOAc		68
8	eosin Y	MeCN	420	88
9	acridine orange	CH_2_Cl_2_	420	41
10	9*H*-thioxanthen-9-one	CH_2_Cl_2_	420	86

a Standard conditions: 0.1 M **1**, DPAP (10 mol %), MAP (10 mol %), 2 h. Conversion measured by ^1^HNMR by integration of the product peak compared to the internal standard.

b Inert atmosphere.

c 3 h reaction time.

**Scheme 1:**
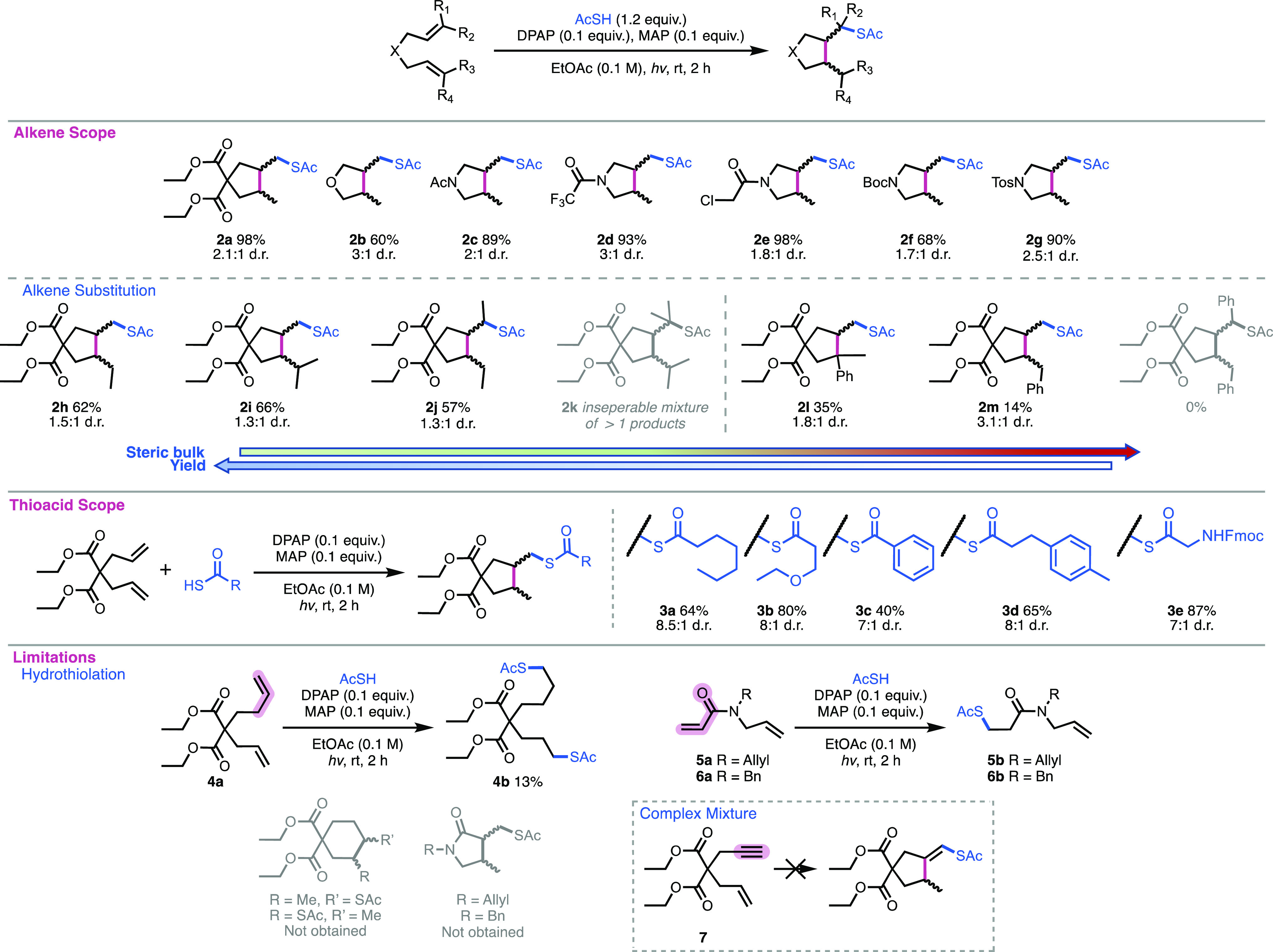
Scope and Limitations for Thioacid-Initiated 1,6-Diene Cyclizations

Following optimization of the cyclization of the model substrate, we sought to evaluate the scope of the reaction (Scheme 1) in relation to heteroatomic substrates bearing N, O, or S atoms within the 1,6-diene backbone, in part due to their high frequency in therapeutics and natural products. Initial attempts to cyclize diallylamine were unsuccessful, although this is not surprising given the basic nature of the amine.^19^ Diallyl ether was successfully cyclized to give **2b** with 60% yield; however, for diallyl sulfide, only traces of the desired product were detected, presumably due to the fragmentation of the beta-thioalkyl radical intermediate. Contrary to simple diallylamine, *N*-protected diallylamine-based substrates delivered the desired *N*-protected pyrrolidines in high yields of up to 98%. For instance, diallylacetamide cyclization yielded **2c** in 89% yield. Likewise, trifluroacetylated and chloroacetylated derivatives gave excellent respective yields of 93% (**2d**) and 98% (**2e**). Boc and tosyl protection was also well tolerated, pyrrolidines **2f** and **2g** being obtained in 68 and 90% yields, respectively.

We then turned to investigation of varying the degree of substitution on the alkene groups of the 1,6-diene scaffold. A range of di- and tri-substituted alkene-bearing substrates was synthesized. These precursors bearing either methyl or phenyl substituents were subjected to optimized cyclization conditions. High levels of regioselectivity were observed when one of the two C=C bonds was nonterminal, with the addition of the acylthiyl radical taking place exclusively at the least sterically hindered alkenes (**2h** and **2i**). Interestingly, precursor **1j** presenting two internal, di-substituted alkenes furnished the desired cyclic compounds in good yield (57% for **2j**). However, **1k** having two trisubstituted alkenyl moieties gave an inseparable mixture of products containing cyclic and singly hydrothiolated species. Substitutions with phenyl groups at the alkene led to significant decreases in yields (**2l** and **2m**), to the point of no reaction of the substrate bearing a phenyl substituent on both alkenes, most likely due to the formation of resonance-stabilized intermediates which inhibit the radical chain process, as well as due to the poorer reactivity of styrene-type alkenes to thiyl radicals.^20^

Brief investigation into variation of the thioacid component of the reaction demonstrated that this transformation is not unique to AcSH and can be used to generate a diverse range of thioesters. In the case of synthetically prepared thioacids, the corresponding *S*-trityl thioester was deprotected using 25% TFA in DCM in the presence of ethyldimethylsilane and dried in vacuo directly prior to use in the cyclization reaction without further purification. The aliphatic thioheptanoic acid gave reasonable yield of **3a** at 64%, with a good d.r. of 8.5:1. The other aliphatic thioacid example **3b** gave a very good yield of 80%, also with good d.r. of 8:1. Aromatic thioacids yielded **3c** and **3d** in moderate yields and good d.r. The glycine amino acid derivative yielded **3e** in very good yield and good d.r. Importantly, these results demonstrate the tolerance of more sterically hindered thioacids than AcSH, often with improved d.r. This can facilitate the use of different thioacids to improve d.r. but at the potential expense of yield.

We also investigated the potential of this methodology for generation of larger ring systems. 1,7-Diene **4a** was synthesized to potentially afford the larger, 6-membered ring. Subjecting this substrate to the cyclization conditions, however, gave no cyclized product, instead furnishing bis-hydrothiolated product **4b** in 13% yield. This is likely due to must faster kinetics for attack of the thiyl radical on the alkene when compared to cyclization of the larger 6-membered system, noting that thiyl radical addition to alkenes is often a highly efficient process. Furthermore, the 6-membered transition state would require different structural conformation. The cyclization of substrates **5a** and **5b** containing a single α,β-unsaturated moiety was also investigated; however, neither yielded cyclic products. We then turned to investigation of cyclization of enyne **7**. A small amount of alkene consumption was observed for this substrate when equimolar quantities of AcSH were used, but no cyclization product was obtained. Use of excess AcSH (3 equiv) gave complete consumption of both alkene and alkyne, but again, no cyclic products could be isolated. Despite the limitations of this methodology toward formation of larger ring sizes, these results show potentially beneficial selectivity for 1,6-diene systems over other unsaturated pi-systems.

**Figure 2:**
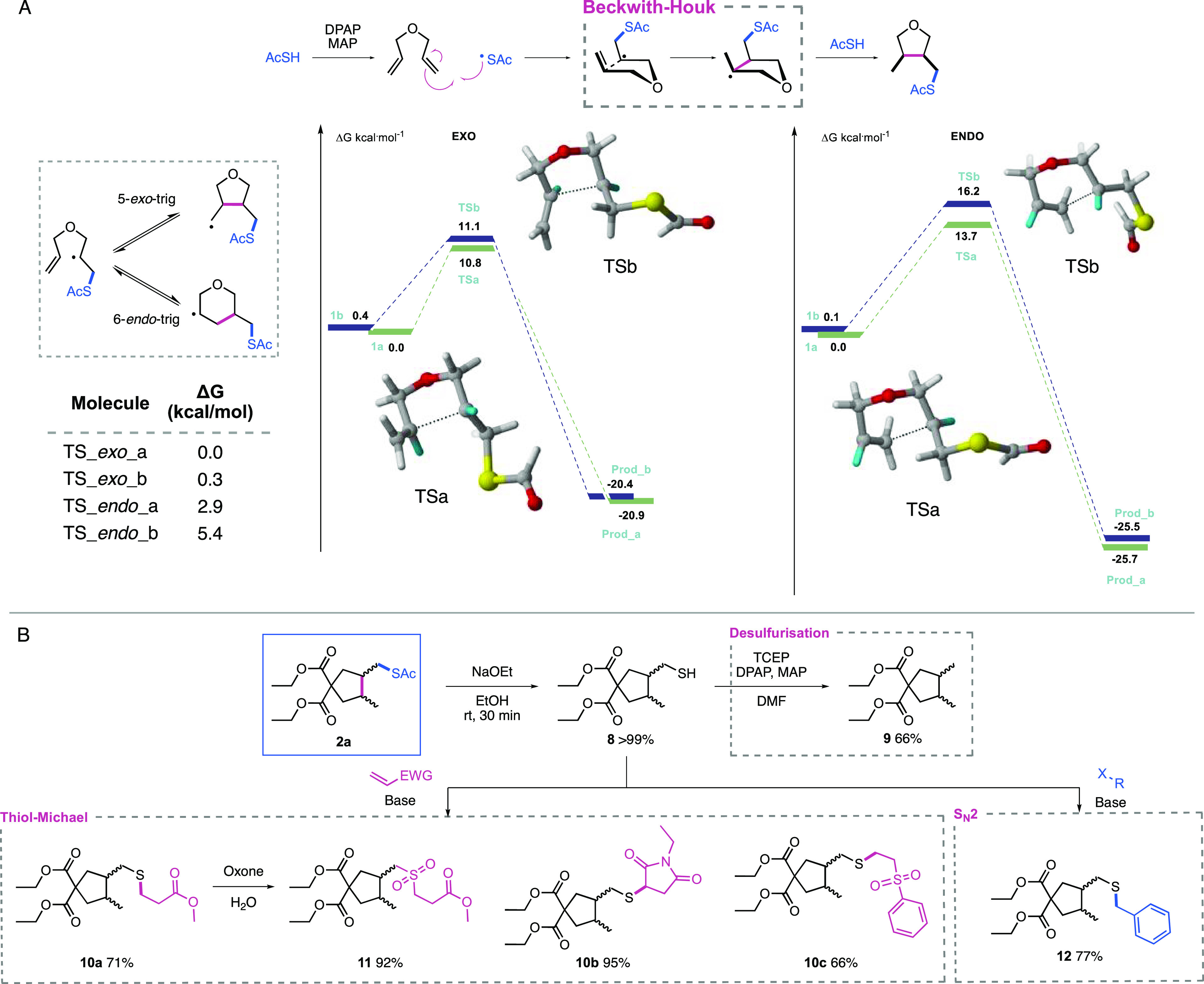
(A) Selectivity of 1,6-diene cyclizations. (B) Further diversification via the thiol handle.

The diastereoselectivities observed in cyclizations of hex-5-enyl radicals such as those investigated are often explained using the Beckwith–Houk model (Figure 2), which invokes a chairlike transition state in which substituents preferentially adopt pseudo-equatorial positions.^21−23^ DFT studies have since verified this model for protected N-substituted systems.^24^ Exo ring closure through this chairlike conformation then yields the *cis* product. Additionally, computational investigation into the observed *exo* selectivities was conducted for the thioacid-mediated system. The potential energy surface (PES) shows the lowest energy for the starting material conformation corresponding to the *trans exo* product, although the *cis* analogue lies only 0.4 kcal/mol higher. However, ΔG for the *cis* TS is marginally lower than that of the *trans* TS with respect to their starting conformations, at 10.7 and 10.8 kcal/mol, respectively. TS energies for the *endo* cyclization are significantly higher in both *cis* and *trans* cases. While the *endo* products are the more stable thermodynamic products, the *exo* products are the kinetic products in the case of this reaction. This can be attributed to the starting conformation for the *exo* more readily geometrically facilitating pre-TS assembly.

Another key advantage of this methodology for generation of 5-membered rings is in the potential for further derivatization via the exocyclic sulfur atom (Figure 2). Facile deacetylation to cleave the thioester furnishes a thiol handle of great synthetic potential for either further modification of the 5-membereed ring or conjugation to other moieties. The thiol handle could then be exploited without any need for chromatographic purification. We investigated the desulfurization of these cyclized thiol products, furnishing the cyclized diene in a traceless manner. Photochemical desulfurization of thiol **8** with tris(2-carboxyethyl)phosphine hydrochloride (TCEP) gave traceless cyclic product **9** in 66% yield, amounting to a three-step 65% yield in traceless thioacid-mediated 1,6-diene cyclization. Such desulfurative conditions have been applied to generation of C-centered radicals for C–C bond formation,^25^ another application to which these cyclic thiol products are suited.

We then demonstrated these thiols as nucleophiles in two common classes of reaction; thiol-Michael and S_N_2. The simple Michael acceptor methyl acrylate proceeded with a good yield of 71% (**10a**). This was then readily converted to the corresponding sulfone **11** using oxone with a high yield of 92%, amounting to a 64% overall yield for the four steps from diene to sulfone. As a result, this cyclization-deacylation approach has facilitated the installation of further functionality, followed by conversion to a highly medicinally relevant sulfone. Maleimides represent another acceptor system commonly used in cysteine modification in peptides and proteins. Addition of thiol **8** to *N*-ethyl maleimide proceeded with excellent yield of 95% (**10b**). A further class of Michael acceptors includes vinyl sulfones, and reaction with phenyl vinyl sulfone proceeded with a yield of 66% (**10c**). S_N_2 reaction of thiol **8** with benzyl bromide demonstrated an additional route for further functionalization, proceeding with a good yield of 77% (**12**).

## Conclusions

In conclusion, we have reported extensive study into the thioacid-mediated 5-*exo*-trig selective cyclization of 1,6-dienes. We have established the effects of varying alkene substitution on the yield of the cyclization reaction and investigated the origin of the observed *exo* selectivity via computational studies. We have demonstrated that through facile hydrolysis of the thioester, the corresponding thiol can be utilized for further derivatization or to access traceless cyclized products, offering facinating prospects for diversity-oriented synthesis and drug discovery.

## Experimental Section

### General Procedure

Commercial materials were obtained from Sigma-Aldrich, Fluorochem, Alfa-Aesar, or Fisher Scientific and used without further purification. Chromatographic separation was performed on Silica gel Florisil (200 mesh; Aldrich). Thin-layer chromatography (TLC) was performed on Merck 60 F254 silica gel plates and visualized by UV light, molybdenum, ninhydrin, or sulfuric acid staining. Dry solvents were obtained from a Pure Solv Micro Solvent Purification System. Deuterated solvents for use in NMR were purchased from Apollo Scientific. NMR data were obtained using a Bruker Advance 400 spectrometer and Bruker Ultrashield 600 and processed using Bruker TopSpin software. ESI mass spectra were acquired using a Bruker micrOTOF-Q III spectrometer interfaced to a Dionex UltiMate 3000 LC in positive and negative modes as required. The instrument was calibrated using a tune mix solution (Agilent Technologies ESI-l Low concentration tuning mix); this was also used as an internal lock mass. Masses were recorded over the range 100–2000 *m/z*. Operating conditions were as follows: end-plate offset 500 V capillary 4500 V, nebulizer 2.0Bar, dry gas 8.0 L/min, and dry temperature 180 °C. MicroTof control 3.2 and HyStar 3.2 software were used to carry out the analysis. UV reactions were performed in a Luzchem LZC-EDU (110 V/60 Hz) photoreactor housing 12 UV lamps centered at 365 nm. Reactions were performed in borosilicate glass and were centered in the reactor (approx. 10 cm from walls of the reactor).

### General Procedure for Thioacid-Initiated 1,6-Diene Cyclization

To a 0.1 M solution of diene (1.0 equiv), DPAP (0.1 equiv), and MAP (0.1 equiv) in EtOAc, AcSH (1.2 equiv) was added. The mixture was then irradiated at 365 nm for 2 h and then concentrated in vacuo. The product was then purified by silica gel flash chromatography.

#### (**2a**) Diethyl-3-((acetylthio)methyl)-4-methylcyclopentane-1,1-dicarboxylate

Prepared following the general procedure from diethyl diallylmalonate (0.298 g, 1.24 mmol) and purified by silica gel flash chromatography in Hex/EtOAc (10%) to yield the product as a colorless oil (0.385 g, 98%). *R*_f_ = 0.23 (10% Hex/EtOAc). ^1^H NMR (400 MHz, CDCl_3_): δ 4.19–4.14 (m, 4H), 2.94 (dd, *J* = 13.2, 6.5 Hz, 1H), 2.78 (dd, *J* = 13.2, 6.5 Hz, 1H), 2.47–2.37 (m, 2H), 2.32 (s, 3H), 2.27–2.14 (m, 2H), 2.09–2.04 (m, 1H), 2.01–1.96 (m, 1H), 1.26–1.20 (t, *J* = 7.1 Hz, 6H), 1.04 (d, *J* = 5.9 Hz, 0.33H), 0.92 (d, *J* = 6.9 Hz, 2.67H). ^13^C{^1^H} NMR (151 MHz, CDCl_3_): δ 195.8, 172.8, 172.7, 61.6, 58.9, 42.6, 41.3, 38.3, 36.2, 30.8, 29.9, 14.9, 14.2. HRMS: (ESI^+^) *m/z* calcd. For C_15_H_25_O_5_S ([M + H]^+^): 317.1417, found 317.1420.

#### (**2b**) *S*-((4-Methyltetrahydrofuran-3-yl)methyl)ethanethioate

Prepared following the general procedure from diallyl ether (0.200 g, 2.04 mmol) and purified by silica gel flash chromatography in Hex/EtOAc (2–5%) to yield the product as a colorless oil (0.212 g, 60%). *R*_f_ = 0.47 (20% Hex/EtOAc (v/v)). ^1^H NMR (400 MHz, CDCl_3_): δ 4.04–3.95 (m, 2H), 3.53–3.47 (m, 1H), 3.38–3.33 (m, 1H), 3.14–3.09 (m, 1H), 2.88 (dd, *J* = 13.5, 7.8 Hz, 1H), 2.37 (s, 3H), 2.05–1.95 (m, 2H), 1.09 (d, *J* = 6.5 Hz, 3H). ^13^C{^1^H} NMR (151 MHz, CDCl_3_) δ 195.5, 75.2, 72.8, 46.8, 39.5, 31.2, 30.6, 16.9. HRMS: (APCI) *m/z* calcd. For C_8_H_15_O_2_S ([M + H]^+^): 175.0787, found 175.0796 υ_max_ (ATR/cm^–1^): 2971 (C–H stretch), 2933 (C–H stretch), 1689 (C=O stretch).

#### (**2c**) *S*-((1-Acetyl-4-methylpyrrolidin-3-yl)methyl)ethanethioate

Prepared following the general procedure from 13a (0.0557 g, 0.40 mmol) and purified by silica gel flash chromatography in EtOAc to yield the product as a colorless oil (rotamers of diastereomers, 0.0761 g, 89%). *R*_f_ = 0.08 (EtOAc). ^1^H NMR (400 MHz, CDCl_3_): δ 3.85–3.80 (m, 0.32 H), 3.71–3.52 (m, 1.60 H), 3.35–2.98 (m, 3H), 2.90–2.80 (m, 1H), 2.49–2.36 (m, 4H), 2.11–1.85 (m, 4H), 1.14–1.11 (m, 1H), 1.06–1.01 (m, 2H). ^13^C{^1^H} NMR (151 MHz, CDCl_3_): δ 195.3, 195.2, 169.5, 169.4, 169.1, 54.5, 54.2, 52.6, 52.3, 52.1, 50.6, 50.4, 48.9, 46.1, 44.4, 42.4, 40.6, 38.5, 37.1, 35.8, 34.3, 30.7, 30.7, 30.6, 30.1, 29.1, 27.9, 27.8, 22.3, 22.2, 22.1, 22.1, 16.2, 16.1, 13.3, 13.3. HRMS: (ESI^+^) *m/z* calcd. For C_10_H_17_NO_2_SNa ([M + Na]^+^): 238.0872, found 238.0874. υ_max_ (ATR/cm^–1^): 2963 (C–H stretch), 1687 (C=O stretch), 1625 (C=O stretch).

#### (**2d**) *S*-((4-Methyl-1-(2,2,2-trifluoroacetyl)pyrrolidin-3-yl)methyl)ethanethioate

Prepared following the general procedure from 13d (0.0773 g, 0.40 mmol) and purified by silica gel flash chromatography in DCM to yield the product as a colorless oil (rotamers of diastereomers, 0.1000 g, 93%). *R*_f_ = 0.60 (DCM). ^1^H NMR (400 MHz, CDCl_3_): δ 3.97–3.65 (m, 2H), 3.51–2.98 (m, 3H), 2.90–2.81 (m, 1H), 2.54–2.38 (m, 4H), 2.14–1.94 (m, 1H), 1.16 (*app*-q, 1H), 1.06 (dd, *J* = 7.0, 2.1 Hz, 2H). ^13^C{^1^H} NMR (151 MHz, CDCl_3_): δ 195.0, 194.9, 155.9, 155.6, 116.3 (q, *J* = 287.4 Hz), 54.1, 53.9, 53.3, 53.2, 53.2, 51.9, 50.4, 49.5, 49.4, 46.1, 43.4, 42.4, 39.7, 38.6, 35.9, 33.2, 30.6, 29.6, 29.4, 27.6, 27.3, 15.9, 15.7, 13.1, 12.9. HRMS: (ESI^+^) *m/z* calcd. For C_10_H_14_F_3_NO_2_SNa ([M + Na]^+^): 292.0590, found 292.0591. υ_max_ (ATR/cm^–1^): 2966 (C–H stretch), 1684 (C=O stretch).

#### (**2e**) *S*-((1-(2-Chloroacetyl)-4-methylpyrrolidin-3-yl)methyl)ethanethioate

Prepared following the general procedure from 13e (0.0695 g, 0.40 mmol) and purified by silica gel flash chromatography in DCM/MeOH (0–5%) to yield the product as a colorless oil (rotamers of diastereomers, 0.0973 g, 98%). *R*_f_ = 0.60 (5% DCM/MeOH (v/v)). ^1^H NMR (400 MHz, CDCl_3_): δ 4.03–3.99 (m, 2H), 3.90–2.80 (m, 8H), 2.90–2.81 (m, 1H), 2.57–2.31 (m, 5H), 2.17–1.78 (m, 1H), 1.14 (t, *J* = 6.7 Hz, 1H), 1.05 (*app*-t, 2H). ^13^C{^1^H} NMR (151 MHz, CDCl_3_): δ 195.2, 195.2, 165.1, 164.8, 53.7, 53.4, 53.3, 53.0, 51.2, 51.1, 49.9, 49.6, 46.2, 44.1, 42.6, 41.8, 41.7, 41.7, 40.6, 40.3, 38.6, 36.7, 36.0, 34.0, 30.7, 29.9, 29.7, 27.8, 27.6, 16.1, 10.4, 13.2, 13.2. HRMS: (ESI^+^) *m/z* calcd. For C_10_H_16_ClNO_2_SNa ([M + Na]^+^): 272.0482, found 272.0485. υ_max_ (ATR/cm^–1^): 2963 (C–H stretch), 1686 (C=O stretch), 1646 (C=O stretch).

#### (**2f**) *tert*-Butyl-3-((acetylthio)methyl)-4-methylpyrrolidine-1-carboxylate

Prepared following the general procedure from 13b (0.0789 g, 0.40 mmol) and purified by silica gel flash chromatography in Hex/EtOAc (0–15%) to yield the product as a colorless oil (rotamers of diastereomers, 0.0737 g, 68%). *R*_f_ = 0.17 (10% EtOAc/Hexane (v/v)). ^1^H NMR (400 MHz, CDCl_3_): δ 3.64–3.59 (m, 0.75H), 3.52–4.43 (m, 1.25H), 3.19–3.11 (m, 1.55H), 3.02–2.79 (m, 2.55H), 2.40–2.29 (m, 4.17H), 1.98–1.92 (m, 0.79H), 1.48 (s, 5.70H), 1.47 (s, 3.30H), 1.09 (d, *J* = 6.20 Hz, 1.11H), 1.01 (d, *J* = 6.80, 1.81 Hz, 1.81H). ^13^C{^1^H} NMR (151 MHz, CDCl_3_): δ 195.4, 195.3, 154.7, 154.4, 79.2, 53.0, 52.7, 50.7, 49.2, 30.6, 30.3, 28.5 28.0, 16.3, 13.3. HRMS: (ESI^+^) *m/z* calcd. For C_13_H_23_NO_3_SNa ([M + Na]^+^): 296.1291, found 296.1294. υ_max_ (ATR/cm^–1^): 2972 (C–H stretch), 1687 (C=O stretch), 1131 (C–O stretch).

#### (**2g**) *S*-((4-Methy-1-tosylpyrrolidin-3-yl)methyl)ethanethioate

Prepared following the general procedure from 13c (0.2000 g, 0.79 mmol) and purified by silica gel flash chromatography in Hex/EtOAc (10%) to yield the product as a pale yellow oil (0.2330 g, 90%). *R*_f_ = 0.34 (10% Hex/EtOAc (v/v)). ^1^H NMR (400 MHz, CDCl_3_): δ 7.69 (dd, *J* = 8.2, 2.6 Hz, 2H), 7.31 (dd, *J* = 8.4, 2.2 Hz 2H), 3.53–3.32 (m, 2H), 3.09–2.74 (m, 3H), 2.62 (m, 1H), 2.42 (d, *J* = 2.5 Hz, 3H), 2.30 (d, *J* = 4.2 Hz, 3H), 2.28–2.11 (m, 1H), 1.90–1.74 (m, 1H), 0.81 (d, *J* = 6.5 Hz, 3H). ^13^C{^1^H} NMR (151 MHz, CDCl_3_): δ 195.2, 143.6, 134.0, 133.8, 129.8, 127.6, 127.5, 54.8, 54.5, 41.9, 35.6, 30.7, 27.8, 21.6, 13.2. HRMS: (ESI^+^) *m/z* calcd. For C_13_H_23_NO_3_SNa ([M + Na]^+^): υ_max_ (ATR/cm^–1^): 2884 (C–H stretch), 1688 (C=O stretch).

#### (**2h**) Diethyl-3-((acetylthio)methyl)-4-ethylcyclopentane-1,1-dicarboxylate

Prepared following the general procedure from 13g (0.2540 g, 1.00 mmol) and purified by silica gel flash chromatography in hex/EtOAc (2–5%) to yield the product as a colorless oil (0.2040 g, 62%). *R*_f_ = 0.28 (10% Hex/EtOAc (v/v)). ^1^H NMR (400 MHz, CDCl_3_): δ 4.23–4.10 (m, 4H), 3.02 (dd, *J* = 13.2, 5.4 Hz, 1H), 2.62 (dd, *J* = 13.2, 10.1 Hz, 1H), 2.53–2.49 (m, 1H), 2.42–2.28 (m, 5H), 2.28–2.17 (m, 1H), 2.12 (dd, *J* = 13.6, 6.1 Hz, 1H), 2.07–1.89 (m, 2H), 1.52–1.37 (m, 1H), 1.36–1.15 (m, 7H), 0.97–0.83 (m, 3H). ^13^C{^1^H} NMR (151 MHz, CDCl_3_): δ 195.8, 172.8, 61.6, 58.8, 44.5, 41.8, 38.7, 38.7, 30.7, 29.6, 22.2, 14.1, 12.8. HRMS: (ESI^+^) *m/z* calcd. For C_16_H_26_O_5_SNa ([M + Na]^+^): 353.1399, found 353.1396. υ_max_ (ATR/cm^–1^): 2972 (C–H stretch), 2874 (C–H stretch), 1725 (C=O stretch), 1689.

#### (**2i**) Diethyl-3-((acetylthio)methyl)-4-isopropylcyclopentane-1,1-dicarboxylate

Prepared following the general procedure from 13h (0.2680 g, 1.00 mmol) and purified by silica gel flash chromatography in hex/EtOAc (2–5%) to yield the product as a colorless oil (0.2280 g, 66%). *R*_f_ = 0.41 (10% Hex/EtOAc (v/v)). ^1^H NMR (400 MHz, CDCl_3_): δ 4.26–4.08 (m, 4H), 3.17 (ddd, *J* = 13.0, 3.1, 1.5 Hz, 1H), 2.55–2.41 (m, 1H), 2.39–2.33 (m, 1H), 2.31 (s, 3H), 2.29–2.21 (m, 1H), 1.99–1.83 (m, 1H), 1.73–1.52 (m, 1H), 1.23 (td, *J* = 7.1, 3.9 Hz, 6H), 1.04 (d, *J* = 6.2 Hz, 3H), 0.94 (dd, *J* = 6.2 Hz, 3H). ^13^C{^1^H} NMR (151 MHz, CDCl_3_): δ 196.1, 173.0, 61.7, 58.5, 52.0, 41.0, 38.6, 37.2, 30.7, 29.1, 28.8, 22.2, 21.8, 14.2. HRMS: (ESI^+^) *m/z* calcd. For C_17_H_28_O_5_SNa ([M + Na]^+^): 367.1555, found 367.1541. υ_max_ (ATR/cm^–1^): 2963 (C–H stretch), 2160 (C–H stretch), 1722 (C=O stretch), 1683.

#### (**2j**) Diethyl-3-(1-(acetylthio)ethyl)-4-ethylcyclopentane-1,1-dicarboxylate

Prepared following the general procedure from 13i (0.2680 g, 1.00 mmol) and purified by silica gel flash chromatography in hex/EtOAc (2–5%) to yield the product as a colorless oil (0.1970 g, 57%). *R*_f_ = 0.33 (10% Hex/EtOAc (v/v)). ^1^H NMR (400 MHz, CDCl_3_): δ 4.25–4.07 (m, 4H), 3.63–3.41 (m, 1H), 2.55–2.32 (m, 2H), 2.32–2.27 (m, 3H), 2.18 (dtd, *J* = 14.1, 7.2, 1.1 Hz, 1H), 2.13–2.01 (m, 1H), 1.98–1.88 (m, 2H), 1.36–1.30 (m, 3H), 1.30–1.18 (m, 6H), 0.96–0.80 (m, 3H). ^13^C{^1^H} NMR (151 MHz, CDCl_3_): δ 195.7, 172.7, 61.6, 58.4, 48.8, 43.1, 40.4, 37.6, 37.4, 36.9, 31.0, 30.9, 22.1, 21.6, 14.1, 12.3. HRMS: (ESI^+^) *m/z* calcd. For C_17_H_28_O_5_SNa ([M + Na]^+^): 367.1550, found 367.1544. υ_max_ (ATR/cm^–1^): 2968 (C–H stretch), 2871 (C–H stretch), 1726 (C=O stretch), 1688.

#### (**2k**) Diethyl-3-(2-(acetylthio)propan-2-yl)-4-isopropylcyclopentane-1,1-dicarboxylate

Prepared following the general procedure from 13j (0.2960 g, 1.00 mmol) and purified by silica gel flash chromatography in hex/EtOAc (2–5%) to yield the product as a colorless oil (0.1730 g, 47%, inseparable mixture of products). *R*_f_ = 0.39 (10% Hex/EtOAc (v/v)). ^1^H NMR (400 MHz, CDCl_3_): δ 4.21–4.05 (m, 4H), 2.74–2.60 (m, 1H), 2.42 (ddd, *J* = 13.5, 3.9, 2.5 Hz, 1H), 2.34 (s, 3H) 2.17–2.09 (m, 2H), 2.08–1.90 (m, 2H), 1.71–1.55 (m, 1H), 1.33–1.19 (m, 6H), 1.04–0.76 (m, 12H). ^13^C{^1^H} NMR (151 MHz, CDCl_3_): δ 194.2, 170.5, 61.7, 55.8, 51.4, 48.5, 34.9, 30.9, 30.6, 26.7, 26.6, 24.6, 18.4, 16.9, 14.2, 14.0. HRMS: (ESI^+^) *m/z* calcd. For C_19_H_32_O_5_SNa ([M + Na]^+^): 395.1863, found, 395.1878. υ_max_ (ATR/cm^–1^): 2964 (C–H stretch), 1727 (C=O stretch), 1693.

#### (**2l**) Diethyl-4-((acetylthio)methyl)-3-methyl-3-phenylcyclopentane-1,1-dicarboxylate

Prepared following the general procedure from 13l (0.3160 g, 1.00 mmol) and purified by silica gel flash chromatography in hex/EtOAc (2–5%) to yield the product as a colorless oil (0.1374 g, 35%). *R*_f_ = 0.30 (10% Hex/EtOAc (v/v)). ^1^H NMR (400 MHz, CDCl_3_): δ 7.37–7.27 (m, 3H), 7.24–7.17 (m, 2H), 4.29–4.09 (m, 4H), 3.42 (dd, *J* = 13.4, 1.3 Hz, 1H), 3.23 (dd, *J* = 16.3, 13.4 Hz, 1H), 3.03–2.86 (m, 1H), 2.73–2.59 (m, 1H), 2.58–2.48 (m, 1H), 2.47–2.24 (m, 2H), 2.25–2.19 (m, 3H), 1.35–1.13 (m, 6H), 1.01 (d, *J* = 6.8 Hz, 3H). ^13^C{^1^H} NMR (151 MHz, CDCl_3_): δ 195.2, 172.8, 172.3, 144.7, 128.4, 128.1, 127.9, 126.5, 61.8, 58.1, 52.4, 45.7, 45.0, 41.1, 34.0, 30.7, 30.6, 14.2. HRMS: (ESI^+^) *m/z* calcd. For C_21_H_28_O_5_SNa ([M + Na]^+^): 415.1555, found 415.1553. υ_max_ (ATR/cm^–1^): 2979 (C–H stretch), 1724 (C=O stretch), 1690.

#### (**2m**) Diethyl-3-((acetylthio)methyl)-4-benzylcyclopentane-1,1-dicarboxylate

Prepared following the general procedure from 13k (0.1530 g, 0.48 mmol) and purified by silica gel flash chromatography in hex/EtOAc (2–5%) to yield the product as a colorless oil (0.0262 g, 14%). *R*_f_ = 0.16 (5% Hex/EtOAc (v/v)). ^1^H NMR (400 MHz, CDCl_3_): δ 7.28 (dd, *J* = 8.3, 7.0 Hz, 2H), 7.21–7.18 (m, 3H), 4.22–4.10 (m, 4H), 3.11 (dd, *J* = 13.2, 6.3 Hz, 1H), 2.86 (dd, *J* = 13.2, 9.5 Hz, 1H), 2.80 (d, *J* = 8.4 Hz, 1H), 2.48–2.39 (m, 3H), 2.34 (d, *J* = 2.0 Hz, 3H), 2.33–2.27 (m, 1H), 2.27–2.15 (m, 2H), 2.14–2.03 (m, 1H), 1.29–1.20 (m, 9H). ^13^C{^1^H} NMR (151 MHz, CDCl_3_): δ 195.7, 172.8, 172.6, 140.7, 122.9, 128.4, 126.0, 61.6, 61.5, 61.4 60.0, 58.5, 43.9, 42.4 39.7, 39.5, 38.5, 37.8, 35.0, 33.6, 33.5, 31.4, 30.6, 29.6, 14.2, 14.1. HRMS: (ESI^+^) *m/z* calcd. For C_21_H_28_O_5_SNa ([M + Na]^+^): 415.1555, found 415.1553. υ_max_ (ATR/cm^–1^): 2979 (C–H stretch), 1723 (C=O stretch).

#### (**3a**) Diethyl-3-((heptanoylthio)methyl)-4-methylcyclopentane-1,1-dicarboxylate

Prepared following the general procedure from diethyl diallylmalonate (0.150 mL, 0.1489 g 0.62 mmol) and crude thioacid obtained via the general procedure for *S*-trityl deprotection from the trityl thioester 17a (0.2860 g, 0.74 mmol). The product was purified by silica gel flash chromatography in hex/EtOAc (2–5%) to yield the product as a colorless oil (0.1500 g, 65%). *R*_f_ = 0.31 (4% Hex/EtOAc (v/v)). ^1^H NMR (400 MHz, CDCl_3_): δ 4.17 (m, 4H), 2.94 (dd, *J* = 13.2, 6.5 Hz, 2H), 2.78 (dd, *J* = 13.2, 8.6 Hz, 2H), 2.56–2.50 (m, 4H), 2.48–2.35 (m, 4H), 2.28–2.13 (m, 4H, CH2), 2.07 (dd, *J* = 13.2, 8.6 Hz, 2H), 1.99 (dd, *J* = 13.8, 5.7 Hz, 2H), 1.64 (dt, *J* = 15.0, 7.7 Hz, 4H), 1.36–1.20 (m, 12H), 1.04 (d, *J* = 5.9 Hz, 3H), 0.92 (d, *J* = 6.9 Hz, 6H), 0.88 (t, *J* = 6.9 Hz, 3H). ^13^C{^1^H} NMR (151 MHz, CDCl_3_): δ 199.6, 172.7, 61.6, 58.9, 44.3, 42.7, 41.3, 38.3, 36.2, 31.6, 29.5, 28.8, 25.8, 22.6, 14.9, 14.2. HRMS: (APCI) *m/z* calcd. For C_20_H_35_O_5_S ([M + H]^+^): 387.2205, found 387.2199. υ_max_ (ATR/cm^–1^): 2958 (C–H stretch), 2931 (C–H stretch), 2873 (C–H stretch), 1729 (C=O), 1690 (C=O).

#### (**3b**) Diethyl-3-(((3-ethoxypropanoyl)thio)methyl)-4-methylcyclopentane-1,1-dicarboxylate

Prepared following the general procedure from diethyl diallylmalonate (80 μL, 0.0793 g, 0.33 mmol) and crude thioacid obtained via the general procedure for S-trityl deprotection from the trityl thioester 17b (0.1505 g, 0.4 mmol). The product was purified by silica gel flash chromatography in hex/EtOAc (2–10%) to yield the product as a yellow oil (0.0991 g, 80%). *R*_f_ = 0.31 (10% Hex/EtOAc (v/v). ^1^H NMR (400 MHz, CDCl_3_): δ 4.13–4.20 (m, 4H), 3.71 (t, *J* = 6.5 Hz, 2H), 3.48 (q, 7.0 Hz, 2H), 2.96 (dd, *J* = 13.6, 6.6 Hz, 1H), 2.78–2.84 (m, 3H), 2.37–2.47 (m, 2H), 2.14–2.27 (m, 2H), 2.04–2.10 (m, 1H), 1.98 (dd, *J* = 13.9, 5.7 Hz, 1H), 1.21–1.25 (dt, *J* = 7.1, 2.0 Hz, 6H), 1.18 (t, *J* = 7.1 Hz, 3H), 1.04 (d, *J* = 5.9 Hz, 3H), 0.91 (d, *J* = 6.9 Hz, 3H).^13^C{^1^H} NMR (151 MHz, CDCl_3_): δ 197.3, 172.6, 66.5, 65.9, 61.5, 58.9, 44.4, 42.4, 41.2, 38.2, 36.1, 29.5, 15.1, 14.8, 14.0. HRMS: (ESI) *m/z* calcd. For C_18_H_30_NaO_6_S ([M + Na]^+^): 397.1655, found 397.1666. υ_max_ (ATR/cm^–1^): 2976 (C–H stretch), 1728 (C=O), 1689 (C=O), 1253 (C–O stretch).

#### (**3c**) Diethyl-3-((benzoylthio)methyl)-4-methylcyclopentane-1,1-dicarboxylate

Prepared following the general procedure from diethyl diallylmalonate (0.30 mL, 0.298 g, 1.24 mmol) and thiobenzoic acid (0.17 mL, 0.205 g, 1.49 mmol). The product was purified by silica gel flash chromatography in Hex/EtOAc (5% v/v) to yield the product as a pale yellow oil (0.190 g, 40%). *R*_f_ = 0.34 (10% Hex/EtOAc (v/v)). ^1^H NMR (400 MHz, CDCl_3_): δ 7.98 (t, *J* = 8.0 Hz, 2H), 7.49 (tt, *J* = 7.4, 1.3 Hz, 1H), 7.47 (t, *J* = 7.7 Hz, 2H), 4.17–4.24 (m, 4H), 3.15–3.41 (m, 1H), 2.96–3.04 (m, 1H), 2.46–2.64 (m, 2H), 2.29–2.38 (m, 2H), 2.19 (dd, *J* = 13.5, 8.8 Hz, 1 H), 2.05 (dd, *J* = 13.7, 5.4 Hz, 1H), 1.24–1.28 (m, 6H), 1.01 (d, *J* = 6.8 Hz, 3H). ^13^C{^1^H} NMR (151 MHz, CDCl_3_): δ 191.8, 172.65, 172.63, 137.1, 133.3, 128.6, 127.2, 61.5, 58.9, 42.5, 41.2, 38.2, 36.2, 29.6, 17.8, 14.9, 14.0. HRMS: (APCI) *m/z* calcd. For C_20_H_27_O_5_S ([M + H]^+^): 379.1574, found 379.1576. υ_max_ (ATR/cm^–1^): 2977 (C–H stretch), 1727 (C=O), 1662 (C=O), 1581 (Ar C–C stretch).

#### (**3d**) Diethyl-3-methyl-4-(((2-(p-tolyl)acetyl)thio)methyl)cyclopentane-1,1-dicarboxylate

Prepared following the general procedure from diethyl diallylmalonate (0.150 mL, 0.1489 g 0.62 mmol) and crude thioacid obtained via the general procedure for *S*-trityl deprotection from the trityl thioester 17c (0.3150 g, 0.74 mmol). The product was purified by silica gel flash chromatography in hex/EtOAc (10%) to yield the product as a colorless oil (0.1500 g, 65%). *R*_f_ = 0.27 (10% Hex/EtOAc (v/v)). ^1^H NMR (400 MHz, CDCl_3_): δ 7.11–7.05 (m, 4H), 4.17 (qd, *J* = 7.2, 2.3 Hz, 4H), 2.96–2.91 (m, 3H,), 2.86–2.76 (m, 3H), 2.47–2.36 (m, 2H), 2.31 (s, 3H), 2.25–2.12 (m, 2H), 2.06 (dd, *J* = 13.3, 8.7 Hz, 1H), 1.99 (dd, *J* = 13.8, 5.7 Hz, 1H), 1.24 (td, *J* = 7.2, 1.2 Hz, 6H), 0.91 (d, *J* = 6.9 Hz, 3H). ^13^C{^1^H} NMR (151 MHz, CDCl_3_): δ 198.6, 172.7, 129.3, 128.3, 61.6, 58.9, 45.8, 42.6, 41.3, 38.3, 36.2, 31.2, 29.6, 21.1, 14.9, 14.2. HRMS: (APCI) *m/z* calcd. For C_23_H_33_O_5_S ([M + H]^+^): 421.2049, found 421.2049. υ_max_ (ATR/cm^–1^): 2963 (C–H stretch), 1723 (C=O stretch).

#### (**3e**) Diethyl-3-((((((9*H*-fluoren-9-yl)methoxy)carbonyl)glycyl)thio)methyl)-4-methylcyclopentane-1,1-dicarboxylate

Prepared following the general procedure from diethyl diallylmalonate (0.150 mL, 0.1489 g 0.62 mmol) and crude thioacid obtained via the general procedure for *S*-trityl deprotection from the trityl thioester 17d (0.4140 g, 0.74 mmol). The product was purified by silica gel flash chromatography in hex/EtOAc (10%) to yield the product as a colorless oil (0.3000 g, 87%). *R*_f_ = 0.29 (10% Hex/EtOAc (v/v)). ^1^H NMR (400 MHz, CDCl_3_): δ 7.77 (d, *J* = 7.5 Hz, 2H), 7.61 (d, *J* = 7.4 Hz, 2H), 7.40 (t, *J* = 7.4 Hz, 2H), 7.32 (t, *J* = 7.2 Hz, 2H), 5.34 (br s, 1H), 4.44 (d, *J* = 7.0 Hz, 2H), 4.25 (t, *J* = 7.0 Hz, 1H), 4.20–4.11 (m, 6H), 3.02–2.97 (m, 1H, CH2), 2.87–2.82 (m, 1H), 2.48–2.37 (m, 2H), 2.28–2.15 (m, 2H), 2.11–2.05 (m, 1H), 2.01–1.96 (m, 1H), 1.27–1.20 (m, 6H), 0.92 (d, *J* = 6.9 Hz, 3H). ^13^C{^1^H} NMR (151 MHz, CDCl_3_): δ 197.4, 172.7, 172.7, 156.3, 143.9, 141.5, 127.9, 127.2, 125.2, 120.1, 67.5, 61.7, 58.9, 50.8, 47.3, 42.5, 41.3, 38.3, 36.3, 29.4, 14.9, 14.2. HRMS: (APCI) *m/z* calcd. For C_30_H_36_NO_7_S ([M + H]^+^): 554.2207, found 554.2209. υ_max_ (ATR/cm^–1^): 3315 (N–H stretch), 2936 (C–H stretch), 1711 (C=O).

## Data Availability

The data underlying this study are available in the published article and its Supporting Information.

## Supporting Information Available

General information; general synthetic procedures; diene substrate syntheses; trityl thioester syntheses; thioacid-mediated cyclization of 1,6-dienes; derivatization; computational work; references; and NMR spectra of novel compounds (PDF)
