# Supplementary material for: Traceless Thioacid-Mediated Radical Cyclization of 1,6-Dienes
Source: J Org Chem. 2023 Jul 7;88(14):10020–6. doi: 10.1021/acs.joc.3c00824 (PMC10367065; doi:10.1021/acs.joc.3c00824)

Traceless Thioacid Mediated Radical Cyclization of 1,6-Dienes.

Dylan M. Lynch,[‡a] Mark D. Nolan,[‡a] Conor Williams,[a] Leendert Van Dalsen,[†a] Susannah H. Calvert,[a] Fabrice Dénès[b] Cristina Trujillo[†a] and Eoin M. Scanlan*[a]

[a]Trinity Biomedical Sciences Institute, Trinity College Dublin, 152-160 Pearse Street, Dublin 2, Ireland.
[b]Université de Nantes, CEISAM UMR CNRS 6230 UFR des Sciences et des Techniques - 2 rue de la Houssinière, BP 92208 - 44322 NANTES Cedex 3, France.

‡These authors contributed equally

e-mail: eoin.scanlan@tcd.ie

Supporting Information

Contents:

General Information	S2
General Synthetic Procedures	S3
Diene Substrate Syntheses	S4
Trityl Thioester Synthesis	S11
Thioacid Mediated Cyclisation of 1,6-Dienes	S13
Derivatization	S14
Computational Work	S19
References	S27
NMR Spectra of Novel Compounds	S29

General Information

Commercial materials were obtained from Sigma-Aldrich, Fluorochem, Alfa-Aesar or Fisher Scientific and used without further purification. Chromatographic separation was performed on Silica gel Florisil (200 mesh; Aldrich). Thin-layer chromatography (TLC) was performed on Merck 60 F254 silica gel plates and visualized by UV light, molybdenum, ninhydrin, or sulfuric acid staining. Dry solvents were obtained from a Pure Solv Micro Solvent Purification System. Deuterated solvents for use in NMR were purchased from Apollo Scientific. NMR data was obtained using a Bruker Advance 400 spectrometer and Bruker Ultrashield 600 and processed using Bruker TopSpin software. ESI mass spectra were acquired using a Bruker micrOTOF-Q III spectrometer interfaced to a Dionex UltiMate 3000 LC in positive and negative modes as required. The instrument was calibrated using a tune mix solution, (Agilent Technologies ESI-l Low concentration tuning mix) this was also used as an internal lock mass. Masses were recorded over the range 100-2000 m/z. Operating conditions were as follows: end-plate offset 500V capillary 4500V, nebulizer 2.0Bar, dry gas 8.0 L/min, and dry temperature 180°C. MicroTof control 3.2 and HyStar 3.2 software were used to carry out the analysis. UV reactions were performed in a Luzchem LZC-EDU (110 V/ 60 Hz) photoreactor housing 12 UV lamps centered at 365 nm.

General Procedures:

Acetylation of diallylamine using anhydrides
A 1.0 M solution of diallylamine (1.0 equiv.) and TEA (1.0 equiv.) in THF was cooled to 0 ºC. Anhydride (1.1 equiv.) was added dropwise (in THF solution if solid). The mix was allowed warm to rt and stirred for 18 h, following which it was diluted with H_2O and extracted with DCM. The organics were washed with 0.1M HCl, sat. $NaHCO_3$ and H_2O, dried over $MgSO_4$ and concentrated *in vacuo*. Products were of sufficient purity without further purification.

α-Functionalization of Diethyl Allylmalonate
To a solution of Na metal in EtOH (1 M, 5 mL), diethyl allylmalonate (5.00 mmol, 1.0 equiv.) was added dropwise. The solution was cooled to 0 ºC and alkyl bromide (6.00 mmol, 1.2 equiv.) was added dropwise. The reaction was then brought to reflux using an oil bath and stirred for 2 h. The solution was then cooled and acidified using glacial acetic acid, then concentrated *in vacuo*. The product was then purified by silica gel flash chromatography.

Double α-Functionalization of Diethyl Malonate
To a solution of Na metal in EtOH (1 M, 5 mL), diethyl allylmalonate (5.00 mmol, 1.0 equiv.) was added dropwise. The solution was cooled to 0 ºC and alkyl bromide (12.00 mmol, 2.4 equiv.) was added dropwise. The reaction was then brought to reflux using an oil bath and stirred for 2 h. The solution was then cooled and acidified using glacial acetic acid, then concentrated *in vacuo*. The product was then purified by silica gel flash chromatography.

Thioacid-Initiated 1,6-Diene Cyclisation
To a 0.1M solution of diene (1.0 equiv.), DPAP (0.1 equiv.) and MAP (0.1 equiv.) in EtOAc, AcSH (1.2 equiv.) was added. The mix was then irradiated at 365 nm for 2 hours and then concentrated *in vacuo*. The product was then purified by silica gel flash chromatography.

***S*-Trityl Thioester Synthesis**
To a 0.1 M solution of carboxylic acid in anhydrous DCM under Ar, DMAP (0.1 equiv.), triphenylmethanethiol (1.0 equiv.) and EDC•HCl (2.0 equiv.) was added. The solution was then stirred under Ar for 18 h, following which it was concentrated *in vacuo* and then purified by silica gel flash chromatography.

***S*-Trityl Deprotection**
To a 0.1 M solution of trityl thioester in anhydrous DCM, TFA (final conc. 25% v/v in DCM) and ethyldimethylsilane (10 equiv.) was added, and the mixture stirred for 10 min. Toluene was then added, and the reaction concentrated *in vacuo*. Crude thioacids were used immediately.

***S*-Deacetylation**
To a 0.5 M solution of acetyl thioester in anhydrous ethanol, sodium (0.2 equiv., 21 wt % in ethanol) was added. The mix was stirred under Ar for 30 min, then quenched using freshly washed Amberlite H^+ resin, filtered and concentrated *in vacuo*.

Diene Substrate Syntheses

Diallylacetamide products are obtained as a mixture of rotamers, exemplified by variable temperature ^{1}HNMR analysis of compound **13a** (Fig. S1).

Figure S1: Variable temperature ^{1}H NMR (400 MHz, DMSO-d_6) analysis of *N,N*-diallylacetamide.

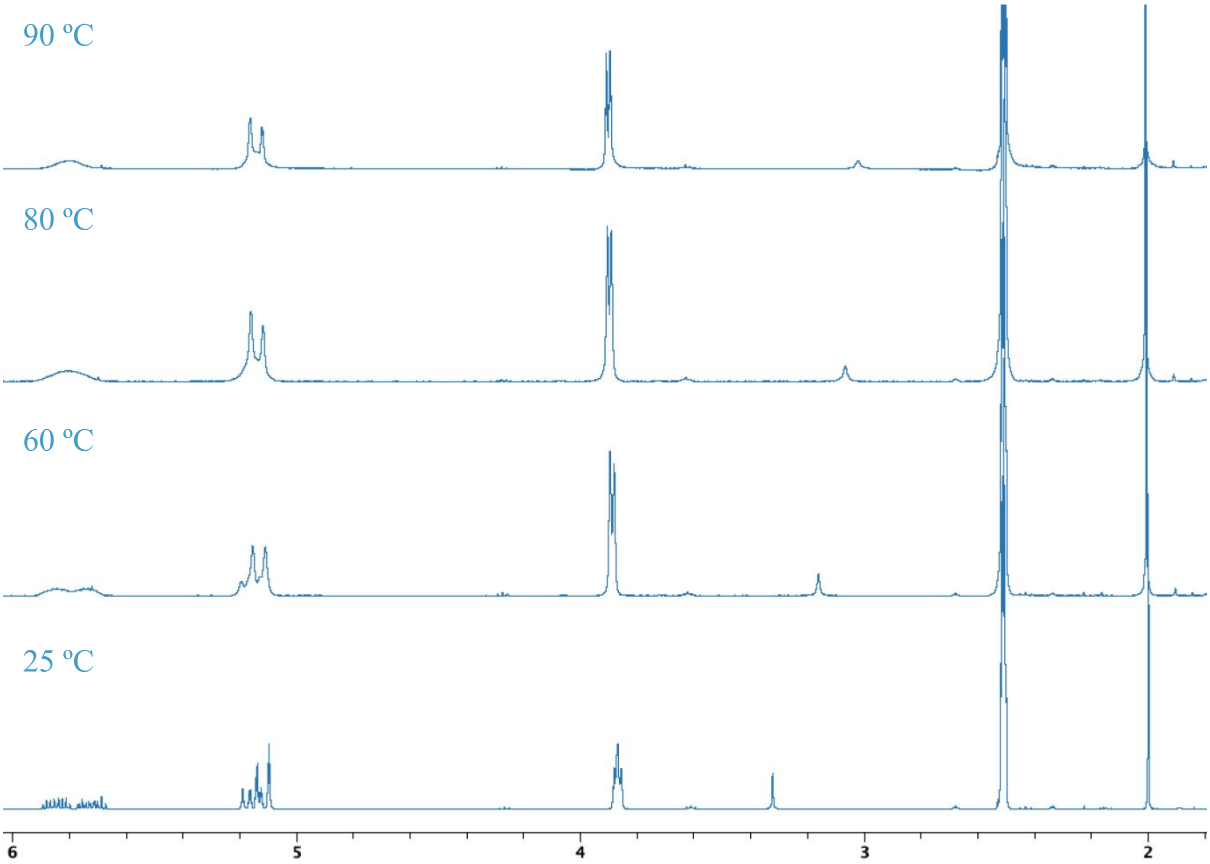

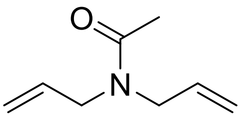

(13a) ***N,N*-diallylacetamide**[1]

Prepared following the general procedure from diallylamine (0.309 mL, 0.2429 g, 2.50 mmol), and acetic anhydride (0.260 mL, 0.2807 g, 2.75 mmol), to yield the desired as a colorless oil (0.3280 g, 94%).

^{1}H NMR (400 MHz, $CDCl_3$): δ 5.84-5.74 (m, 2H), 5.25-5.12 (m, 4H), 4.01 (d, J = 5.96, 2H), 3.89 (d, J = 4.88, 2H), 2.12 (s, 3H).

^{13}C{^{1}H} NMR (151 MHz, $CDCl_3$): δ 170.6, 133.3, 132.7, 117.2, 116.6, 50.0, 47.8, 21.4.

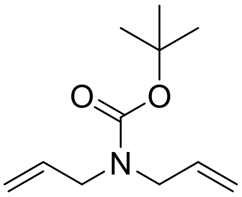

(13b) ***Tert*-butyl diallylcarbamate**[2]

Prepared following the general procedure from diallylamine (0.309 mL, 0.2429 g, 2.50 mmol), and di-*tert*-butyl dicarbonate (0.6002 g, 2.75 mmol), to yield the desired as a pale yellow oil (0.4932 g, >99%).

^{1}H NMR (400 MHz, $CDCl_3$): δ 5.83-5.74 (m, 2H), 5.15-5.11 (m, 4H), 3.80 (br s, 4H), 1.48 (s, 9H).

^{13}C{^{1}H} NMR (151 MHz, $CDCl_3$): δ 155.5, 134.0, 116.2, 79.6, 48.7, 28.4, 27.4.

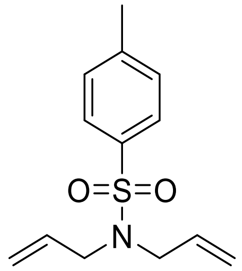

(13c) ***N,N*-diallyl-4-methylbenzenesulfonamide**[3]

To tosyl chloride (2.00 g, 10.48 mmol) and triethylamine (1.50 mL, 10.68 mmol), a solution of diallylamine (1.27 mL, 10.29 mmol) in DCM (35 mL) was added and the solution stirred at rt for 18 h. The organics were then washed with 10% $KHSO_4$, sat. $NaHCO_3$, and then H_2O, then dried over $MgSO_4$ and concentrated *in vacuo* to yield the product as a pale yellow oil (2.24 g, 86%).

^{1}H NMR (400 MHz, $CDCl_3$): δ 7.70 (d, J = 8.0 Hz, 2H), 7.29 (d, J = 8.0 Hz, 2H), 5.67-5.55 (m, 2H), 5.18-5.10 (m, 4H), 3.80 (d, J = 6.3 Hz, 4H), 2.43 (s, 3H).

HRMS: ($APCI^+$) m/z calcd. For $C_{13}H_{18}NOS$ ($[M+H]^+$): 252.1058, found 252.1053.

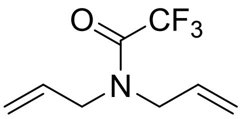

(13d) *N,N*-diallyl-2,2,2-trifluoroacetamide[4]

Prepared following the general procedure from diallylamine (0.123 mL, 0.0972 g, 1.0 mmol), and trifluoroacetic anhydride (0.139 mL, 0.2100 g, 1.10 mmol), to yield the desired as a pale yellow oil (0.1258 g, 65%).

^{1}H NMR (400 MHz, $CDCl_3$): δ 5.83-5.73 (m, 2H), 5.33-5.20 (m, 4H), 4.03 (m, 4H).

^{13}C{^{1}H} NMR (151 MHz, $CDCl_3$): δ 156.9, 156.7, 131.8, 130.9, 118.2 (q, J = 287.3 Hz), 49.1, 49.1, 48.2.

δ_F (377 MHz, $CDCl_3$) -72.3, -72.4, -72.4, -72.4

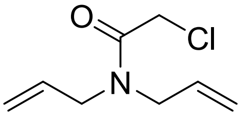

(13e) *N,N*-diallyl-2-chloroacetamide[5]

Prepared following the general procedure from diallylamine (0.123 mL, 0.0972 g, 1.0 mmol), and chloroacetic anhydride (0.1881 g, 1.10 mmol), to yield the desired as a pale yellow oil (0.1647 g, 95%).

^{1}H NMR (400 MHz, $CDCl_3$): δ 5.88-5.74 (m, 2H), 5.32-5.18 (m, 4H), 4.09 (s, 2H), 4.03 (d, J = 5.9 Hz, 2H), 3.99 (d, J = 4.9 Hz, 2H).

^{13}C{^{1}H} NMR (151 MHz, $CDCl_3$): δ 166.6, 132.6, 132.3, 117.9, 117.3, 49.5, 48.3, 41.1.

HRMS: (ESI^+) m/z calcd. For $C_8H_{12}NOClNa$ ($[M+Na]^+$): 196.0500, found 196.0502.

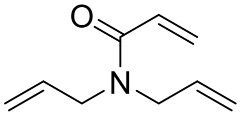

(13f) *N,N*-diallylacrylamide[6]

To a solution of diallylamine (0.62 mL, 5.00 mmol) in toluene (10 mL) at 0 ºC, triethylamine (0.84 mL, 6.00 mmol) and acryloyl chloride (0.49 mL, 6.00 mmol) was added. The solution was stirred for 3 h at 0 ºC, then filtered to remove precipitate, washed with ice-cold Et_2O and concentrated *in vacuo*. The product was purified by silica gel flash chromatography in Hex/EtOAc (20%) to yield the product as a colorless oil (0.316 g, 42%).

^{1}H NMR (600 MHz, $CDCl_3$): δ 6.66 (dd, J, = 16.7, 10.3 Hz, 1H), 6.17 (dd, J = 16.6, 2.5 Hz, 1H), 5.91-5.72 (m, 2H) 5.69 (dd, J = 10.3, 2.4 Hz, 1H), 5.22-5.08 (m, 4H), 3.98 (ddt, J = 23.8, 5.9, 1.7 Hz, 4H).

^{13}C{^{1}H} NMR (151 MHz, $CDCl_3$): δ 165.1, 134.2, 133.6, 128.3, 127.6, 117.0, 116.1, 48.8, 47.8.

HRMS: (ESI^+) m/z calcd. For $C_9H_{13}NONa$ ($[M+Na]^+$): 174.0889, found 174.0887.

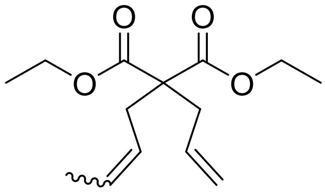

(13g) Diethyl 2-allyl-2-(but-2-en-1-yl)malonate[9]

Prepared following the general procedure from diethyl allylmalonate (0.99 mL, 5.00 mmol) and crotyl bromide (0.62 mL, 6.00 mmol), followed by purification by silica gel flash chromatography in Hex/EtOAc (5%) to yield the product as a pale yellow oil (0.660 g, 52%).

Rf = 0.46 (10% EtOAc/Hexane (v/v)).

^{1}H NMR (400 MHz, $CDCl_3$): δ 5.73-5.44 (m, 2H), 5.32-5.18 (m, 1H), 5.14-5.03 (m, 2H), 4.17 (q, J = 7.1 Hz, 4H), 2.68-2.52 (m, 4H), 1.67-1.57 (m, 3H), 1.28-1.19 (m, 6H).

HRMS: (ESI^+) m/z calcd. for $C_{14}H_{22}O_4Na$ ($[M+Na]^+$]): 277.1416, found 277.1410.

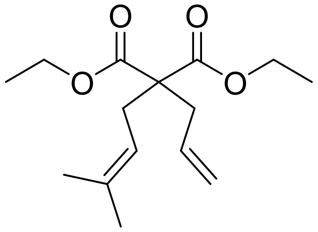

(13h) Diethyl 2-allyl-2-(3-methylbut-2-en-1-yl)malonate[9]

Prepared following the general procedure from diethyl allylmalonate (0.99 mL, 5.00 mmol) and prenyl bromide (0.69 mL, 6.00 mmol), followed by purification by silica gel flash chromatography in Hex/EtOAc (5%) to yield the product as a pale yellow oil (0.657 g, 49%).

Rf = 0.62 (10% EtOAc/Hexane (v/v)).

^{1}H NMR (400 MHz, $CDCl_3$): δ 5.73-5.44 (m, 2H), 5.32-5.18 (m, 1H), 5.14-5.03 (m, 2H), 4.17 (q, J = 7.1 Hz, 4H), 2.68-2.52 (m, 4H), 1.67-1.57 (m, 3H), 1.28-1.19 (m, 6H).

HRMS: (ESI^+) m/z calcd. for $C_{14}H_{22}O_4Na$ ($[M+Na]^+$): 277.1416, found 277.1410.

υ_{max} (ATR/cm^{-1}): 2975 (C-H stretch), 2912 (C-H stretch), 1723 (C=O stretch), 1177 (C-O stretch).

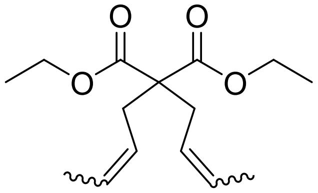

(13i) Diethyl 2,2-di(but-2-en-1-yl)malonate[10]

Prepared following the general procedure from diethyl malonate (0.76 mL, 5.00 mmol) and crotyl bromide (1.24 mL, 12.00 mmol), followed by purification by silica gel flash chromatography in Hex/EtOAc (5%) to yield the product as a colorless oil (0.890 g, 67%).

Rf = 0.60 (10% EtOAc/Hexane (v/v)).

^{1}H NMR (400 MHz, $CDCl_3$): δ 5.58-5.43 (m, 2H), 5.32-5.17 (m, 2H), 4.16 (q, J = 7.1 Hz, 4H), 2.66-2.50 (m, 4H), 1.67-1.60 (m, 6H), 1.29-1.19 (m, 6H).

HRMS: (ESI^+) m/z calcd. for $C_{15}H_{24}O_4Na$ ($[M+Na]^+$): 291.1572, found 291.1570.

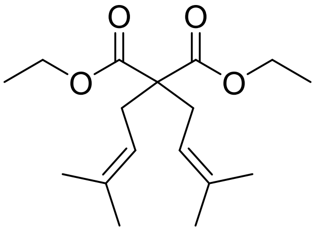

(13j) Diethyl 2,2-bis(3-methylbut-2-en-1-yl)malonate[11]

Prepared following the general procedure from diethyl malonate (0.61 mL, 4.00 mmol) and prenyl bromide (1.11 mL, 9.60 mmol), followed by purification by silica gel flash chromatography in Hex/EtOAc (5%) to yield the product as a colorless oil (0.581 g, 49%).

Rf = 0.61 (10% EtOAc/Hexane (v/v)).

^{1}H NMR (400 MHz, $CDCl_3$): δ 5.00-4.91 (m, 2H), 4.15 (q, J = 7.1 Hz, 4H), 2.60-2.54 (m, 4H), 1.67 (q, J = 1.4 Hz, 4H), 1.58 (d, J = 1.4 Hz, 6H), 1.22 (t, J = 7.1 Hz, 6H).

HRMS: (ESI^+) m/z calcd. for $C_{17}H_{28}O_4Na$ ($[M+Na]^+$): 319.1885, found319.1879.

υ_{max} (ATR/cm^{-1}): 2972 (C-H stretch, 2912 (C-H stretch), 1727 (C=O stretch), 1174 (C-O stretch).

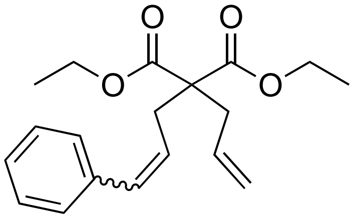

(13k) Diethyl 2-allyl-2-(3-phenylallyl)malonate[9]

Prepared following the general procedure from diethyl allylmalonate (0.99 mL, 5.00 mmol) and cinnamyl bromide (1.28 g, 6.00 mmol), followed by purification by silica gel flash chromatography in Hex/EtOAc (5%) to yield the product as a pale yellow oil (0.305 g, 20%).

Rf = 0.49 (10% EtOAc/Hexane (v/v)).

^{1}H NMR (400 MHz, $CDCl_3$): δ 7.33-7.18 (m, 5H), 6.47-6.42 (m, 1H), 6.09-6.00 (m, 1H), 5.77-5.65 (m, 1H), 5.17-5.09 (m, 2H), 4.19 (q, J = 7.1 Hz), 2.81-2.77 (m, 2H), 2.71-2.67 (m, 2H), 1.25 (t, J = 7.1 Hz, 6H).

HRMS: (ESI^+) m/z calcd. for $C_{19}H_{24}O_4Na$ ($[M+Na]^+$): 339.1572, found 229.156.

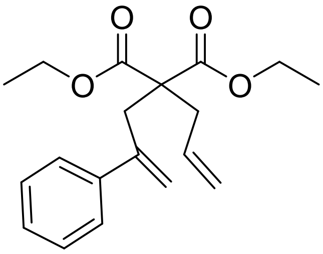

(13l) Diethyl 2-allyl-2-(2-phenylallyl)malonate[12]

Prepared following the general procedure from diethyl allylmalonate (0.99 mL, 5.00 mmol) and ((*E*)-4-Bromo-1-butenyl)benzene (0.86 mL, 6.00 mmol), followed by purification by silica gel flash chromatography in Hex/EtOAc (5%) to yield the product as a pale yellow oil (0.840 g, 53%).

Rf = 0.49 (10% EtOAc/Hexane (v/v)).

^{1}H NMR (400 MHz, $CDCl_3$): δ 7.37-7.23 (m, 5H), 5.63 (ddt, J = 17.5, 10.2, 7.3 Hz, 1H), 5.28 (d, J = 1.7 Hz, 1H), 5.18 (d, J = 1.7 Hz, 1H), 5.12-5.04 (m, 1H), 5.04-4.96 (m, 1H), 3.96 (dq,

J = 10.7, 7.2 Hz, 2H), 3.83 (dq, *J* = 10.8, 7.2 Hz, 2H), 3.18 (s, 2H), 2.60 (dt, *J* = 7.4, 1.3 Hz, 2H), 1.16 (t, *J* = 7.1 Hz, 6H).
HRMS: (ESI^+) m/z calcd. for $C_{19}H_{24}O_4Na$ ($[M+Na]^+$): 339.1572, found 339.1568.
υ_{max} (ATR/cm^{-1}): 2979 (C-H stretch), 1727 (C=O stretch), 1185 (C-O stretch).

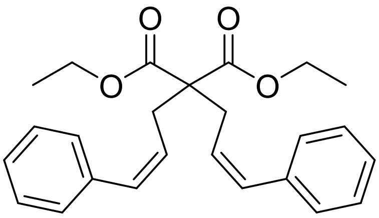

(13m) Diethyl 2,2-bis(3-phenylallyl)malonate[13]

Prepared following the general procedure from diethyl malonate (0.76 mL, 5.00 mmol) and cinnamyl bromide (2.56 g, 12.00 mmol), followed by purification by silica gel flash chromatography in Hex/EtOAc (5%) to yield the product as a colorless oil (0.900 g, 46%).
Rf = 0.61 (10% EtOAc/Hexane(v/v)).
^{1}H NMR (400 MHz, $CDCl_3$): δ 7.35-7.20 (m, 10H), 6.50-6.44 (m, 2H), 6.14-6.06 (m, 2H), 4.22 (q, *J* = 7.1 Hz, 4H), 2.85 (dd, *J* = 7.5, 1.1 Hz, 4H), 1.26 (t, *J* = 7.1 Hz, 6H).
HRMS: (ESI^+) m/z calcd. for $C_{25}H_{28}O_4Na$ ($[M+Na]^+$): 415.1885, found 415.1882.

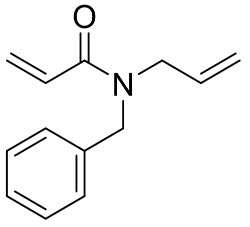

(14) *N*-allyl-*N*-benzylacrylamide[6]

To a solution of *N*-allylbenzylamine (0.78 mL, 5.00 mmol) in toluene (10 mL) at 0 ºC, triethylamine (0.84 mL, 6.00 mmol) and acryloyl chloride (0.49 mL, 6.00 mmol) was added. The solution was stirred for 3 h at 0 ºC, then filtered to remove precipitate which was washed with ice-cold Et_2O and then concentrated *in vacuo*. The product was purified by silica gel flash chromatography in Hex/EtOAc (10-30%) to yield the product as a colorless oil (0.430 g, 43%).
Rf = 0.29 (20% EtOAc/Hexane (v/v)).
^{1}H NMR (400 MHz, $CDCl_3$): δ 7.39-7.16 (m, 5H), 6.55 (dd, *J* = 16.7, 10.2 Hz, 1H), 6.43 (dd, *J* = 16.7, 2.2 Hz, 1H), 5.87-5.66 (m, 1H), 5.25-5.10 (m, 2H), 4.65 (s, 1H), 4.58 (s, 1H), 4.06 (d, *J* = 5.9 Hz, 1H), 3.89 (dd, *J* = 8.0, 5.3 Hz, 1H).
HRMS: (ESI^+) m/z calcd. for $C_{13}H_{15}NONa$ ($[M+Na]^+$): 224.1046, found 224.1050.

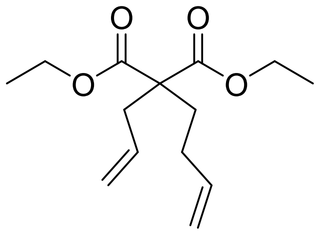

(15) Diethyl 2-allyl-2-(but-3-en-1-yl)malonate[7]

Sodium metal (0.117 g, 5.07 mmol) was added to dry EtOH (5 mL). Once dissolution was complete, diethyl allylmalonate (1.00 mL, 5.07 mmol) was added dropwise and the solution stirred for 10 min. 5-bromo-1-butene (0.72 mL, 6.08 mmol) was then added dropwise, following which the solution was brought to reflux and stirred for 3 h. The reaction was then cooled, acidified with DOWEX 50wx8 H^+ resin, filtered and organics concentrated *in vacuo*. The product was purified by silica gel flash chromatography in Hex/EtOAc (0-2%) to yield the product as a colorless oil (0.532 g, 41%).

Rf = 0.24 (5% EtOAc/Hexane (v/v))

^{1}H NMR (400 MHz, $CDCl_3$): δ 5.82-5.71 (m, 1H), 5.70-5.58 (m, 1H), 5.12-4.93 (m, 4H), 4.18 (q, J = 7.1 Hz, 4H), 2.64 (d, J = 7.4 Hz, 2h), 2.05 (q, J = 7.2 Hz, 2H), 1.24 (t, J = 7.1 Hz, 6H).

^{13}C{^{1}H} NMR (151 MHz, $CDCl_3$): δ 171.4, 138.3, 132.7, 118.9, 115.1, 61.3, 57.5, 37.1, 31.8, 23.4, 14.3).

HRMS: (ESI^+) m/z calcd. for $C_{14}H_{22}O_4Na$ ($[M+Na]^+$): 227.1410, found 227.1409.

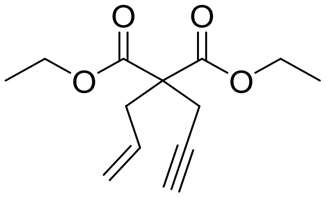

(16) Diethyl 2-allyl-2-(prop-2-yn-1-yl)malonate[8]

Diethyl allylmalonate (1.00 g, 5.00 mmol) was added to dry Et_2O (10 mL) and cooled to 0 ºC. Sodium metal (0.115 g, 5.00 mmol) was dissolved in dry EtOH (5mL), and then added dropwise to the malonate solution and stirred for 10 min at rt. Propargyl bromide (80% in toluene, 0.65 mL, 6.00 mmol) was then added dropwise, and the reaction mix stirred for a further 1 h. Brine was then added, and the mix extracted with Et_2O (x3). The combined organics were dried over $MgSO_4$, filtered, and concentrated *in vacuo* to yield the product as a colorless oil (1.096 g, 92%).

^{1}H NMR (400 MHz, $CDCl_3$): δ 5.69=5.57 (m, 1H), 5.22-5.10 (m, 2H), 4.24-4.17 (m, 4H), 2.83-2.77 (m, 4H), 2.01 (s, 1H), 1.25 (t, J = 7.5 Hz, 6H).

HRMS: (ESI^+) m/z calcd. for $C_{13}H_{19}O_4$ ($[M+H]^+$): 239.1283, found 239.1280.

Trityl Thioester Synthesis

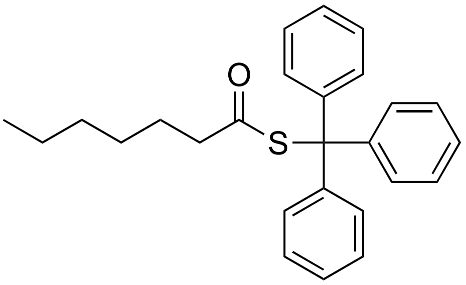

(17a) *S*-trityl heptanethioate[14]

Prepared following the general procedure from heptanoic acid (0.3 mL, 2.30 mmol) and purified by silica gel flash chromatography in Hex/EtOAc (5%) to yield the product as a yellow oil (1.590 g, 81%).

Rf = 0.42 (20% EtOAc/Hexane (v/v)).

^{1}H NMR (400 MHz, $CDCl_3$): δ 7.35-7.25 (m, 15H), 2.53 (t, *J* = 7.3 Hz, 2H), 1.65-1.58 (m, 2H), 1.34-1.26 (6H, m), 0.91 (t, J = 7.0 Hz, 3H).

HRMS: (ESI^+) m/z calcd. for $C_{26}H_{28}OSNa$ ($[M+Na]^+$): 411.1740, found 411.1753.

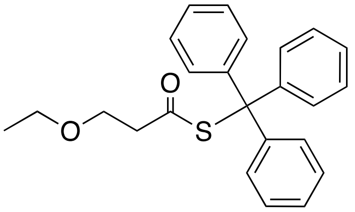

(17b) *S*-trityl 3-ethoxypropanethioate

Prepared following the general procedure from 3-ethoxypropanoic acid (0.3 mL, 2.50 mmol) and purified by silica gel flash chromatography in Hex/EtOAc (5-10%) to yield the product as a white solid (0.622 g, 83%).

R_f = 0.42 (10% Hex/EtOAc (v/v)).

^{1}H NMR (400 MHz, $CDCl_3$): δ 7.24-7.31 (m, 15H), 3.66 (t, *J* = 6.2 Hz, 2H), 3.48 (q, *J* = 7.0 Hz, 2H), 2.77 (t, *J* = 6.3 Hz, 2H), 1.22 (t, *J* = 7.0, 3H).

$^{13}C\{^1H\}$ NMR (151 MHz, $CDCl_3$): δ 195.2, 143.7, 129.8, 127.7, 127.1, 70.4, 66.4, 66.1 44.1, 15.1.

HRMS: (ESI) m/z calcd. for $C_{24}H_{24}NaO_2S$ ($[M+Na]^+$): 399.1389, found 399.1393.

υ_{max} (ATR/cm^{-1}): 2980 (C-H stretch), 1731 (C=O), 1448 (Ar C-C stretch), 1256 (C-O stretch).

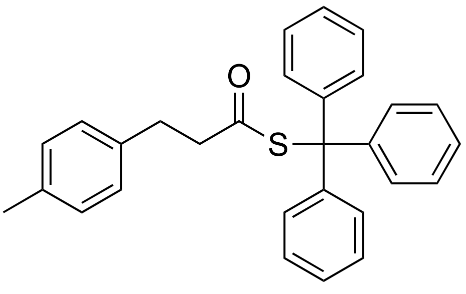

(17c) *S*-trityl 2-(*p*-tolyl)ethanethioate[14]

Prepared following the general procedure from 3-(*p*-tolyl)propanoic acid (0.290 g, 1.77 mmol) and purified by silica gel flash chromatography in Hex/EtOAc (2%) to yield the product as a yellow oil (0.742 g, 99%).

Rf = 0.41 (5% EtOAc/Hexane (v/v)).

^{1}H NMR (400 MHz, $CDCl_3$): δ 7.33-7.24 (m, 15H), 7.12 (dd, J = 27.9 Hz, J = 7.9 Hz, 4H), 2.88 (m, 4H), 2.39 (s, 3H).
HRMS: (ESI^+) m/z calcd. for $C_{29}H_{26}OSNa$ ($[M+Na]^+$): 445.1704, found 445.1724.

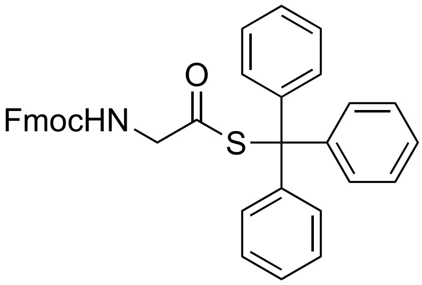

(17d) *S*-trityl 2-((((9*H*-fluoren-9-yl)methoxy)carbonyl)amino)ethanethioate

Prepared following the general procedure from Fmoc-Gly-OH (1.400 g, 4.17 mmol) and purified by silica gel flash chromatography in Hex/EtOAc (10%) to yield the product as white crystals (2.300 g, 88%).
M.P. = 65-70 ºC
^{1}H NMR (400 MHz, $CDCl_3$): δ 7.74 (d, J = 7.6 Hz, 2H), 7.55 (d, J = 7.6 Hz, 2H), 7.37 (t, J = 7.6 Hz, 2H), 7.33-7.25 (m, 17H), 5.30 (t, J = 5.8 Hz, 1H), 4.42 (d, J = 6.8 Hz, 2H), 4.24 (t, J = 6.8 Hz, 1H), 4.15 (d, J = 5.6 Hz, 2H)
HRMS: (ESI^+) m/z calcd. for $C_{29}H_{26}OSNa$ ($[M+Na]^+$): 578.1766, found 578.1760.

Thioacid-Mediated Cyclisation of 1,6-Dienes

Cyclisation products are obtained as a mixture of diastereomers, exemplified by tocsy analysis of *S*-((1-acetyl-4-methylpyrrolidin-3-yl)methyl) ethanethioate **2f** (Fig. S2).

Figure S2: NMR tocsy analysis of diastereomers of compound **2f**.

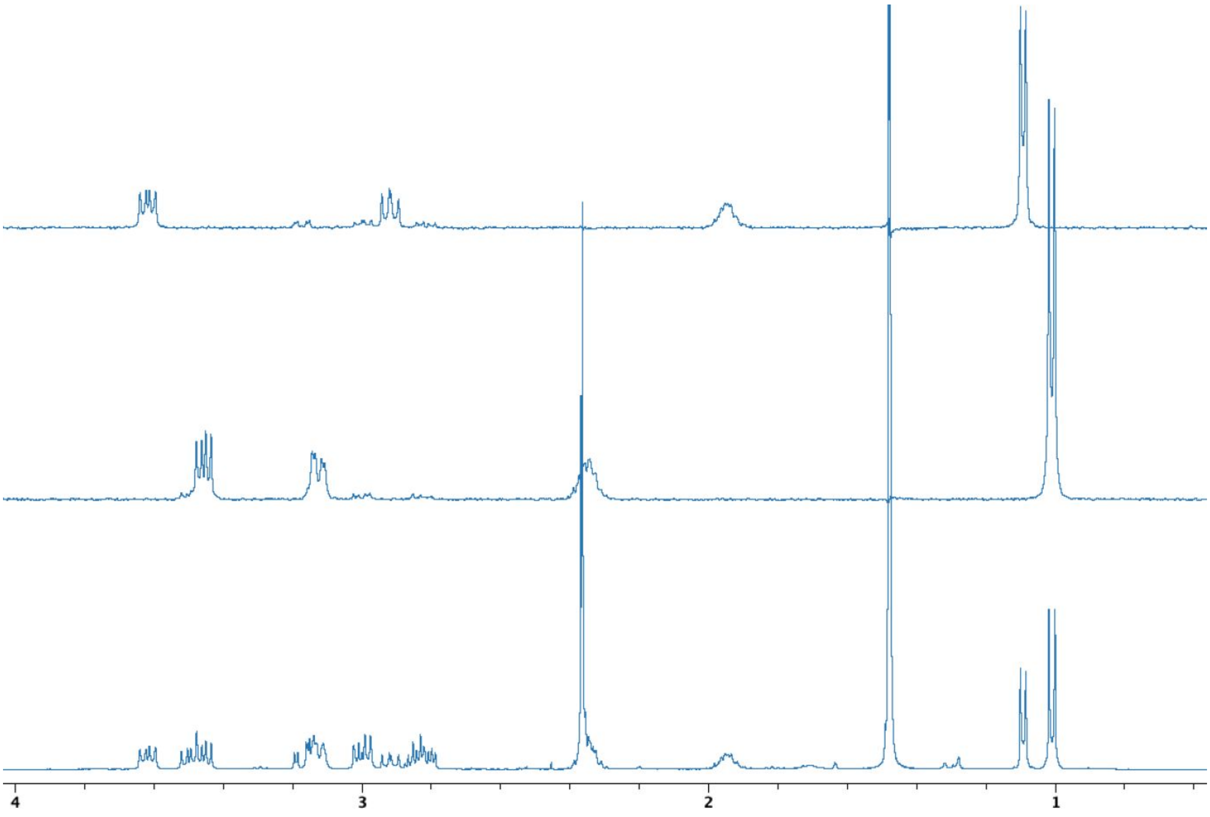

Gram-Scale Example

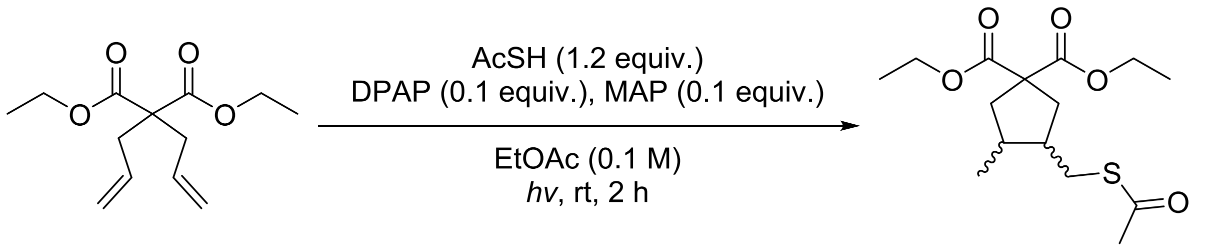

Prepared following the general procedure from diethyl diallylmalonate (1.209 mL, 1.2015 g, 5.0 mmol) and purified by silica gel flash chromatography in Hex/EtOAc (0-25%) to yield the product as a colorless oil (rotamers of diastereomers, 1.4314 g, 90%).

Derivatization

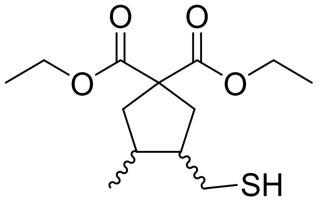

(8) Diethyl 3-(mercaptomethyl)-4-methylcyclopentane-1,1-dicarboxylate

To a solution of **2a** (0.0949 g, 0.30 mmol) in anhydrous ethanol (3.0 mL), a catalytic amount of sodium (21 wt % in ethanol) was added. The mix was stirred under Ar for 30 min, then quenched using freshly washed Amberlite H^+ resin, filtered and concentrated *in vacuo* to yield the desired product as a pale yellow oil (0.0823 g, >99%).

Rf = 0.59 (20% Hex/EtOAc (v/v)).

^{1}H NMR (400 MHz, $CDCl_3$): δ 4.17 (q, J = 7.1 Hz, 4H), 2.62 – 2.50 (m, 1H), 2.49 – 2.37 (m, 2H), 2.35 – 2.21 (m, 1H), 2.19 – 2.07 (m, 2H), 2.03 – 1.92 (m, 1H), 1.24 (t, J = 7.1, 6H), 0.89 (dd, J = 7.1, 2.6 Hz, 3H).

^{13}C{^{1}H} NMR (151 MHz, $CDCl_3$): δ 172.9, 61.6, 58.9, 46.7, 41.2, 37.9, 35.8, 24.9, 14.5, 14.0.

HRMS: (APCI) m/z calcd. for $C_{13}H_{23}O_4S$ ([M+H]$^+$): 275.1317, found 275.1310.

υ_{max} (ATR/cm^{-1}): 2979 (C-H stretch), 1725 (C=O stretch), 1679 (C=O stretch).

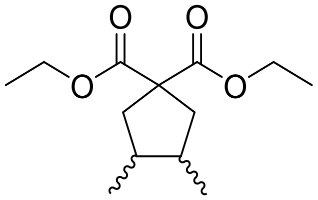

(9) Diethyl 3,4-dimethylcyclopentane-1,1-dicarboxylate[17]

To a solution of **8** (0.4340 g, 1.58 mmol) and TCEP (1.1860 g, 4.741 mmol) in DMF, was added DPAP (0.0400 g, 0.158 mmol) and MAP (0.0240 g, 0.158 mmol). The resulting mixture was irradiated under Ar, following which it was concentrated *in vacuo* and resuspended in EtOAc. The organics were washed with HCl, sat. aq. $NaHCO_3$ and brine, then dried over $MgSO_4$, filtered, and concentrated *in vacuo*. The product was purified by silica gel flash chromatography in hex/EtOAc (1-5%) to yield the product as a colorless oil (0.2450 g, 66%).

Rf = 0.53 (5% Hex/EtOAc (v/v)).

^{1}H NMR (400 MHz, $CDCl_3$): δ 4.20 – 4.13 (m, 4H), 2.38 (dd, J = 13.6, 7.0 Hz, 2H), 2.10 (dq, J = 11.7, 6.8 Hz, 2H), 1.94 (dd, J = 13.6, 7.0 Hz, 2H), 1.27 – 1.20 (m, 6H), 0.89 – 0.84 (m, 6H).

HRMS: (ESI$^+$) m/z calcd. for $C_{13}H_{22}O_4Na$ ([M+Na]$^+$): 265.1409, found 265.1410.

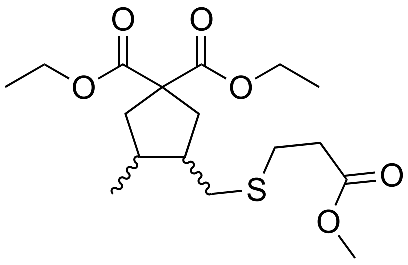

(10a) Diethyl 3-(((3-methoxy-3-oxopropyl)thio)methyl)-4-methylcyclopentane-1,1-dicarboxylate

Thiol **8** (0.2820 g, 1.02 mmol) was dissolved in anhydrous DCM (10 mL) and NEt_3 (0.28 mL, 2.03 mmol) was added. The solution was stirred for 5 min, following which methyl acrylate (0.14 mL, 1.53 mmol) was added dropwise, and then stirred for a further 16 h. Upon completion of reaction, the organic phase was washed with aq. 1 M HCl, sat. aq. $NaHCO_3$ and brine, then dried over $MgSO_4$, filtered, and concentrated *in vacuo* to yield the product as a pale yellow oil (0.2611 g, 71%).

Rf = 0.55 (20% Hex/EtOAc (v/v)).

^{1}H NMR (400 MHz, $CDCl_3$): δ 4.17 (q, J = 7.1, 4H), 3.69 (s, 3H), 2.81 – 2.72 (m, 2H), 2.63 – 2.54 (m, 2H), 2.50 – 2.38 (m, 2H), 2.29 – 1.95 (m, 3H), 1.23 (t, J = 7.1, 6H), 0.89 (dd, J = 7.0, 3.2 Hz, 3H).

^{13}C{^{1}H} NMR (151 MHz, $CDCl_3$): δ 172.9, 172.8, 61.6, 59.0, 51.9, 42.3, 41.4, 38.3, 35.9, 34.9, 33.0, 27.5, 14.8, 14.2.

HRMS: (ESI^+) m/z calcd. for $C_{17}H_{28}O_6SNa$ ($[M+Na]^+$): 383.1504, found 383.1509.

υ_{max} (ATR/cm^{-1}): 2957 (C-H stretch), 1724 (C=O stretch).

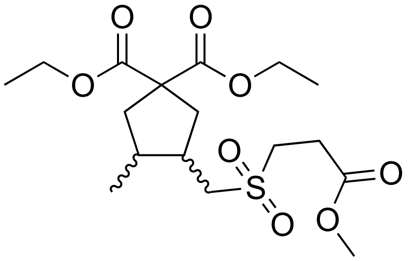

(11) Diethyl 3-(((3-methoxy-3-oxopropyl)sulfonyl)methyl)-4-methylcyclopentane-1,1-dicarboxylate

To a solution of **10a** (0.2110 g, 0.59 mmol) in EtOH (2 mL), a solution of Oxone (0.7200 g, 2.34 mmol) in H_2O (2 mL) was added, and the slurry was stirred for 16 h. The mix was then diluted with H_2O (10 mL) and extracted with DCM (x3). The organics were then washed with H_2O and brine, then dried over $MgSO_4$, filtered and concentrated *in vacuo* to yield the product as a pale yellow oil (0.2131 g, 92%).

Rf = 0.45 (20% Hex/EtOAc (v/v)).

^{1}H NMR (400 MHz, $CDCl_3$): δ 4.20 (q, J = 7.1 Hz, 4H), 3.77 (s, 3), 3.37 – 3.31 (m, 2H), 3.12 – 2.96 (m, 2H), 2.91 (t, J = 7.4 Hz, 2H), 2.73 – 2.22 (m, 4H), 2.02 (dd, J = 13.8, 5.2 Hz, 1H), 1.27 (t, J = 7.1 Hz, 6H), 0.93 (d, J = 7.1 Hz, 3H).

^{13}C{^{1}H} NMR (151 MHz, $CDCl_3$): δ 172.3, 171.6, 61.8, 58.7, 53.8, 52.6, 49.2, 41.1, 38.2, 36.6, 36.5, 26.9, 15.1, 14.1.

HRMS: (ESI^+) m/z calcd. for $C_{17}H_{28}O_8SNa$ ($[M+Na]^+$): 415.1403, found 415.1409.

υ_{max} (ATR/cm^{-1}): 2982 (C-H stretch), 1723 (C=O stretch), 1311 (S=O stretch), 1122 (S=O stretch).

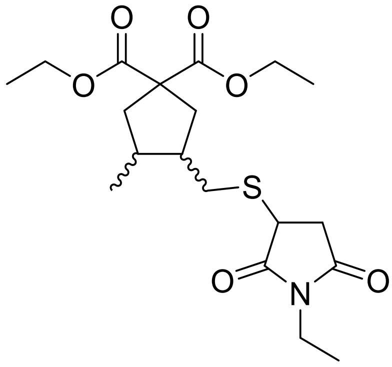

(10b) Diethyl 3-(((1-ethyl-2,5-dioxopyrrolidin-3-yl)thio)methyl)-4-methylcyclopentane-1,1-dicarboxylate

8 (0.0686 g, 0.25 mmol was dissolved in anhydrous DCM (2.5 mL) and NEt_3 (0.070 mL, 0.0506 g, 0.50 mmol) was added. The mix was stirred for 5 min under Ar, following which *N*-ethyl maleimide (0.0626 g, 0.50 mmol) was added and the mix was stirred for 16 h under Ar. The reaction was then diluted with DCM and washed with 0.2 M HCl_{aq}, and water, dried over $MgSO_4$, filtered, and concentrated *in vacuo*. The product was then purified by silica gel flash chromatography in Hex/EtOAc (0-25%) to yield the product as a colorless oil (0.0950 g, 95%).
Rf = 0.29 (30% Hex/EtOAc (v/v)).

^{1}H NMR (400 MHz, $CDCl_3$): δ 4.20 (qd, *J* = 7.1, 1.1 Hz), 3.75-3.70 (m, 1H), 3.59 (q, *J* = 7.2 Hz, 2H), 3.13 (dd, *J* = 18.7, 9.1 Hz, 1H), 2.99-2.89 (m, 1H), 2.82-2.62 (m, 1H), 2.56-2.40 (m, 3H), 2.33-2.12 (m, 3H), 2.06-1.99 (m, 1H), 1.26 (t, *J* = 7.1 Hz, 6H), 1.20 (t, *J* = 7.2 Hz, 3H), 0.93 (t, *J* = 6.7 Hz).

^{13}C{^{1}H} NMR (151 MHz, $CDCl_3$): δ 176.5, 176.3, 174.6, 174.5, 172.7, 172.7, 172.6, 172.6, 61.5, 58.8, 58.7, 42.2, 41.6, 41.2, 41.1, 39.6, 38.7, 38.1, 38.1, 36.1, 36.0, 35.8, 34.0, 32.5, 32.2, 14.7, 14.6, 14.0, 12.9, 12.9.

HRMS: (ESI$^+$) m/z calcd. for $C_{19}H_{29}O_6SNa$ ([M+Na]$^+$): 422.1608, found 422.1610.

υ_{max} (ATR/cm^{-1}): 2978 (C-H stretch), 1724 (C=O stretch), 1698 (C=O stretch).

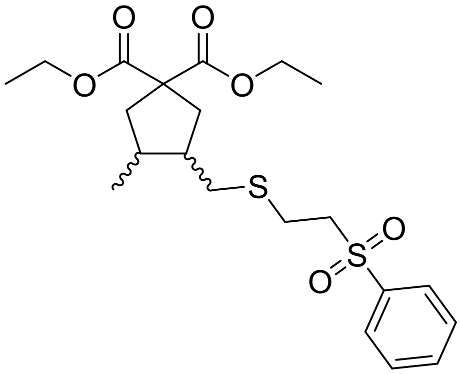

(10c) Diethyl 3-methyl-4-(((2-(phenylsulfonyl)ethyl)thio)methyl)cyclopentane-1,1-dicarboxylate

8 (0.0686 g, 0.25 mmol was dissolved in anhydrous DCM (2.5 mL) and NEt_3 (0.070 mL, 0.0506 g, 0.50 mmol) was added. The mix was stirred for 5 min under Ar, following which phenyl vinyl sulfone (0.0841 g, 0.50 mmol) was added and the mix was stirred for 16 h under Ar. The reaction was then diluted with DCM and washed with 0.2 M HCl_{aq}, and water, dried over $MgSO_4$, filtered, and concentrated *in vacuo*. The product was then purified by silica gel flash chromatography in Hex/EtOAc (10-25%) to yield the product as a pale yellow oil (0.0728 g, 66%).

Rf = 0.11 (15% Hex/EtOAc (v/v)).

^{1}H NMR (400 MHz, $CDCl_3$): δ 7.95-7.92 (m, 2H), 7.72-7.67 (m, 1H), 7.63-7.58 (m, 2H), 4.18 (qd, J = 7.1, 3.1 Hz, 4H), 3.37-3.25 (m, 2H), 2.83-2.78 (m, 2H), 2.58-2.53 (m, 1H), 2.46-2.35 (m, 3H), 2.27-2.05 (m, 3H), 2.01-1.95 (m, 1H), 1.25 (td, J = 7.11, 2.05 Hz, 6H), 0.87 (d, J = 0.87, 7.04 Hz).

^{13}C{^{1}H} NMR (151 MHz, $CDCl_3$): δ 172.6, 138.7, 134.0, 129.4, 128.1, 61.5, 58.7, 56.4, 46.2, 42.5, 42.1, 41.1, 39.6, 38.1, 36.1, 35.9, 33.1, 24.8, 14.6, 14.0.

HRMS: (ESI$^+$) m/z calcd. for $C_{21}H_{30}O_6S_2Na$ ([M+Na]$^+$): 465.1376, found 465.1378.

υ_{max} (ATR/cm^{-1}): 2962 (C-H stretch), 1723 (C=O stretch), 1145 (S=O stretch).

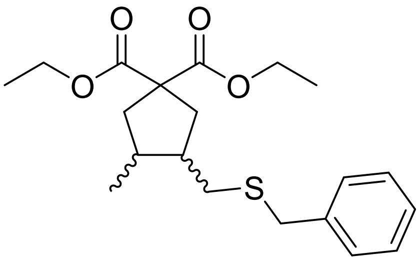

(12) Diethyl 3-((benzylthio)methyl)-4-methylcyclopentane-1,1-dicarboxylate

8 (0.0686 g, 0.25 mmol) was dissolved in anhydrous ethanol and NEt_3 (0.070 mL, 0.0506 g, 0.50 mmol) was added, followed by benzyl bromide (0.059 mL, 0.0855 g, 0.50 mmol). The reaction was then diluted with DCM and washed with 0.2 M HCl_{aq}, and water, dried over $MgSO_4$, filtered, and concentrated *in vacuo*. The product was then purified by silica gel flash chromatography in Hex/EtOAc (0-10%) to yield the product as a colorless oil (0.0699 g, 77%).
Rf = 0.27 (10% Hex/EtOAc (v/v)).
^{1}H NMR (400 MHz, $CDCl_3$): δ 7.35-7.31 (m, 4H), 7.28-7.23 (m, 1H), 4.19 (qd, J = 7.1, 3.8 Hz, 4H), 3.72 (s, 2H), 2.68-2.39 (m, 3H), 2.39-2.15 (m, 3H), 2.14-1.97 (2H), 1.27 (td, J = 7.1, 3.8 Hz, 6H), 0.84 (d, J = 6.8, 3H).
^{13}C{^{1}H} NMR (151 MHz, $CDCl_3$): δ 172.8, 172.7, 138.5, 128.8, 128.5, 126.9, 61.4, 61.4, 58.8, 41.9, 41.3, 38.2, 36.6, 35.6, 32.0, 13.6, 14.0.
HRMS: (ESI$^+$) m/z calcd. for $C_{20}H_{28}O_4SNa$ ([M+Na]$^+$): 387.1601, found 387.1606.
υ_{max} (ATR/cm^{-1}): 2961 (C-H stretch), 1725 (C=O stretch).

Computational Work

All calculations reported in the manuscript were carried out using standard computational methods as implemented in Gaussian 16.[18] All initial explorations of minima and transition structures were performed at the uMP2[19]/def2svp[20] level of theory in a solvent model SMD[21] (ethylethanoate) at 298 K in order to mimic experimental conditions. Single point energies for lowest energy small basis set calculations were computed using uMP2/ def2tzvp (ethylethanoate). The free energies reported in the document were obtained by adding the free energy correction from the small bases set calculations to the potential energy obtained from the high-level single-point energy calculations. To assess the connectivity between each transition states and the minima to which it evolves, intrinsic reaction coordinates (IRC) calculations have been performed.

Figure S3: PES for cyclisation to yield compound 2b.

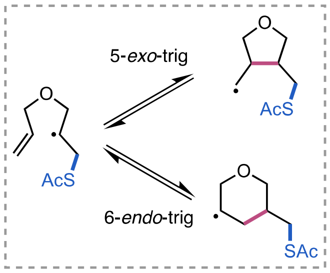

Molecule	ΔG (kcal/mol)
TS_*exo*_a	0.0
TS_*exo*_b	0.3
TS_*endo*_a	2.9
TS_*endo*_b	5.4

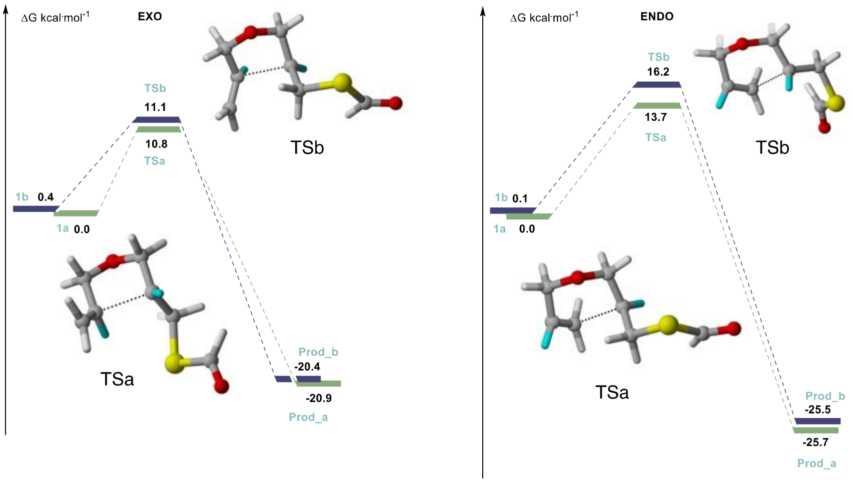

Figure S4: Geometrical arrangements in cyclization reactions.

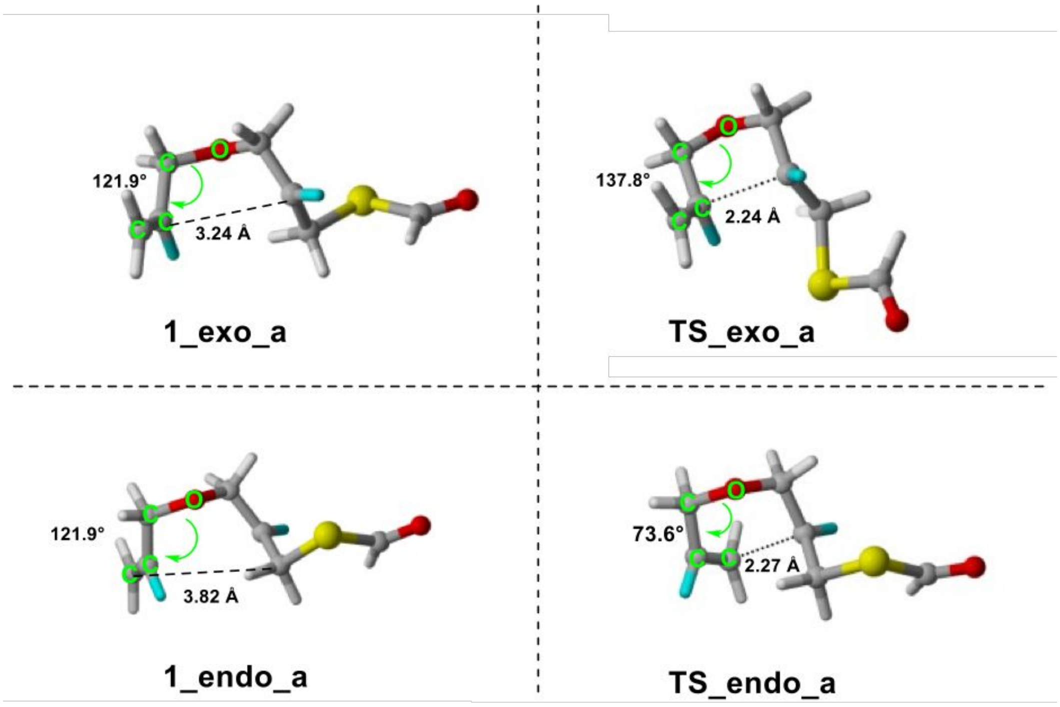

Geometrical penalties : in order to promote the cyclisation, the dihedral angle, O-C-C-C (Figure in green) changes from 121.9 to 73.6 for the TS_endo_a case (from 123.6 to 74.5 for the TS_endo_b) and therefore, a rotation has been forced to facilitate the encounter, making the energetic barrier higher than the exo conformer, since the pre-Ts assembly for the 5-exo conformer is almost ready for the cyclisation, in terms of geometry.
Thus, the overall process is not thermodynamically favorable, in complete agreement with the experimental results.

Table SI1: Cartesian coordinates of all the compounds at the uMP2/def2tzvp//uMP2/def2svp computational level.

1_endo_a

O 0 -2.6419114684 -0.7134818659 0.1343242918
C 0 -2.7118997687 0.702396005 0.1615480353
C 0 -1.689664902 1.375348931 -0.7060966875
H 0 -2.619110718 1.0765755087 1.1994783395
H 0 -3.7242878401 0.9505115338 -0.1937345025
H 0 -1.6934952826 1.0917365768 -1.7648965448
C 0 -1.4404463248 -1.2343574117 0.6653455538
C 0 -0.4425695088 -1.5923525743 -0.3867885129
H 0 -1.7103012626 -2.1378830919 1.2484833616
H 0 -0.9935141657 -0.5291145364 1.3945355018
S 0 2.149657897 -0.5974288545 -0.6206622971
C 0 -0.8164566541 2.2669138618 -0.2592158845
H 0 -0.0968391084 2.7488656209 -0.9249391171
C 0 1.8680700365 0.6056985869 0.6438868555
O 0 2.4087712963 1.6872243183 0.6781950108
H 0 1.1581816537 0.2502604812 1.4265702994
C 0 0.9657646328 -1.8972775429 -0.0524917572
H 0 1.3242406878 -2.809656776 -0.55452517
H 0 1.1168441627 -2.0389128552 1.0290355806
H 0 -0.7770532529 -1.6503989038 -1.4243468855
H 0 -0.7916851096 2.5598039885 0.7949245291

1_endo_b

O 0 0.796860759 1.0772526551 0.2124279511
C 0 2.1723375562 1.2627042298 -0.0807456031
C 0 2.8113145686 0.089756655 -0.7648521134
H 0 2.7345968945 1.5042346283 0.841237911
H 0 2.2133635221 2.1444156786 -0.7392405463
H 0 2.3651490893 -0.2172816444 -1.718017932
C 0 0.5441806606 0.1042657988 1.209185646
C 0 0.2160942068 -1.2441732717 0.6539255768
H 0 -0.3182444217 0.4782087532 1.7963001574
H 0 1.3924167885 0.0266936633 1.9144244636
S 0 -2.3298499714 -0.9106020796 -0.3913313377
C 0 3.8575214198 -0.5600547409 -0.2824779584
H 0 4.3152472455 -0.2707433891 0.6686187869
C 0 -2.88557167 -2.4180270493 0.348036963
O 0 -4.0153727503 -2.6041311458 0.7373968244
H 0 -2.0850361104 -3.1940010273 0.4038783185
H 0 0.1980541669 -2.0835449066 1.356040878
C 0 -0.5462607264 -1.3560590593 -0.6072329218
H 0 -0.5074577131 -2.3666765267 -1.0384346473
H 0 -0.1840994535 -0.6400740625 -1.357103592
H 0 4.3082280293 -1.4002638888 -0.8165963747

1_exo_b

O 0 -1.41875326 -0.7892603168 -0.8615624024
C 0 -2.568086832 -0.3649688869 -0.143910158
C 0 -2.3724226701 0.8895294953 0.6583468896
H 0 -2.9097035362 -1.1812486796 0.5220514478
H 0 -3.3532331005 -0.1986311282 -0.8971227729
H 0 -1.6280104357 0.8559442748 1.4618924075
C 0 -0.4451103649 -1.4473586867 -0.0765068407
C 0 0.5005095096 -0.5193767808 0.616452585
H 0 0.1208066454 -2.0921288407 -0.7771431086
H 0 -0.9218894925 -2.1226234837 0.6591833886
H 0 1.1062544428 -0.9287918883 1.4307662539
S 0 2.2169173174 0.2962609678 -1.3957707795
C 0 -3.0740554055 2.0121798418 0.448589826
H 0 -3.8225139663 2.0716274118 -0.3475656817
H 0 -2.927195904 2.9017771557 1.0660279614
C 0 3.6281842982 0.0672650755 -0.3535123695
O 0 4.7163292202 -0.2753685465 -0.7544908091
H 0 3.4152920772 0.2904216808 0.7195418413
C 0 0.9757068898 0.6937309163 -0.0842375343
H 0 1.435149531 1.4257489213 0.5956498499
H 0 0.1585393161 1.1884278871 -0.6266414445

1_exo_a

O 0 1.6295687582 -1.2484025442 -0.0997064931
C 0 2.9629252797 -0.781955644 -0.2294804837
C 0 3.0661287418 0.6728491874 -0.5830414112
H 0 3.4078788031 -1.3915772821 -1.0314112071
H 0 3.5327719426 -0.9781359595 0.6985920958
H 0 2.57412932 0.9816268883 -1.5128013151
C 0 0.9394487864 -0.7227732875 1.0189874427
C 0 0.089505026 0.4620295603 0.6901976352
H 0 1.6398088259 -0.4705341455 1.8367655612
H 0 0.2957068963 -1.5410063386 1.3989865174
H 0 -0.318528231 1.0335215874 1.5296885215
S 0 -2.0174983631 -0.6312350851 -0.7419761807
C 0 3.7096088084 1.5628846686 0.1539944419
H 0 4.2044848482 1.2749648759 1.0867320029
H 0 3.7792397905 2.6122946335 -0.143088612
C 0 -3.1987224255 0.3019930225 0.1862280703
O 0 -4.3152400967 -0.0852438865 0.4434079468
H 0 -2.8120321174 1.3013462391 0.4990627345
C 0 -0.5898616001 0.5405852605 -0.6199664361
H 0 0.0776235991 0.2294761314 -1.4346895537
H 0 -0.9691375925 1.5484521181 -0.8416642775

prod_endo_a

O 0 -2.0303110404 -1.1331995281 -0.834790598
C 0 -2.8832043961 -0.019229349 -0.6258999388
C 0 -2.1223404754 1.2410731229 -0.3806014057
H 0 -3.5573836832 -0.2283088138 0.2374694205
H 0 -3.5214627536 0.0655545068 -1.5177314122
H 0 -2.6333967048 2.194953197 -0.5358699717
C 0 -1.1144842905 -1.296408976 0.2268529884
C 0 -0.1482877935 -0.1189387436 0.320079494
H 0 -0.5733055008 -2.2338846925 0.0348448507
H 0 -1.65159527 -1.4029015647 1.1935943676
S 0 2.0217164259 -1.7029483759 1.1726169802
C 0 -0.9492616186 1.1705933604 0.5417617249
H 0 -0.3011728882 2.0553159608 0.4330938702
C 0 3.1579906919 -0.9312694288 0.0542393903
O 0 4.0897901238 -1.502540361 -0.4615505038
H 0 2.9331107311 0.1497263084 -0.1103466605
C 0 0.8862024291 -0.3001170185 1.4216690436
H 0 0.3936352776 -0.482415967 2.3903590741
H 0 1.49215846 0.6126189431 1.5383729259
H 0 0.368672869 -0.046724525 -0.6531594751
H 0 -1.3067792431 1.1736898446 1.5943096653

prod_endo_b
O 0 -1.203390829 -1.1645948699 -1.3274375779
C 0 -2.0856354159 -0.0746877934 -1.1059160863
C 0 -1.3643056937 1.2021872893 -0.8241672227
H 0 -2.7624367593 -0.3194785758 -0.2540562208
H 0 -2.7172653939 0.0116821161 -2.0024590137
H 0 -1.911958467 2.14025933 -0.9490561878
C 0 -0.2849530972 -1.313081534 -0.2628278416
C 0 0.6536598938 -0.1117988834 -0.1738177206
H 0 0.2736443609 -2.2405653316 -0.4534226309
H 0 -0.8217612986 -1.4304417428 0.7001133947
S 0 2.5920134558 -1.3093373579 -1.8302363738
C 0 -0.1883437814 1.1451112407 0.0969324726
H 0 -0.5440483408 1.1016397324 1.1475885964
C 0 3.9186413425 -0.8570255385 -0.7496676935
O 0 4.9181280633 -1.5186919494 -0.5909560412
H 0 3.7445179556 0.1206485637 -0.2390809796
H 0 1.3502709072 -0.2756211479 0.6664055527
C 0 1.4565913465 0.0637746933 -1.4601089926
H 0 2.0447306483 0.9946807258 -1.4326399739
H 0 0.7806119707 0.128823174 -2.3248148224
H 0 0.4260207224 2.0564145693 0.0170862227

prod_exo_a
O 0 -2.1592605432 -1.1428337083 -0.1223210019
C 0 -1.9128663954 -0.3583770801 1.0442884365
C 0 -0.900403359 0.7177311707 0.6418901353

H 0 -1.5129621654 -1.0026438096 1.8471712166
H 0 -2.8590152887 0.0780592279 1.4026425926
H 0 -0.2303703154 0.9734644166 1.479080828
C 0 -1.2559697784 -0.7646861138 -1.1554073997
C 0 -0.1120268172 -0.0309898223 -0.4602069
H 0 -1.7524432476 -0.1013881018 -1.8868778612
H 0 -0.924124063 -1.6712270504 -1.6865511218
H 0 0.3926238357 0.672439054 -1.1416021605
S 0 2.2307851266 -0.2687799671 1.0844260986
C 0 -1.5490385077 1.9544134227 0.1187010409
H 0 -2.5662068338 1.9265465566 -0.2769035845
H 0 -0.9498241305 2.8486079471 -0.0626026807
C 0 3.2691821454 0.2828301654 -0.2402865647
O 0 4.2787738089 0.9283591039 -0.0833762133
H 0 2.9016370282 -0.047826171 -1.241112895
C 0 0.904407211 -1.0244793801 0.0950570961
H 0 1.3722710796 -1.5920752049 -0.7251226176
H 0 0.4193159099 -1.7612128656 0.7529632062

prod_exo_b
O 0 -2.1406923831 -0.916147562 -0.2390103654
C 0 -1.9841130541 0.3900653006 -0.7855788141
C 0 -0.7819549361 1.0385856036 -0.079910582
H 0 -2.9038194243 0.9812748383 -0.6405568586
H 0 -1.8058636163 0.3052083148 -1.8722139518
H 0 -1.1381884186 1.5282270264 0.8426257331
C 0 -1.1705448182 -1.1055427618 0.7810921131
C 0 0.0043750438 -0.2152711964 0.3915764695
H 0 -0.9165327441 -2.1755503021 0.8269058801
H 0 -1.5646578659 -0.800568312 1.7696313121
H 0 0.6409408402 0.0142785616 1.260955513
S 0 2.3865286791 0.022281295 -1.0956565229
C 0 -0.0645372788 2.0383642106 -0.9116278916
H 0 0.012456654 1.9076855876 -1.9926145565
H 0 0.5548417818 2.8051372734 -0.4453406291
C 0 3.4362027733 -0.6981244043 0.1337253379
O 0 4.5898827097 -0.3801524986 0.305972888
H 0 2.9226484869 -1.4894816342 0.7311156469
C 0 0.8450552586 -0.8626935255 -0.7034815887
H 0 1.1196958634 -1.8904052538 -0.4167526791
H 0 0.285059919 -0.9407765213 -1.6474163139

TS_endo_a
O 0 -2.7412336616 -0.8391667652 -0.1972778417
C 0 -2.7587715427 0.5899942604 -0.1670194018
C 0 -1.5044211636 1.1190011158 -0.7881351815
H 0 -2.8518157508 0.9367825099 0.8799284377
H 0 -3.6621252136 0.891349145 -0.7143810562
H 0 -1.4823184649 1.2539628337 -1.873386154
C 0 -1.7256148238 -1.3909667636 0.6129559641

C 0 -0.3271383532 -1.2427224836 0.074847475
H 0 -1.9711035379 -2.4661083263 0.708348757
H 0 -1.7723743459 -0.9642218726 1.6368219279
S 0 2.4452301308 -1.357371032 0.3231272328
C 0 -0.3586097203 1.0369475147 -0.0928769743
H 0 0.6054572606 1.2611905768 -0.5562272696
C 0 2.7832042157 0.2093828584 1.075773935
O 0 3.7806162849 0.856135819 0.8577505049
H 0 1.9887897724 0.5270528675 1.7917516802
C 0 0.7776272041 -1.6691704115 0.9868023961
H 0 0.737999786 -2.7577207949 1.1829520325
H 0 0.6971799553 -1.1826486398 1.9744481957
H 0 -0.2060224105 -1.5051064616 -0.9818023158
H 0 -0.3768385009 1.0039872296 0.9999809661

TS_endo_b
O 0 -0.9598310482 -1.3621936924 0.211828516
C 0 -2.3391471026 -1.300844973 -0.1638570553
C 0 -2.6122334417 -0.017750824 -0.8858054506
H 0 -2.9702794891 -1.3799702244 0.7418206268
H 0 -2.5193199525 -2.183843831 -0.7918089964
H 0 -2.4603227977 0.0093043186 -1.9686830867
C 0 -0.6089032355 -0.3648133898 1.1525323969
C 0 -0.5140053798 1.0220737268 0.5771405178
H 0 0.3761411663 -0.6698603278 1.5520911128
H 0 -1.317594269 -0.3674091456 2.0034486859
S 0 2.1226922422 0.9930144881 -0.3204727035
C 0 -2.6561312983 1.1240295644 -0.1773661604
H 0 -2.9183205618 1.109295954 0.8835678291
C 0 2.4469708654 2.5675194384 0.4161207818
O 0 3.5195700954 2.8982447164 0.8666535199
H 0 1.5556748131 3.2399009258 0.4060789
H 0 -0.4986094608 1.8343783121 1.3138963342
C 0 0.3195593709 1.2112879193 -0.6384142535
H 0 0.1810643836 2.2039583822 -1.0926647706
H 0 0.0841355185 0.4529520586 -1.3981282329
H 0 -2.6952792984 2.1001092033 -0.6682637913

TS_exo_a
O 0 1.9038067533 -1.5114051376 0.2268511914
C 0 2.9034951262 -0.5421727846 -0.0130713485
C 0 2.3385910383 0.7615204372 -0.5201225736
H 0 3.5827250202 -0.9709727188 -0.7654761228
H 0 3.4863461781 -0.3490290443 0.9089164877
H 0 1.8506302601 0.7234199765 -1.4985301661
C 0 0.9297678873 -0.9242961982 1.0749363718
C 0 0.3553214523 0.2950275761 0.4223858516
H 0 1.3761593376 -0.65668649 2.0514669243
H 0 0.1638383641 -1.6994988327 1.2501923289
H 0 0.1304057514 1.15475556 1.0620024264

S 0 -0.8429381919 1.5405185965 -1.7715869735
C 0 2.7851827245 1.9551351518 -0.0739518863
H 0 3.3875757119 2.0278990327 0.8353904798
H 0 2.4992747022 2.8872059853 -0.5650749763
C 0 -2.0555146276 2.3475418551 -0.7660081542
O 0 -2.522911195 3.4347041042 -1.0127998397
H 0 -2.3590130406 1.735026602 0.1164687267
C 0 -0.5627233491 0.068593109 -0.7346219126
H 0 -1.553102049 -0.2941307862 -0.3994749074
H 0 -0.1591969845 -0.707387964 -1.4028461177

TS_exo_b
O 0 -2.4516730932 -1.0156408473 -0.5420241489
C 0 -2.922066657 0.2608026649 -0.1337112093
C 0 -1.8950822885 1.0984402264 0.6075436996
H 0 -3.8062468782 0.1414515866 0.5220030085
H 0 -3.2442540497 0.782115332 -1.0464561494
H 0 -1.8811337802 1.0080009016 1.6979191554
C 0 -1.5792371186 -1.5347215748 0.4413111086
C 0 -0.4349473503 -0.5909637944 0.6223270341
H 0 -1.245916316 -2.5203980262 0.0719706598
H 0 -2.1030347913 -1.6944563809 1.4034078129
H 0 0.0280142139 -0.5283131111 1.6129553279
S 0 1.6382203532 -1.6941254054 -0.8583493889
C 0 -1.3605408633 2.2073767024 0.0532451961
H 0 -1.4719057158 2.4148465151 -1.0141934728
H 0 -0.7508835999 2.9006271782 0.636945393
C 0 2.8335810731 -1.2785314942 0.3781126926
O 0 3.8148536512 -1.9415990165 0.621970309
H 0 2.5966409172 -0.3215263147 0.9016931033
C 0 0.44455217 -0.3292012885 -0.546856581
H 0 -0.1476933778 -0.2500131939 -1.4691129726
H 0 1.0178770912 0.6032893508 -0.435556468

References

(1) Ohmura, N.; Nakamura, A.; Hamasaki, A.; Tokunaga, M. Hydrolytic Deallylation of N-Allyl Amides Catalyzed by PdII Complexes. *European J. Org. Chem.* **2008**, *2008* (30), 5042–5045. https://doi.org/10.1002/EJOC.200800771.

(2) Millet, A.; Baudoin, O. Palladium-Catalyzed γ-Selective Arylation of Zincated Boc-Allylamines. *Org. Lett.* **2014**, *16* (15), 3998–4000. https://doi.org/10.1021/OL5018257/SUPPL_FILE/OL5018257_SI_001.PDF.

(3) Yotphan, S.; Sumunnee, L.; Beukeaw, D.; Buathongjan, C.; Reutrakul, V. Iodine-Catalyzed Expeditious Synthesis of Sulfonamides from Sulfonyl Hydrazides and Amines. *Org. Biomol. Chem.* **2015**, *14* (2), 590–597. https://doi.org/10.1039/C5OB02075A.

(4) Nugent, W. A.; Feldman, J.; Calabrese, J. C. Practical Catalyst for Cyclic Metathesis. Synthesis of Functional and/or Enantiopure Cycloalkenes. *J. Am. Chem. Soc.* **1995**, *117* (35), 8992–8998. https://doi.org/10.1021/JA00140A015/SUPPL_FILE/JA8992.PDF.

(5) Kozłowska, A.; Dranka, M.; Zachara, J.; Pump, E.; Slugovc, C.; Skowerski, K.; Grela, K. Chelating Ruthenium Phenolate Complexes: Synthesis, General Catalytic Activity, and Applications in Olefin Metathesis Polymerization. *Chem. – A Eur. J.* **2014**, *20* (43), 14120–14125. https://doi.org/10.1002/chem.201403580.

(6) De Riggi, I.; Gastaldi, S.; Surzur, J. M.; Bertrand, M. P.; Virgili, A. Chemoselective Ring Construction from Unsymmetrical 1,6-Dienes via Radical Addition of Sulfonyl Halides. *J. Org. Chem.* **1992**, *57* (23), 6118–6125. https://doi.org/10.1021/JO00049A014/SUPPL_FILE/JO00049A014_SI_001.PDF.

(7) Zieliński, G. K.; Samojłowicz, C.; Wdowik, T.; Grela, K. In Tandem or Alone: A Remarkably Selective Transfer Hydrogenation of Alkenes Catalyzed by Ruthenium Olefin Metathesis Catalysts. *Org. Biomol. Chem.* **2015**, *13* (9), 2684–2688. https://doi.org/10.1039/C4OB02480J.

(8) Mandal, J.; Krishna Prasad, S.; Rao, D. S. S.; Ramakrishnan, S. Periodically Clickable Polyesters: Study of Intrachain Self-Segregation Induced Folding, Crystallization, and Mesophase Formation. *J. Am. Chem. Soc.* **2014**, *136* (6), 2538–2545. https://doi.org/10.1021/JA411583F/SUPPL_FILE/JA411583F_SI_001.PDF.

(9) Nečas, D.; Turský, M.; Kotora, M. Catalytic Deallylation of Allyl- and Diallylmalonates. *J. Am. Chem. Soc.* **2004**, *126* (33), 10222–10223. https://doi.org/10.1021/JA047320T/SUPPL_FILE/JA047320TSI20040712_080435.PDF.

(10) Grigg, R.; Malone, J. F.; Mitchell, T. R. B.; Ramasubbu, A.; Scott, R. M. Palladium- and Rhodium-Catalysed Cyclisation of 1,6-, 1,7- and 1,8-Dienes to Cyclopentenes and Methylenecyclopentenes. Crystal Structure of Dichloro(4,4-Diacetylhepta-1,6-Diene)Platinum(II). *J. Chem. Soc. Perkin Trans. 1* **1984**, No. 0, 1745–1754. https://doi.org/10.1039/P19840001745.

(11) Chow, H. F.; Ng, K. F.; Wang, Z. Y.; Wong, C. H.; Luk, T.; Lo, C. M.; Yang, Y. Y. Synthesis of New Amphiphilic Dendrons Bearing Aliphatic Hydrocarbon Surface Sectors and a Monocarboxylic or Dicarboxylic Acid Focal Point. *Org. Lett.* **2006**, *8* (3), 471–474. https://doi.org/10.1021/OL0526928/SUPPL_FILE/OL0526928SI20051222_100443.PDF.

(12) Kirkland, T. A.; Grubbs, R. H. Effects of Olefin Substitution on the Ring-Closing Metathesis of Dienes. *J. Org. Chem.* **1997**, *62* (21), 7310–7318. https://doi.org/10.1021/JO970877P/SUPPL_FILE/JO7310.PDF.

(13) Ashikari, Y.; Nokami, T.; Yoshida, J. I. Integration of Electrooxidative Cyclization and Chemical Oxidation via Alkoxysulfonium Ions. Synthesis of Exocyclic Ketones from Alkenes with Cyclization. *Org. Biomol. Chem.* **2013**, *11* (20), 3322–3331. https://doi.org/10.1039/C3OB40315G.
(14) McLean, J. T.; Milbeo, P.; Lynch, D. M.; McSweeney, L.; Scanlan, E. Radical-Mediated Acyl-Thiol-Ene Reaction for Rapid Synthesis of Biomolecular Thioester Derivatives. *European J. Org. Chem.* **2021**, ejoc.202100615. https://doi.org/10.1002/EJOC.202100615.
(15) Nolan, M. D.; Mezzetta, A.; Guazzelli, L.; Scanlan, E. M. Radical-Mediated Thiol-Ene 'Click' Reactions in Deep Eutectic Solvents for Bioconjugation. *Green Chem.* **2022**, *24* (4), 1456–1462. https://doi.org/10.1039/d1gc03714e.
(16) Padwa, A.; Nimmesgern, H.; Wong, G. S. K. Synthesis of the Pyrrolidine Ring System by Radical Cyclization. *J. Org. Chem.* **1985**, *50* (26), 5620–5627. https://doi.org/10.1021/JO00350A038/ASSET/JO00350A038.FP.PNG_V03.
(17) Böing, C.; Hahne, J.; Franciò, G.; Leitner, W. Stereoselective Nickel-Catalyzed Cycloisomerization of 1,6-Dienes. *Adv. Synth. Catal.* **2008**, *350* (7–8), 1073–1080. https://doi.org/10.1002/ADSC.200800104.
(18) Frisch, M. J.; Trucks, G. W.; Schlegel, H. B.; Scuseria, G. E.; Robb, M. a.; Cheeseman, J. R.; Scalmani, G.; Barone, V.; Petersson, G. a.; Nakatsuji, H.; Li, X.; Caricato, M.; Marenich, a. V.; Bloino, J.; Janesko, B. G.; Gomperts, R.; Mennucci, B.; Hratchian, H. P.; Ortiz, J. V.; Izmaylov, a. F.; Sonnenberg, J. L.; Williams; Ding, F.; Lipparini, F.; Egidi, F.; Goings, J.; Peng, B.; Petrone, A.; Henderson, T.; Ranasinghe, D.; Zakrzewski, V. G.; Gao, J.; Rega, N.; Zheng, G.; Liang, W.; Hada, M.; Ehara, M.; Toyota, K.; Fukuda, R.; Hasegawa, J.; Ishida, M.; Nakajima, T.; Honda, Y.; Kitao, O.; Nakai, H.; Vreven, T.; Throssell, K.; Montgomery Jr., J. a.; Peralta, J. E.; Ogliaro, F.; Bearpark, M. J.; Heyd, J. J.; Brothers, E. N.; Kudin, K. N.; Staroverov, V. N.; Keith, T. a.; Kobayashi, R.; Normand, J.; Raghavachari, K.; Rendell, a. P.; Burant, J. C.; Iyengar, S. S.; Tomasi, J.; Cossi, M.; Millam, J. M.; Klene, M.; Adamo, C.; Cammi, R.; Ochterski, J. W.; Martin, R. L.; Morokuma, K.; Farkas, O.; Foresman, J. B.; Fox, D. J. G16_C01. 2016, p Gaussian 16, Revision C.01, Gaussian, Inc., Wallin.
(19) Head-Gordon, M.; Head-Gordon, T. Analytic MP2 Frequencies without Fifth-Order Storage. Theory and Application to Bifurcated Hydrogen Bonds in the Water Hexamer. *Chem. Phys. Lett.* **1994**, *220* (1–2), 122–128. https://doi.org/10.1016/0009-2614(94)00116-2.
(20) Weigend, F. Accurate Coulomb-Fitting Basis Sets for H to Rn. *Phys. Chem. Chem. Phys.* **2006**, *8* (9), 1057–1065. https://doi.org/10.1039/B515623H.
(21) Marenich, A. V.; Cramer, C. J.; Truhlar, D. G. Universal Solvation Model Based on Solute Electron Density and on a Continuum Model of the Solvent Defined by the Bulk Dielectric Constant and Atomic Surface Tensions. *J. Phys. Chem. B* **2009**, *113* (18), 6378–6396. https://doi.org/10.1021/JP810292N/SUPPL_FILE/JP810292N_SI_003.PDF.

NMR Spectra of Novel Compounds

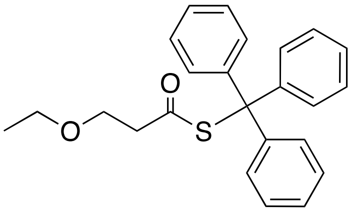

17b ^{1}H NMR (400 MHz, $CDCl_3$)

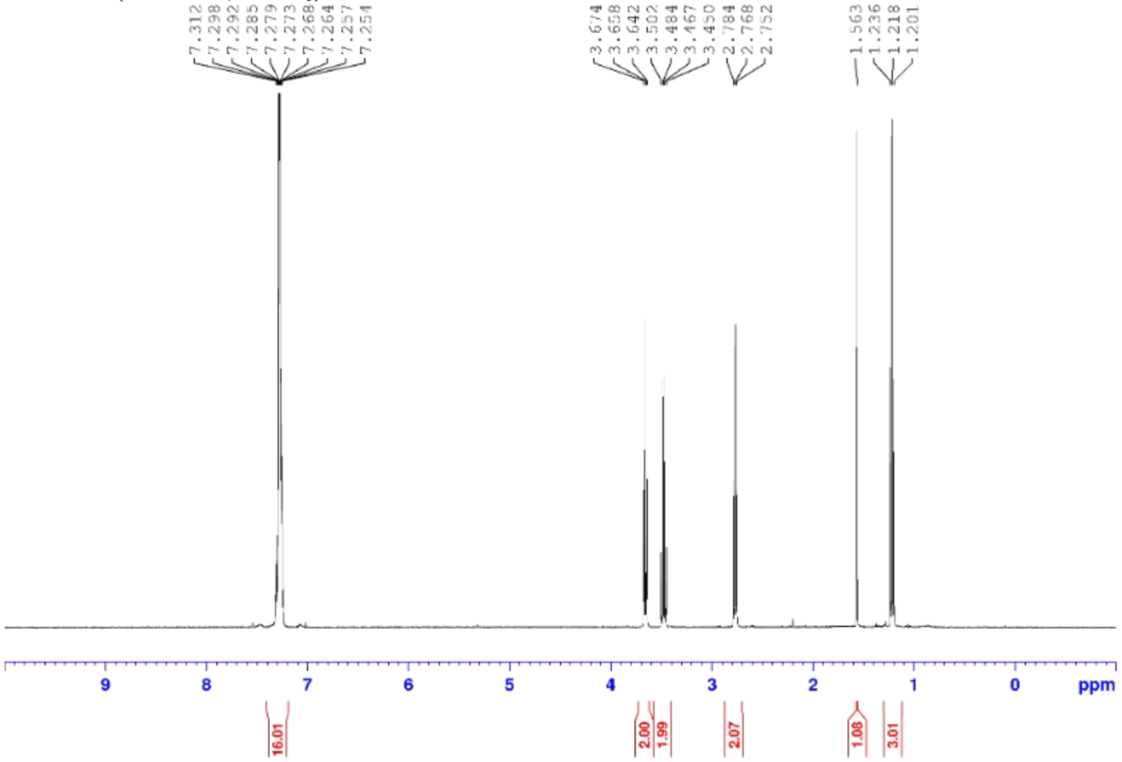

17b $^{13}C\{^{1}H\}$ NMR (151 MHz, $CDCl_3$)

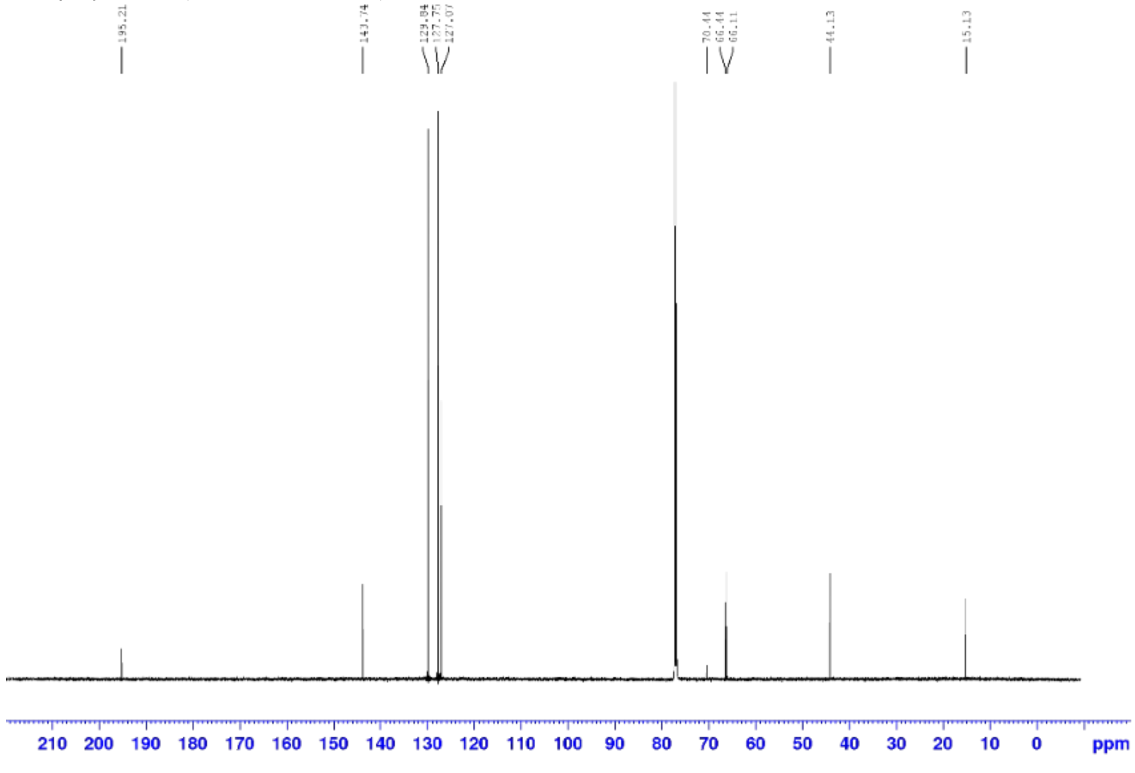

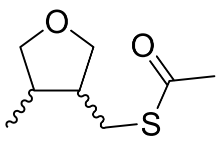

2b ^{1}H NMR (400 MHz, $CDCl_3$)

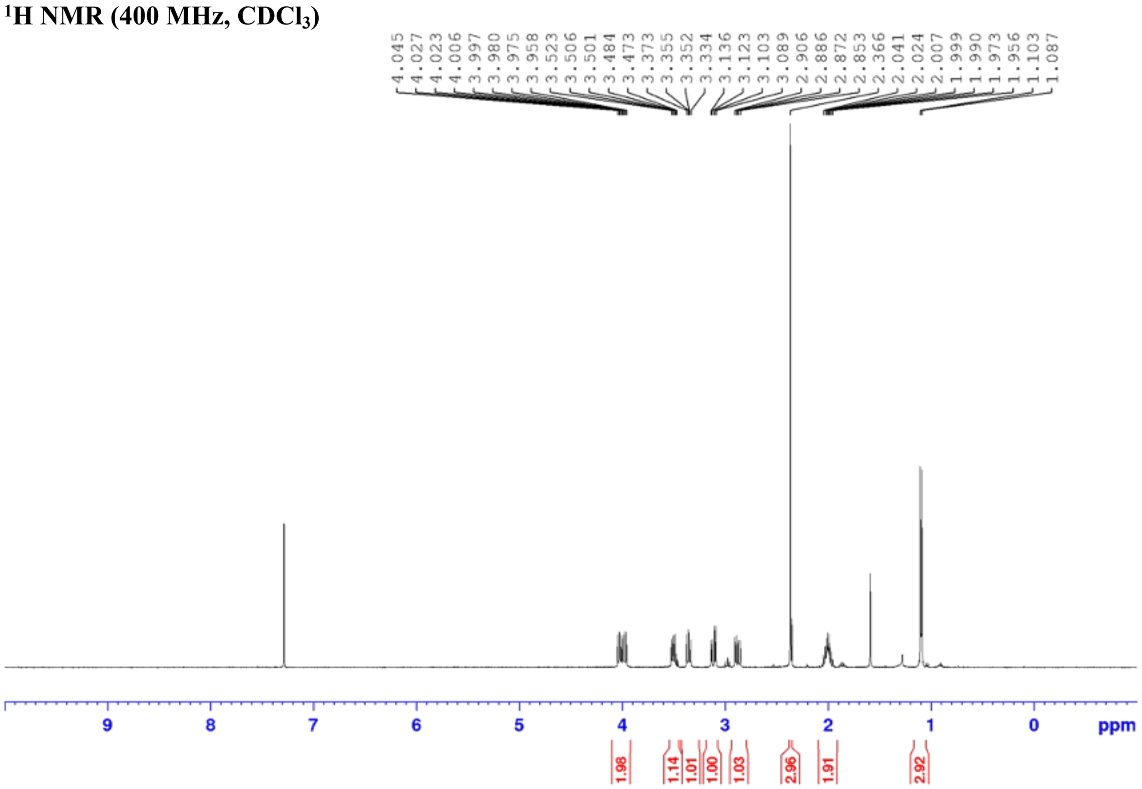

2b $^{13}C\{^{1}H\}$ NMR (151 MHz, $CDCl_3$)

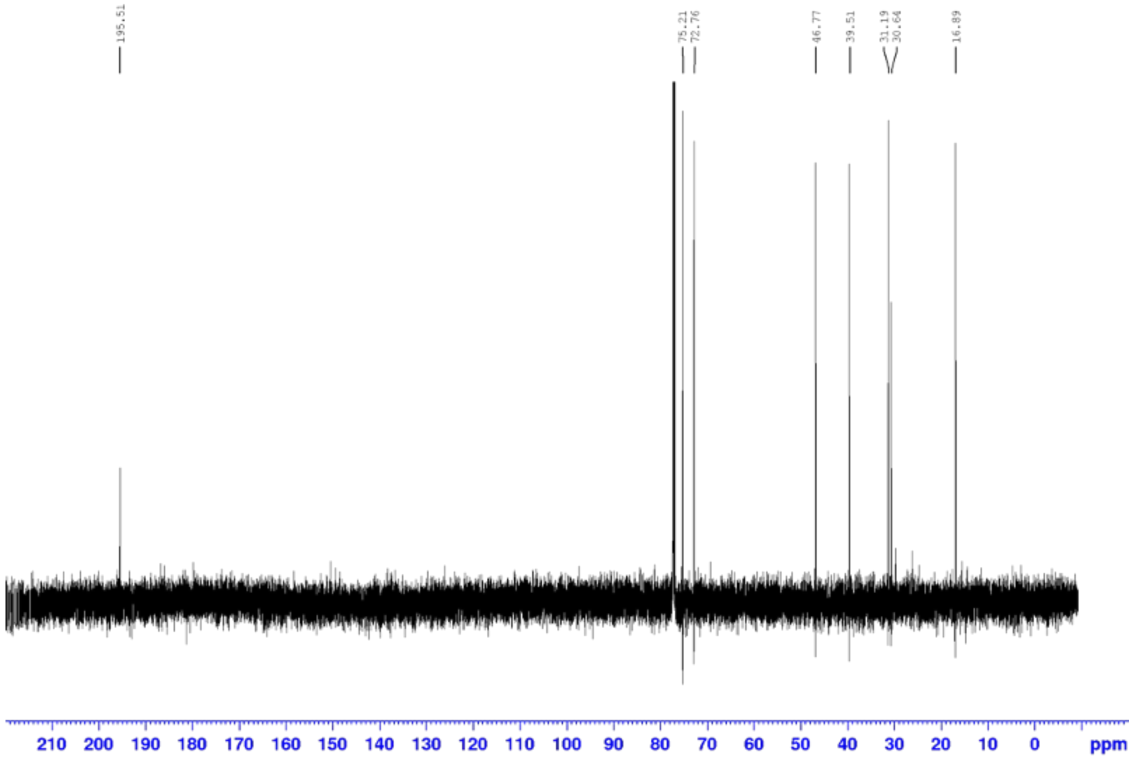

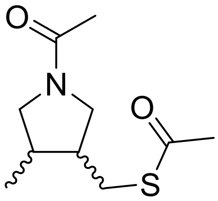

2c ^{1}H NMR (400 MHz, $CDCl_3$)

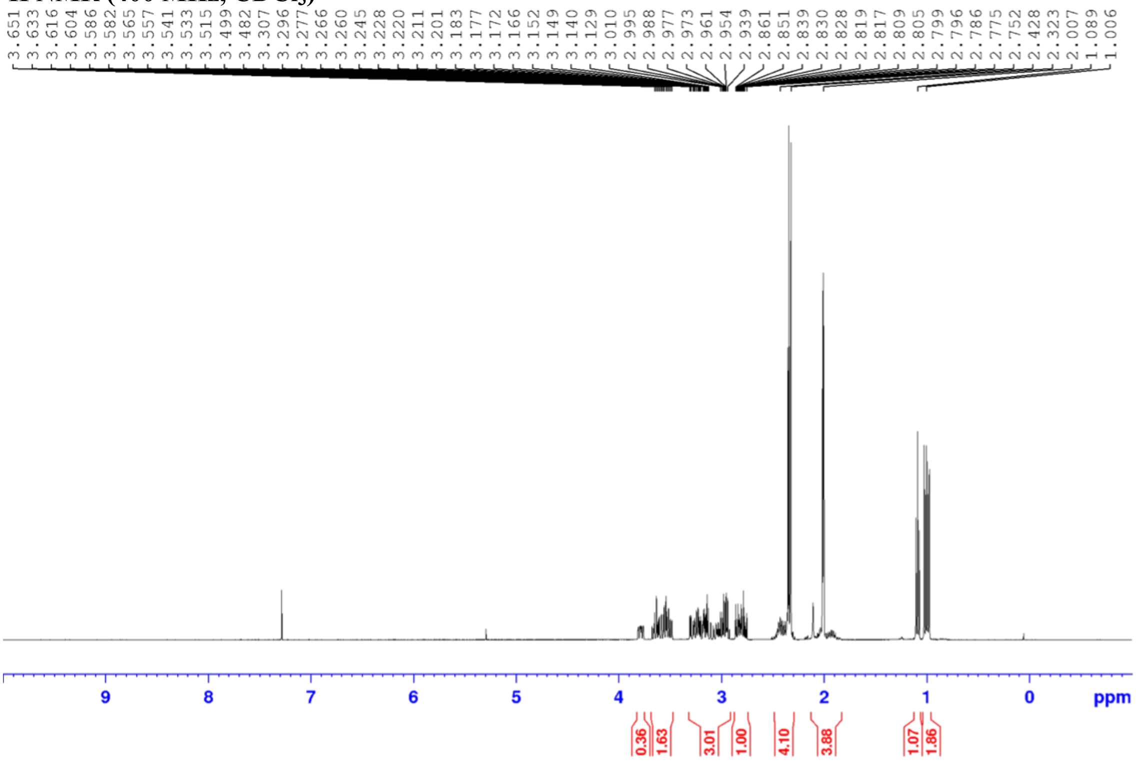

2c ^{13}C{^{1}H} NMR (151 MHz, $CDCl_3$)

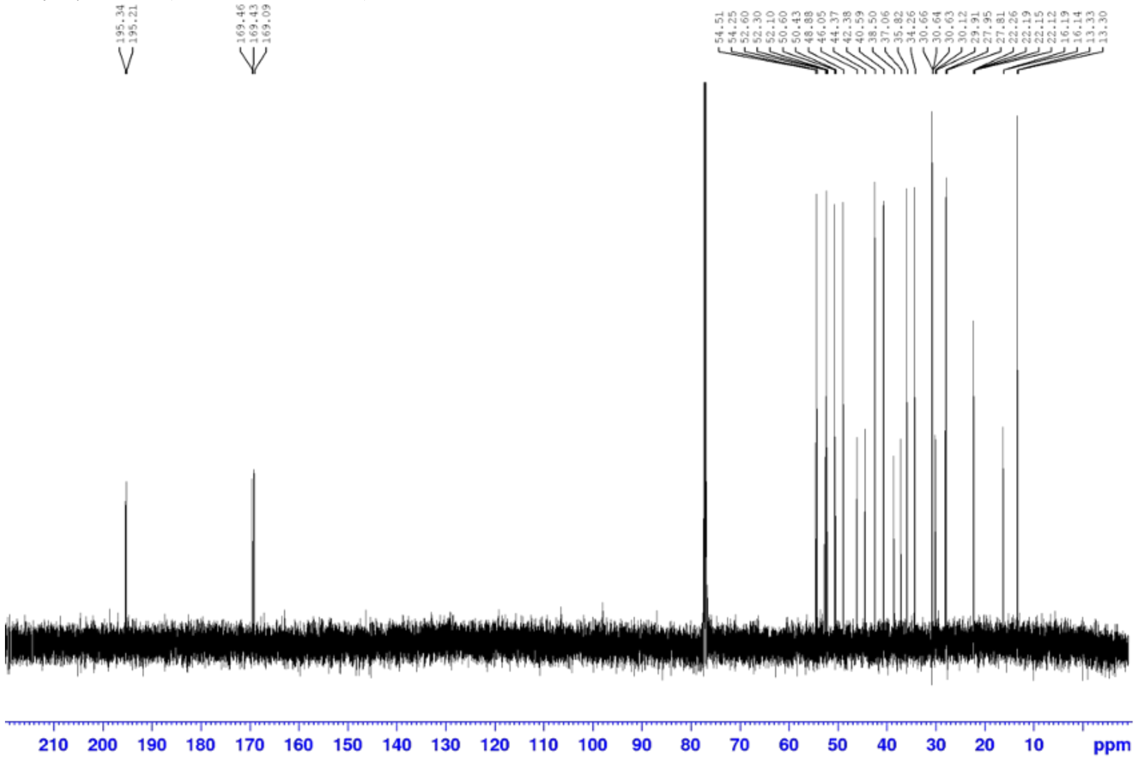

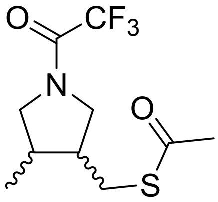

2d ^{1}H NMR (400 MHz, $CDCl_3$)

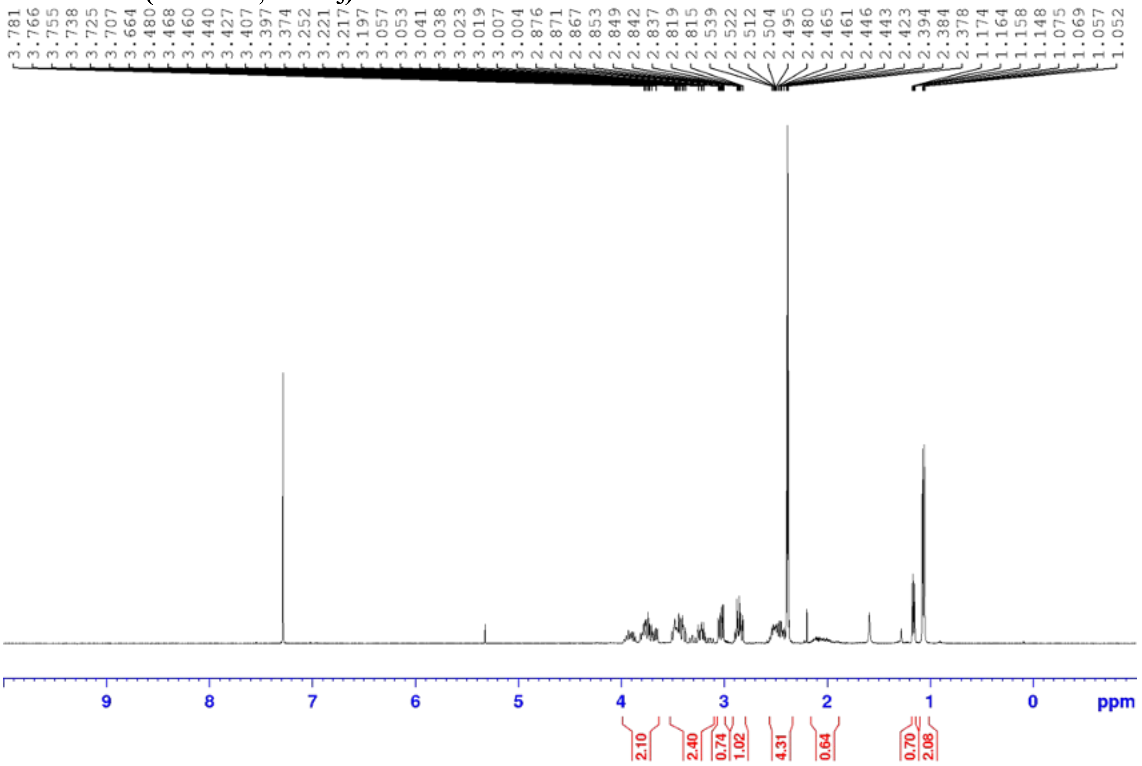

2d $^{13}C\{^{1}H\}$ NMR (151 MHz, $CDCl_3$)

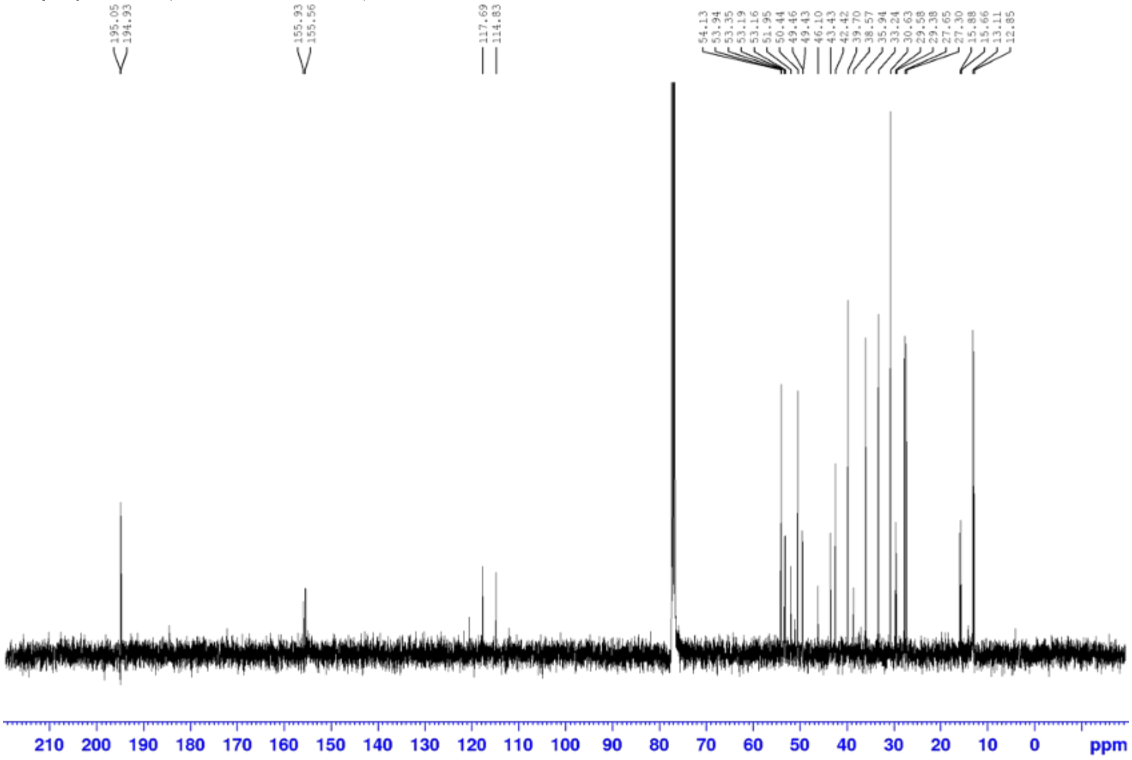

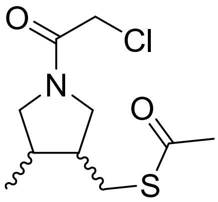

2e ^{1}H NMR (400 MHz, $CDCl_3$)

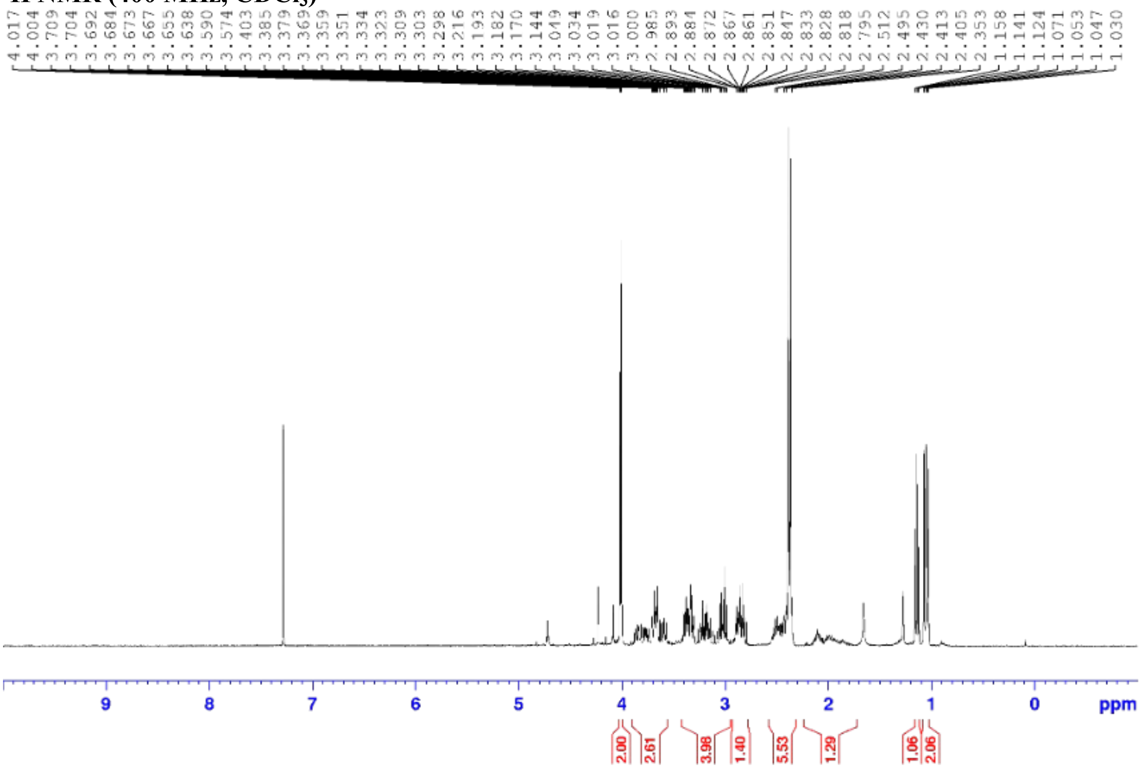

2e $^{13}C\{^1H\}$ NMR (151 MHz, $CDCl_3$)

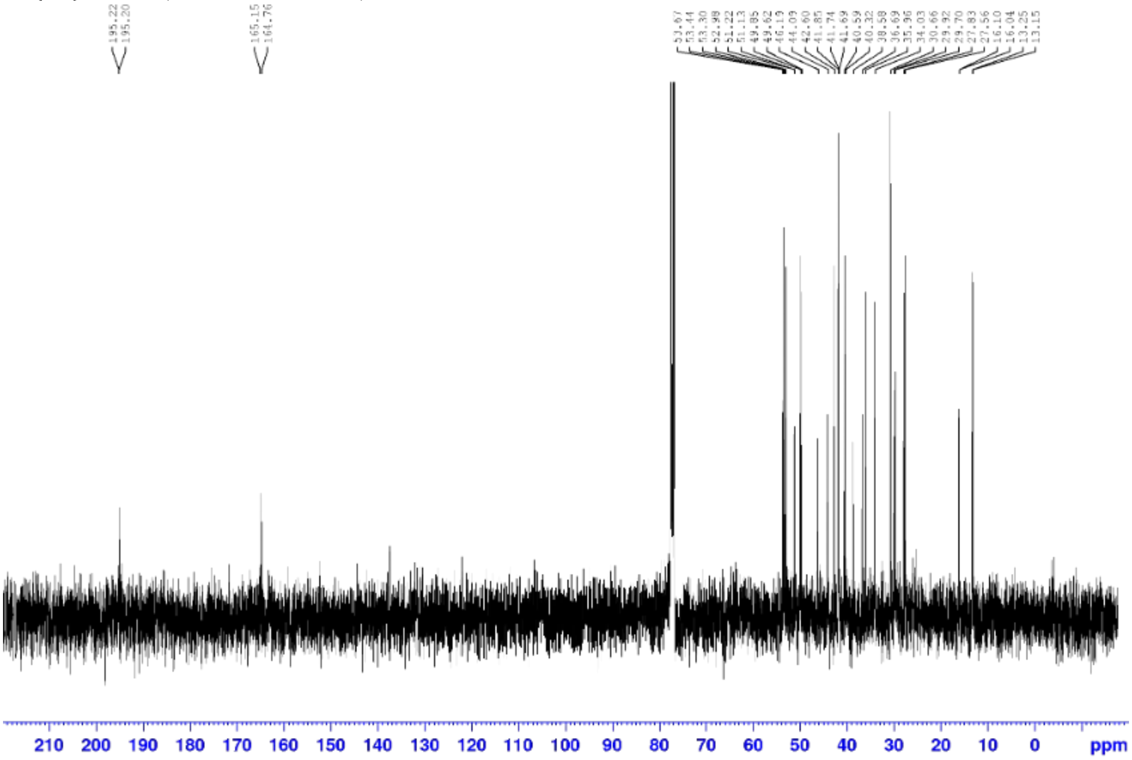

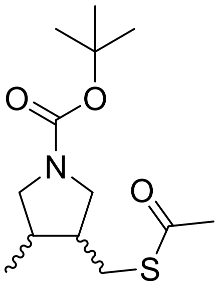

2f ^{1}H NMR (400 MHz, CDCl$_3$)

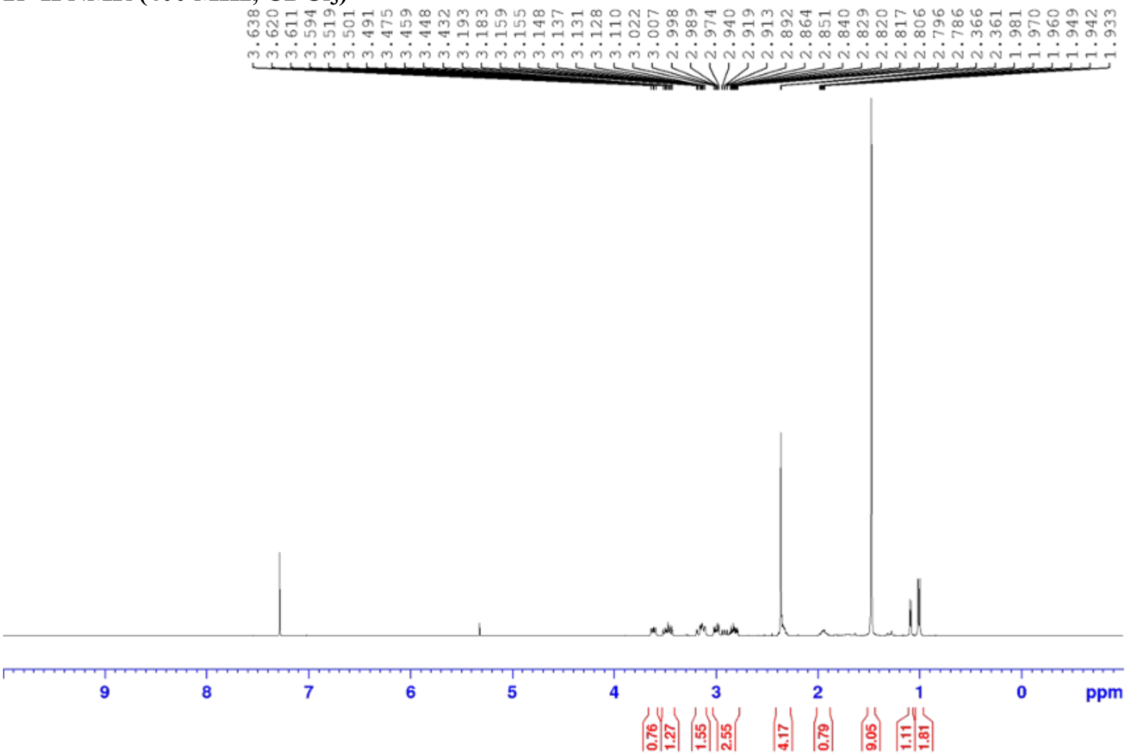

2f ^{13}C{^{1}H} NMR (151 MHz, CDCl$_3$)

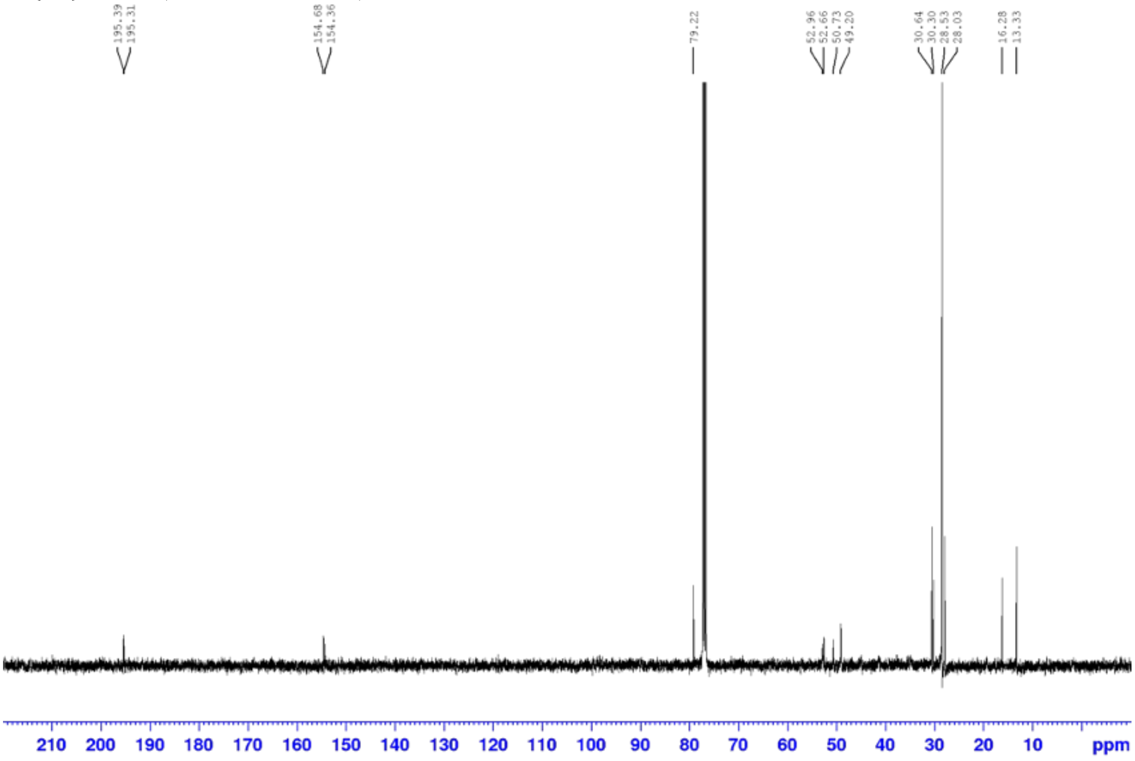

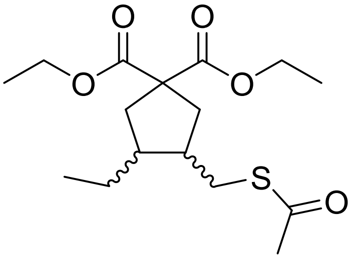

2h ^{1}H NMR (400 MHz, $CDCl_3$)

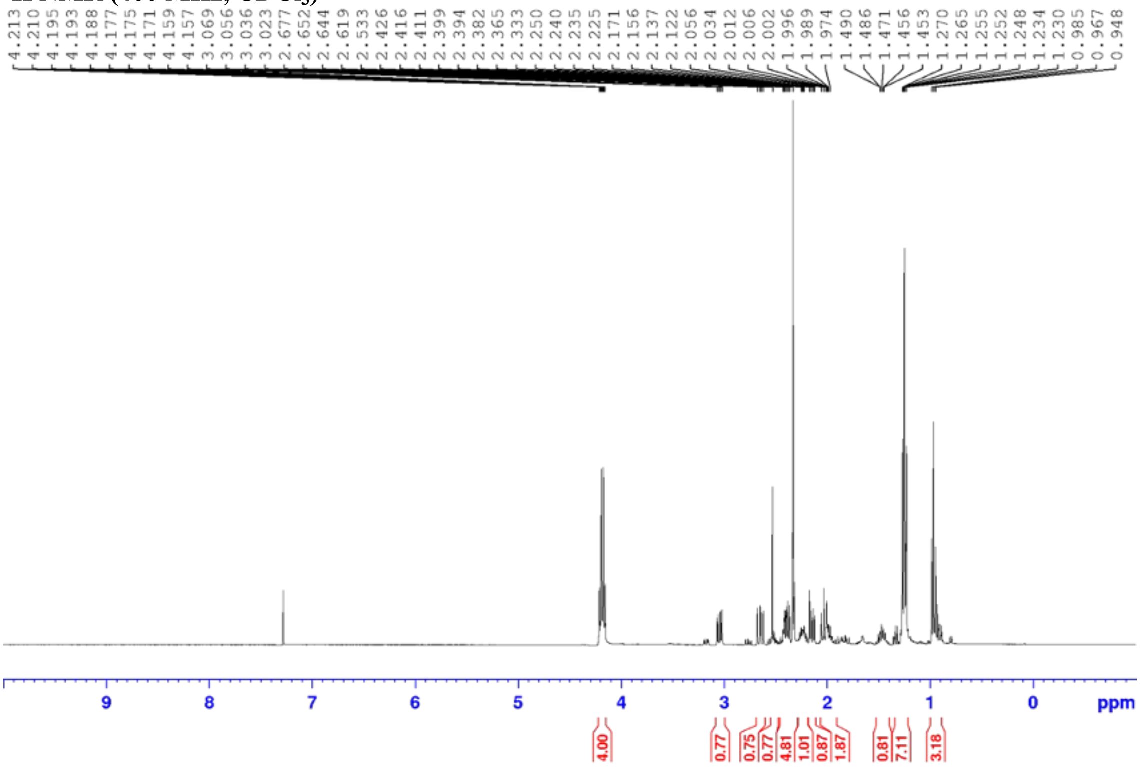

2h $^{13}C\{^{1}H\}$ NMR (151 MHz, $CDCl_3$)

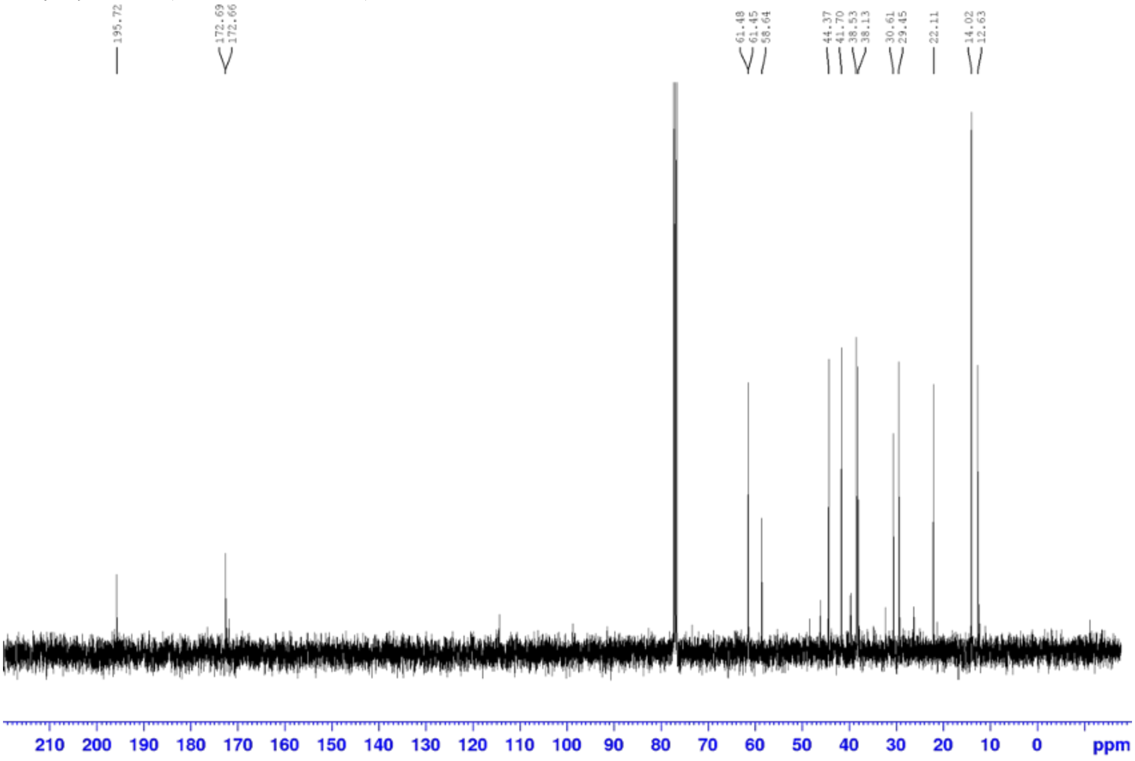

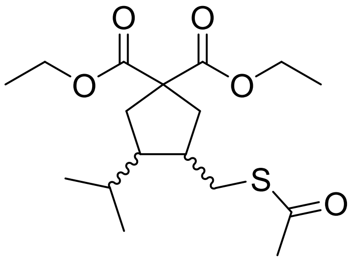

2i ^{1}H NMR (400 MHz, $CDCl_3$)

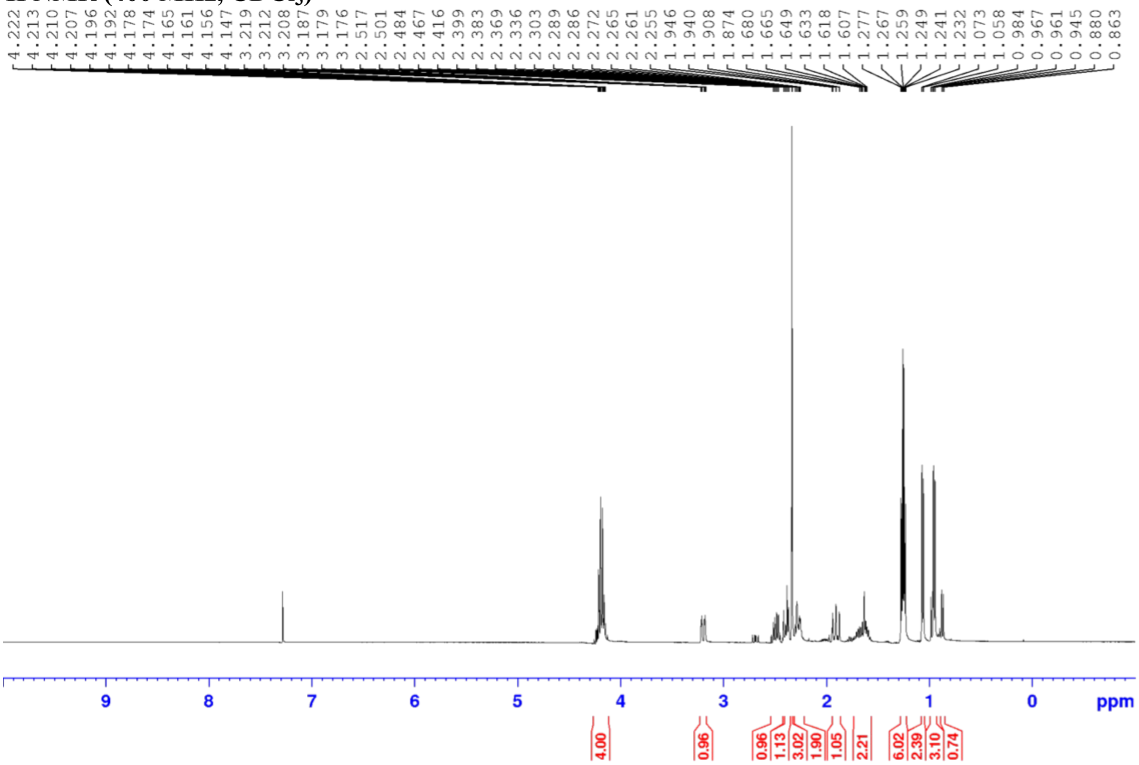

2i $^{13}C\{^{1}H\}$ NMR (151 MHz, $CDCl_3$)

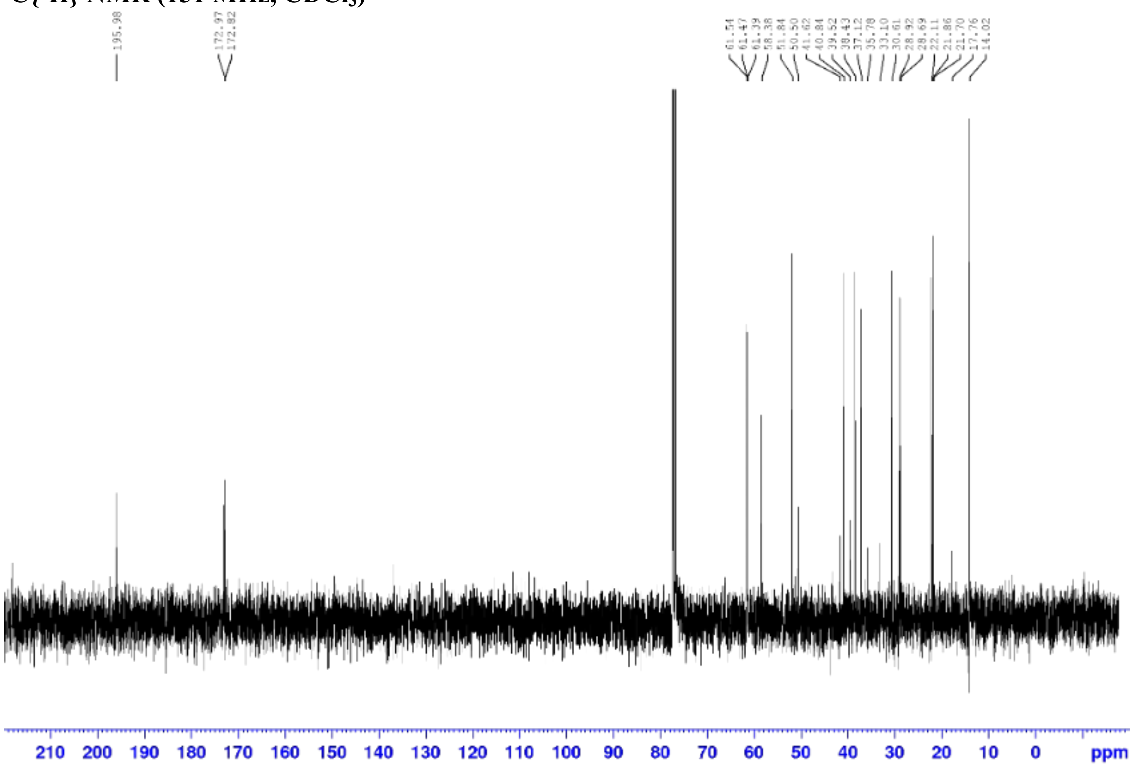

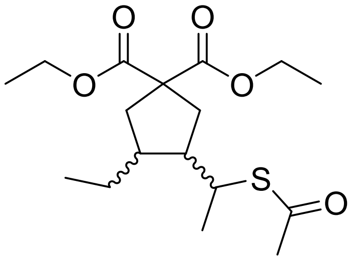

2j ^{1}H NMR (400 MHz, $CDCl_3$)

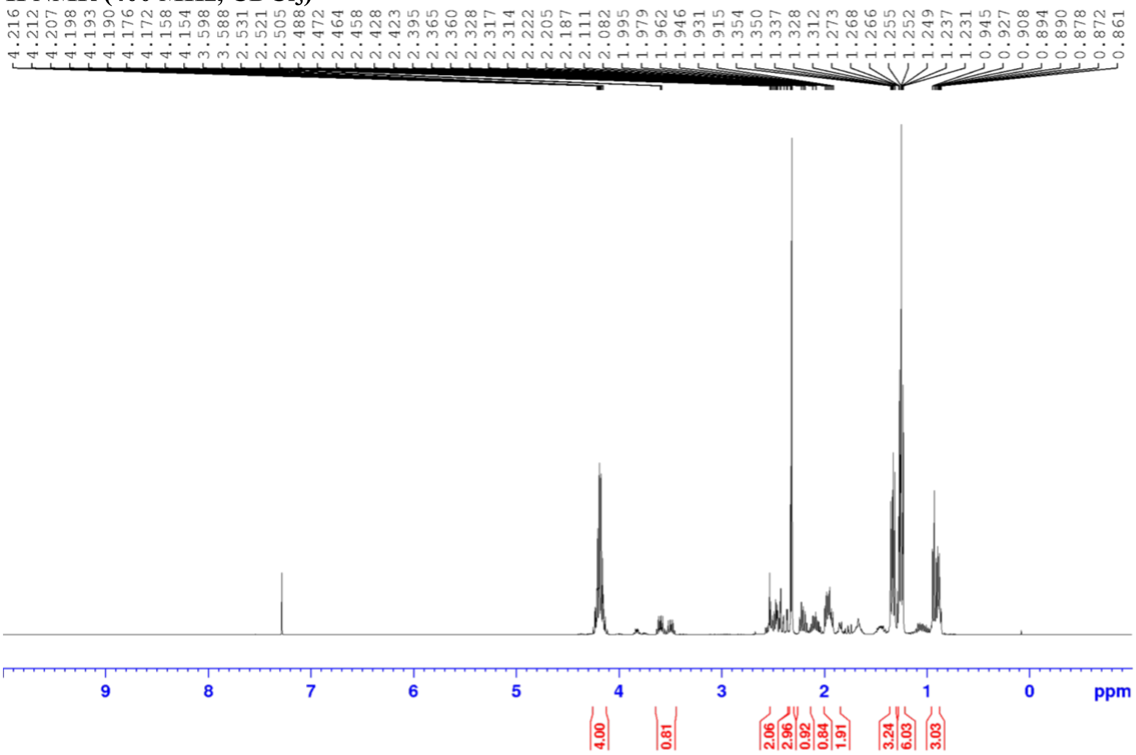

2j ^{13}C{^{1}H} NMR (151 MHz, $CDCl_3$)

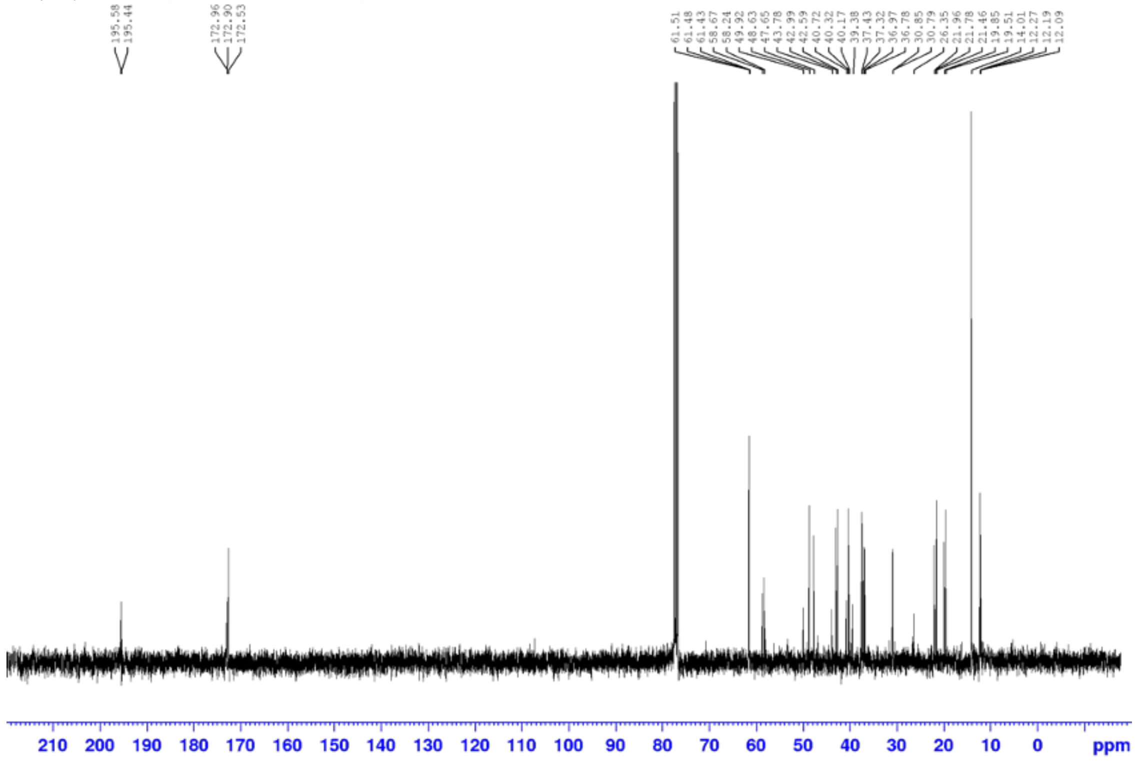

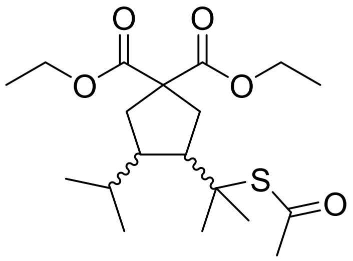

2k ^{1}H NMR (400 MHz, CDCl$_3$)

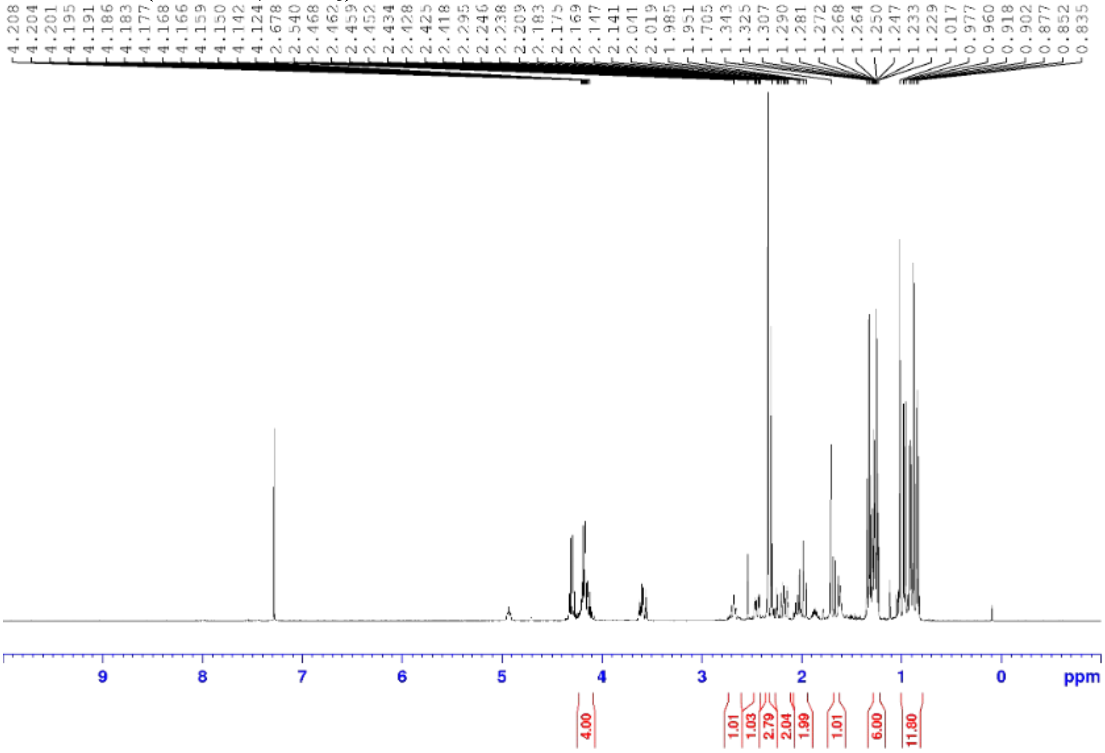

2k ^{13}C{^{1}H} NMR (151 MHz, CDCl$_3$)

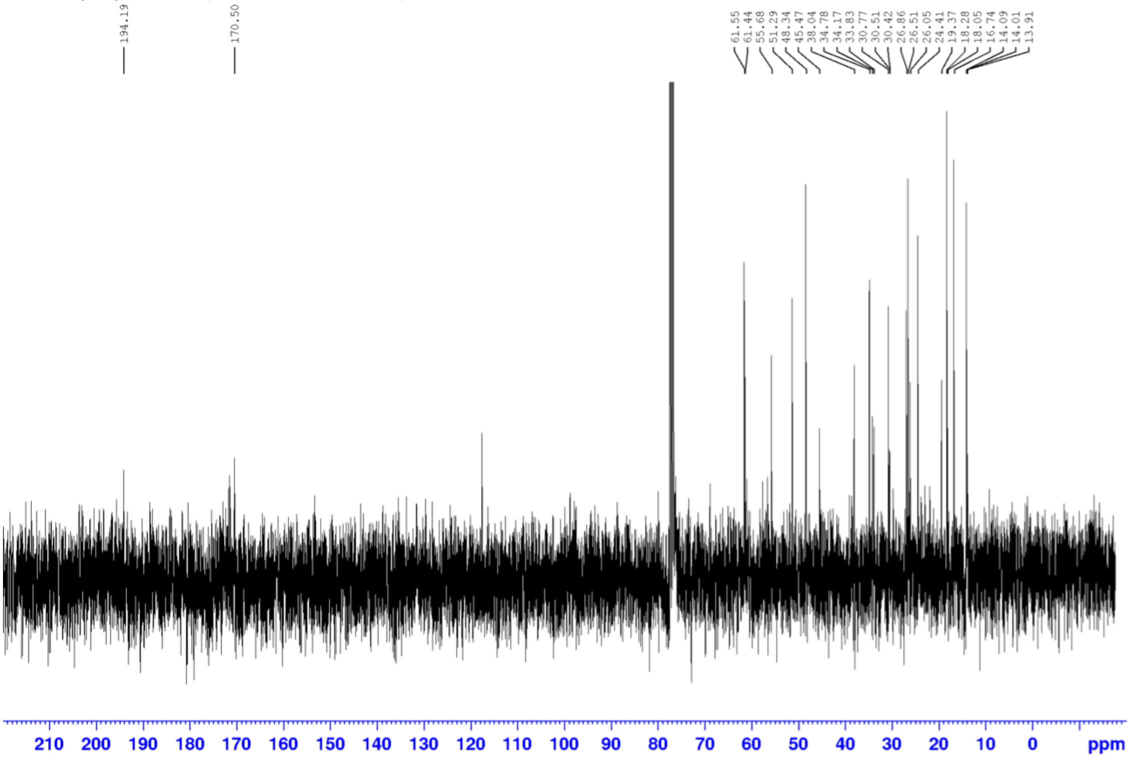

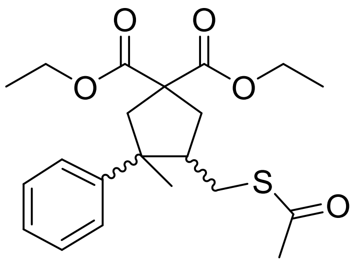

2l ^{1}H NMR (400 MHz, $CDCl_3$)

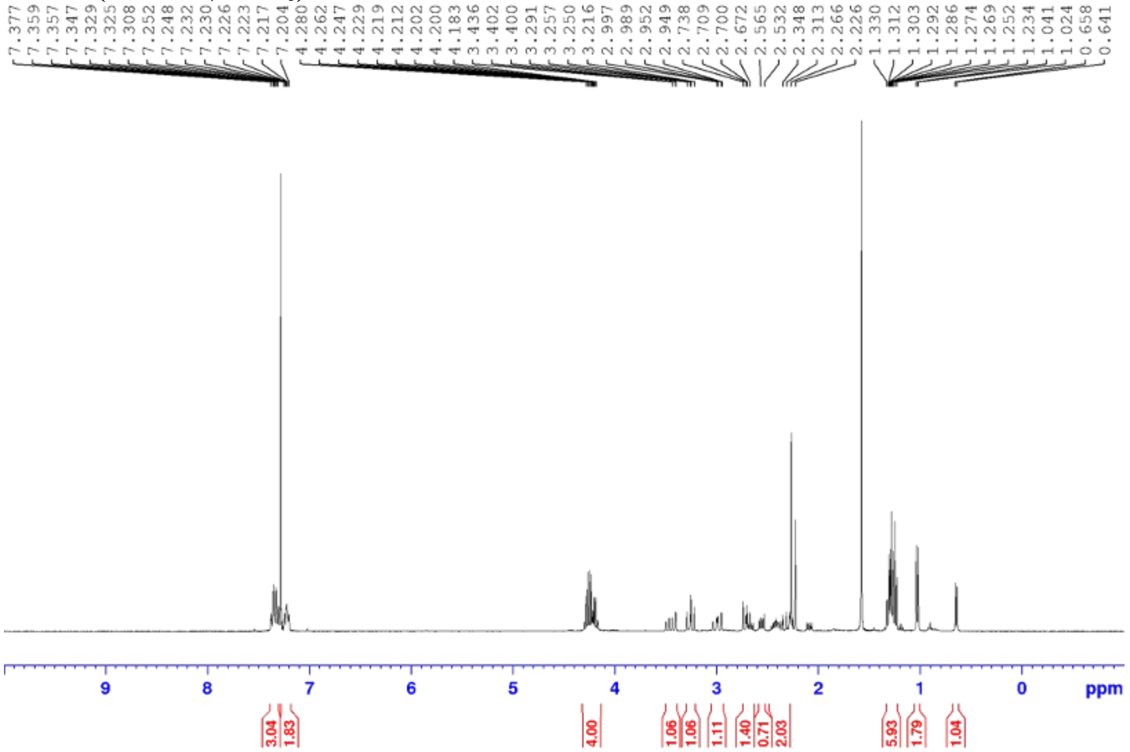

2l $^{13}C\{^{1}H\}$ NMR (151 MHz, $CDCl_3$)

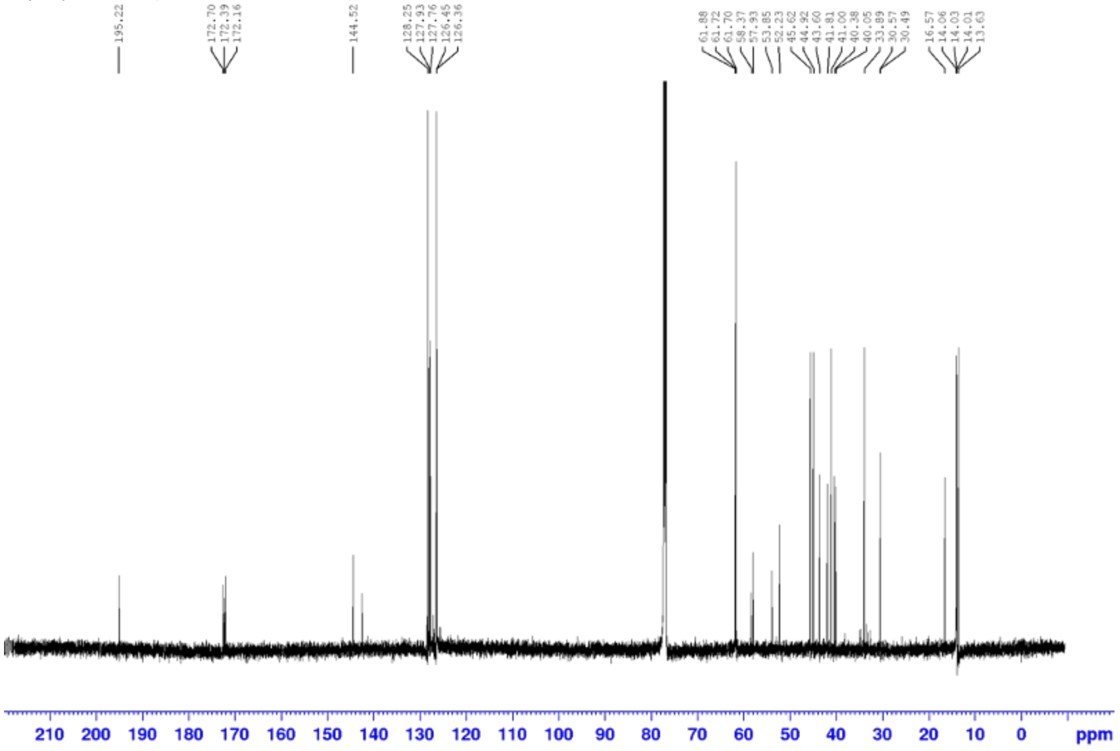

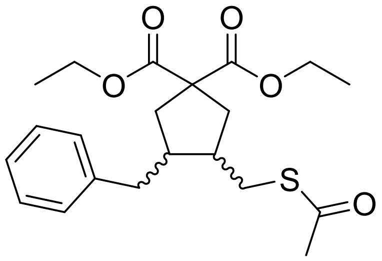

2m ^{1}H NMR (400 MHz, CDCl$_3$)

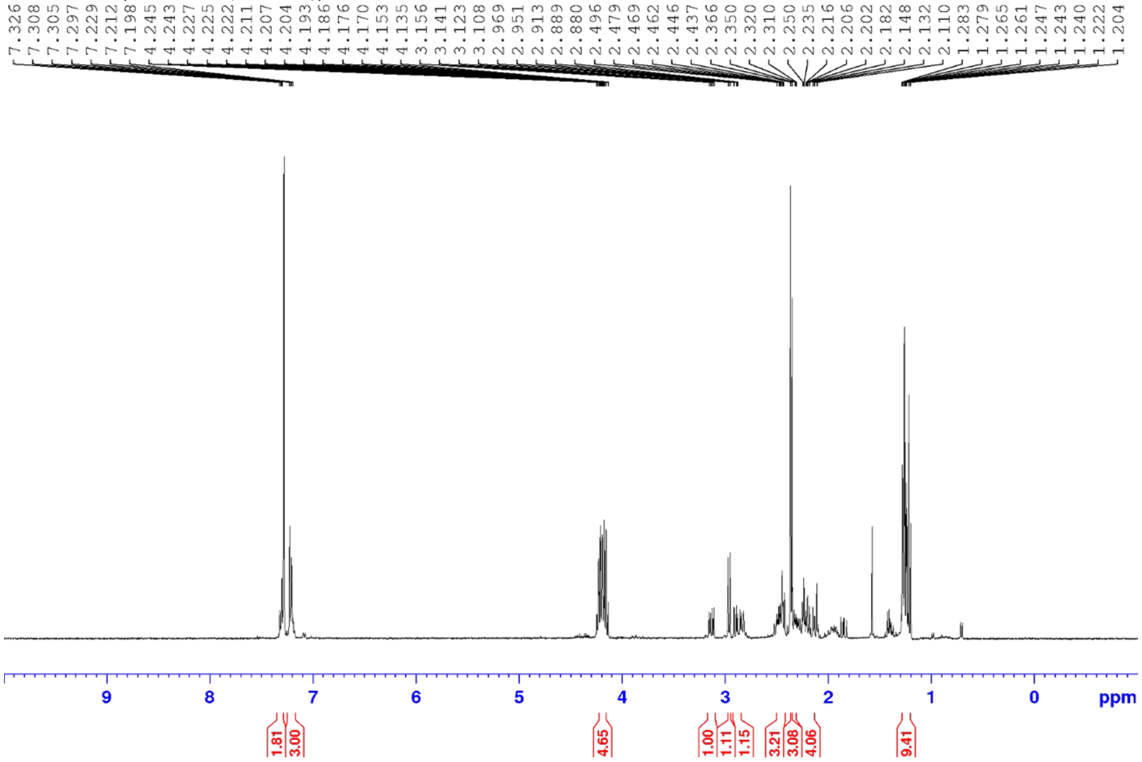

2m ^{13}C{^{1}H} NMR (151 MHz, CDCl$_3$)

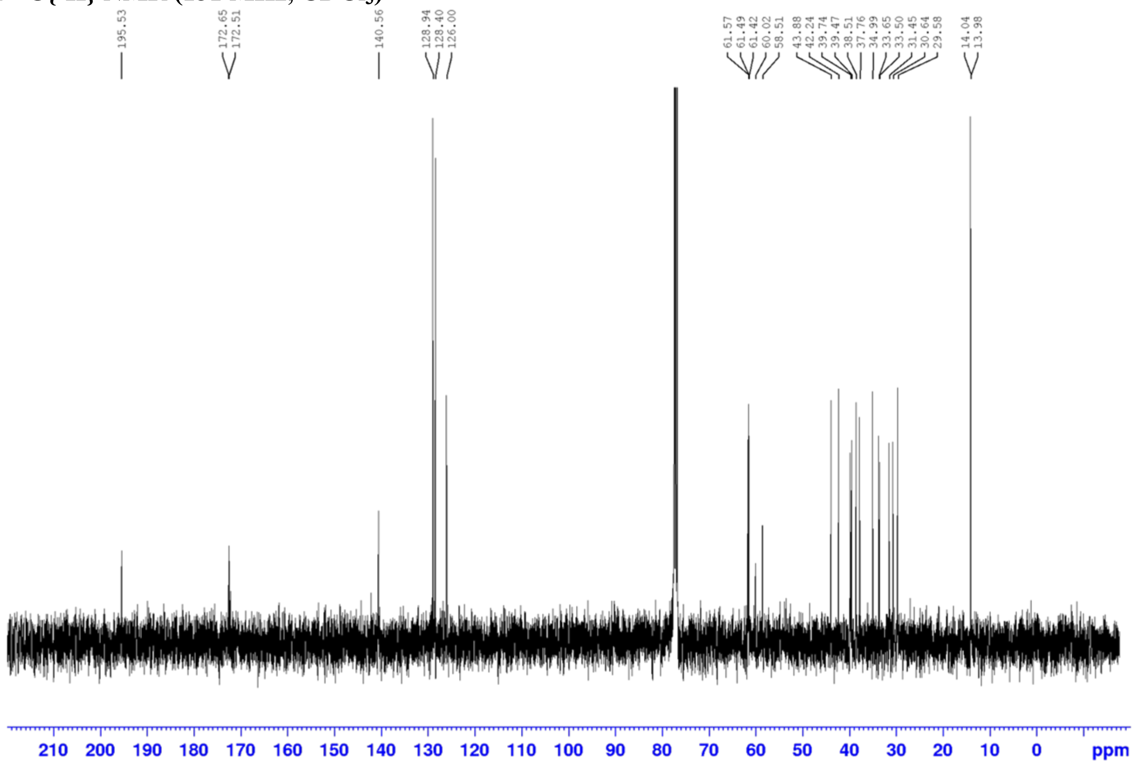

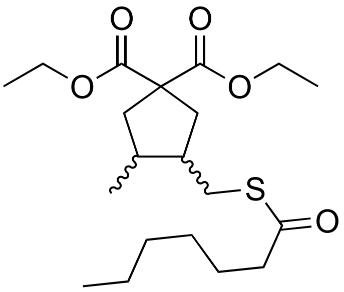

3a ^{1}H NMR (400 MHz, $CDCl_3$)

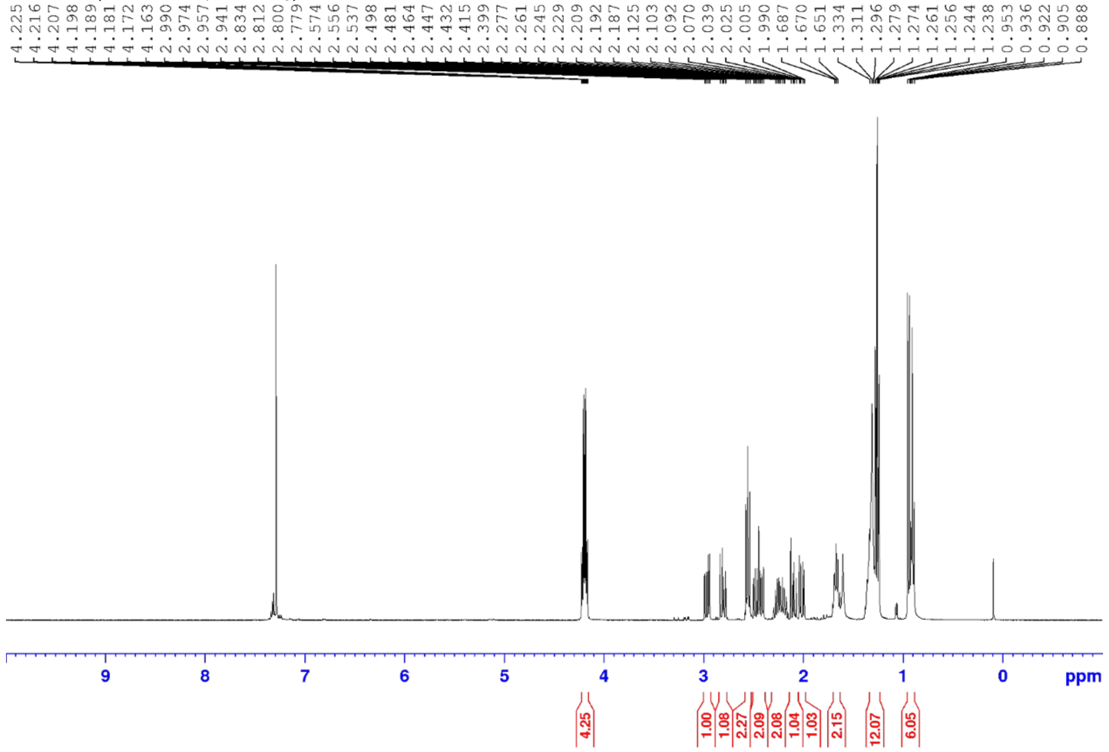

3a $^{13}C\{^{1}H\}$ NMR (151 MHz, $CDCl_3$)

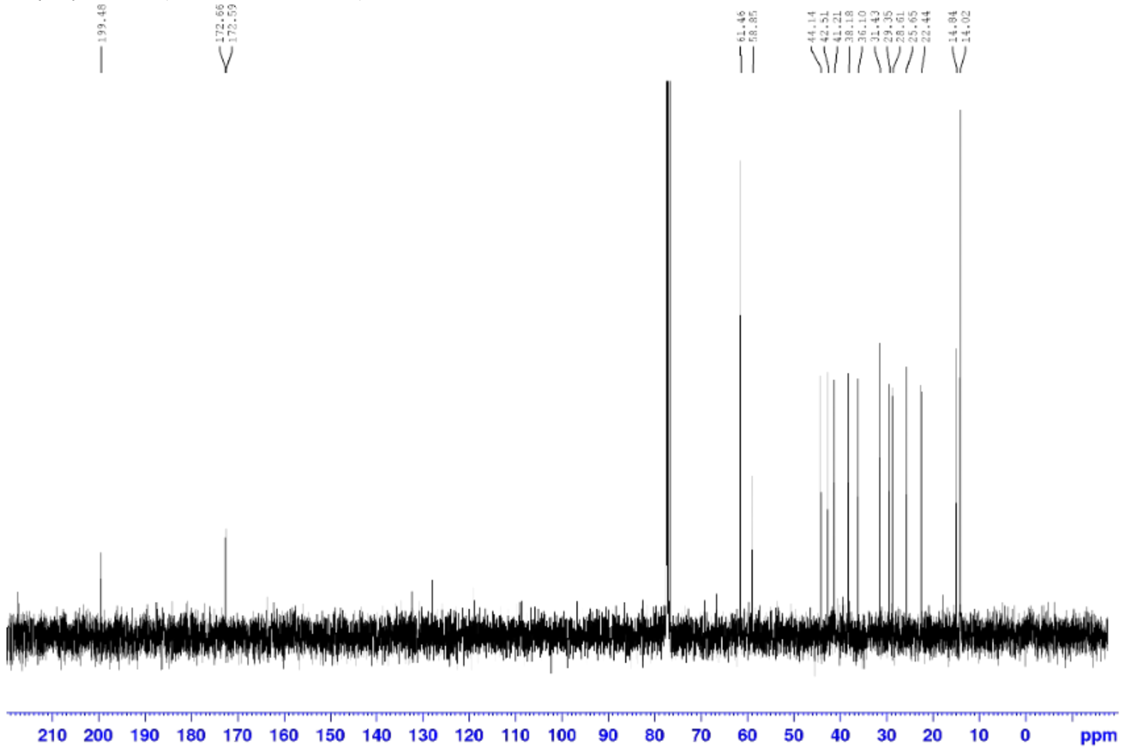

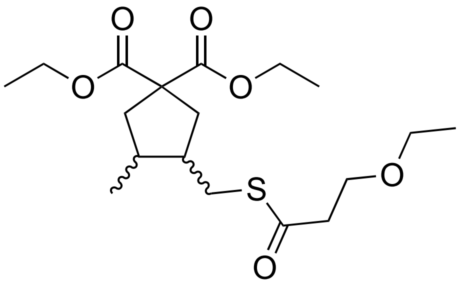

3b ^{1}H NMR (400 MHz, CDCl$_3$)

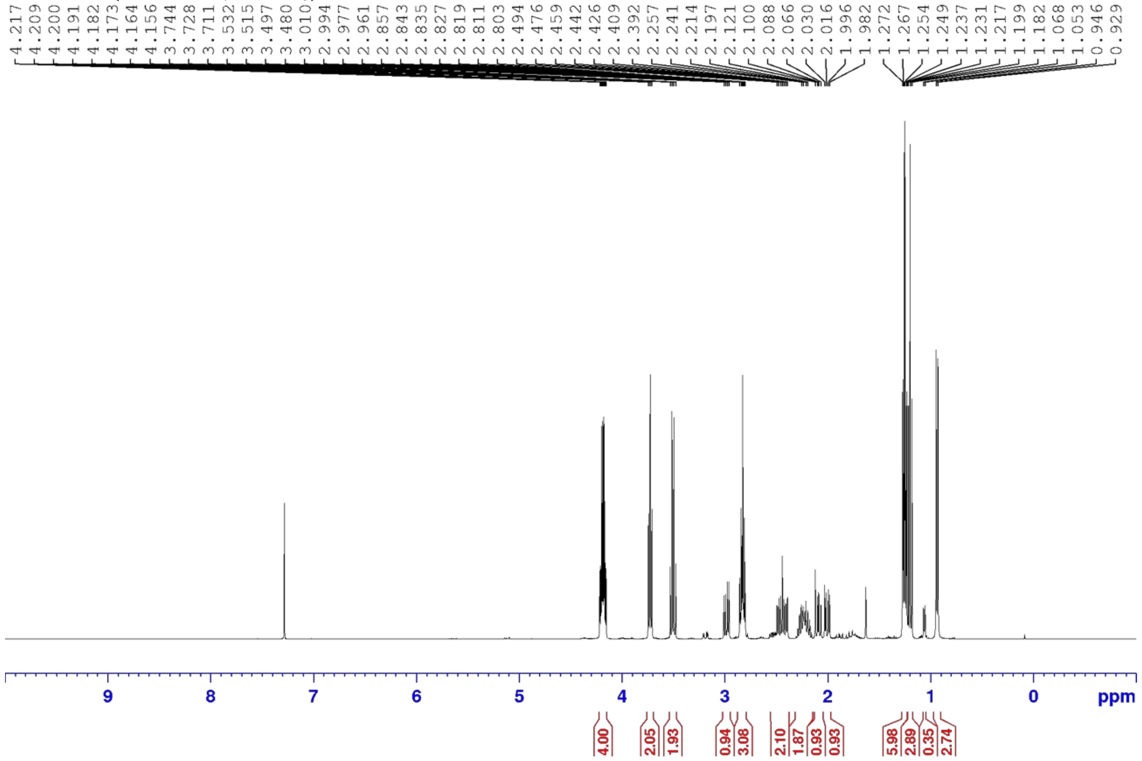

3b ^{13}C{^{1}H} NMR (151 MHz, CDCl$_3$)

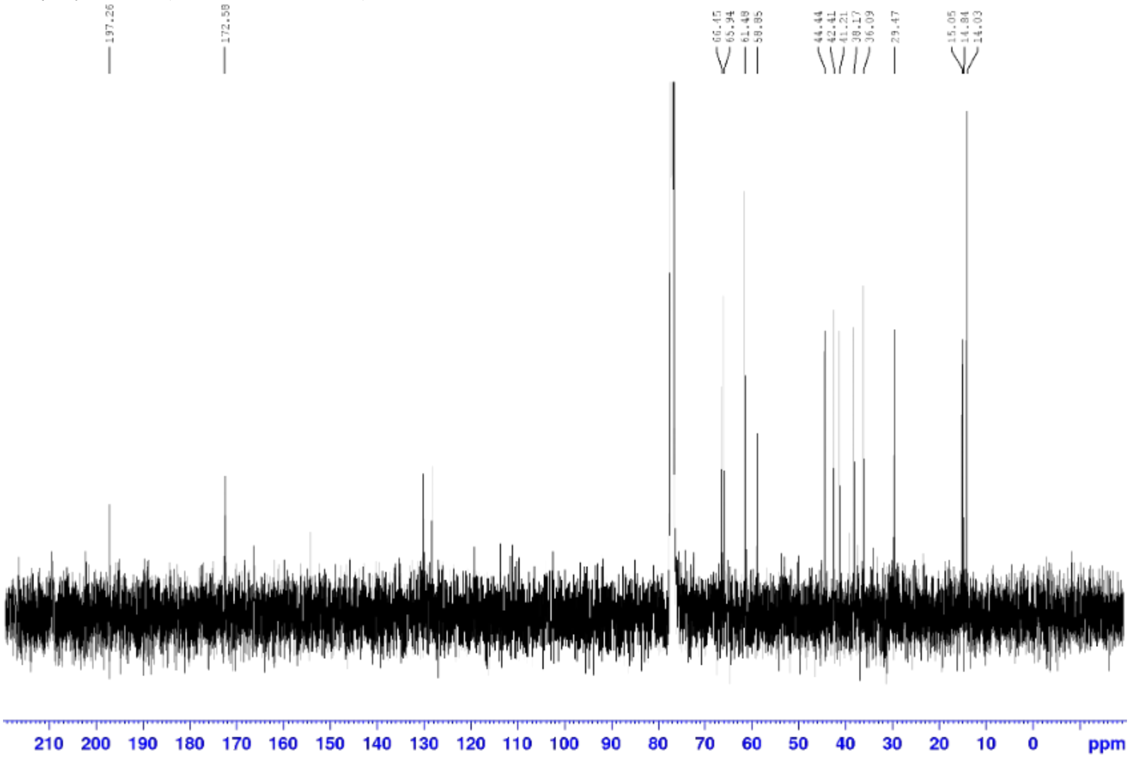

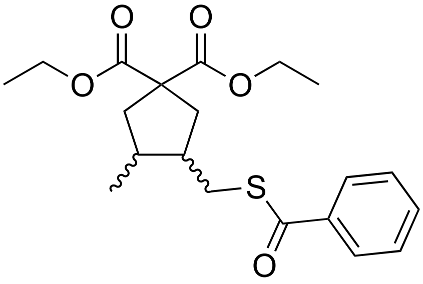

3c ^{1}H NMR (400 MHz, CDCl$_3$)

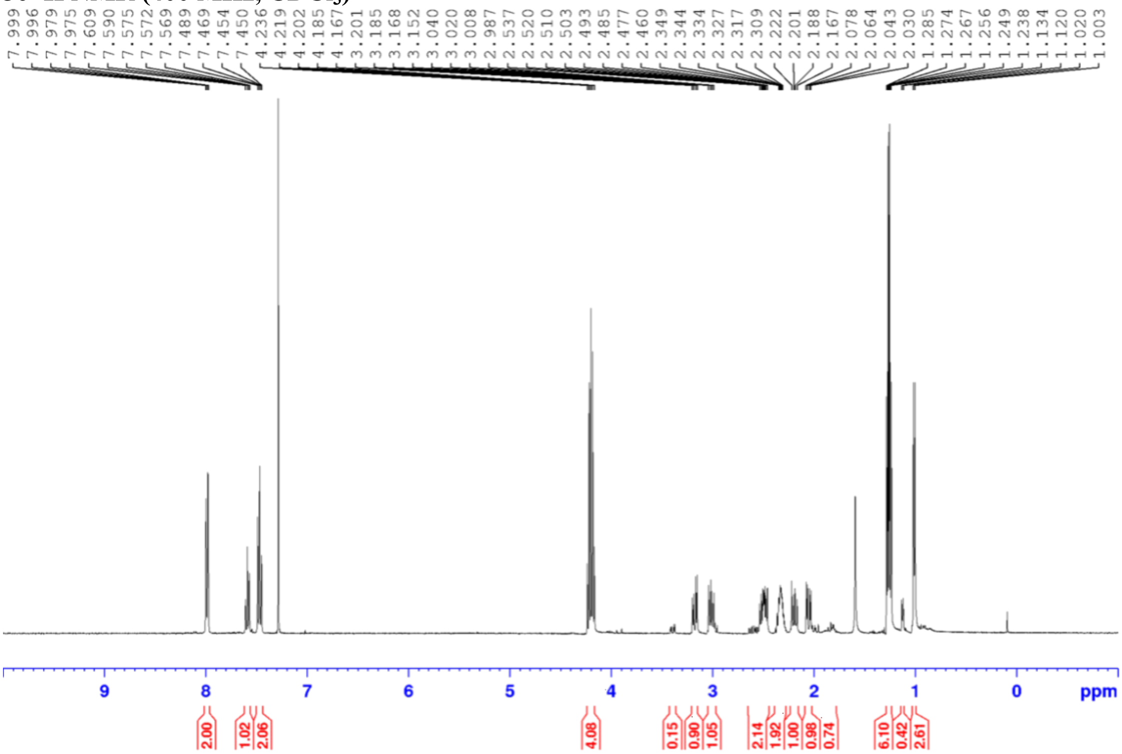

3c ^{13}C{^{1}H} NMR (151 MHz, CDCl$_3$)

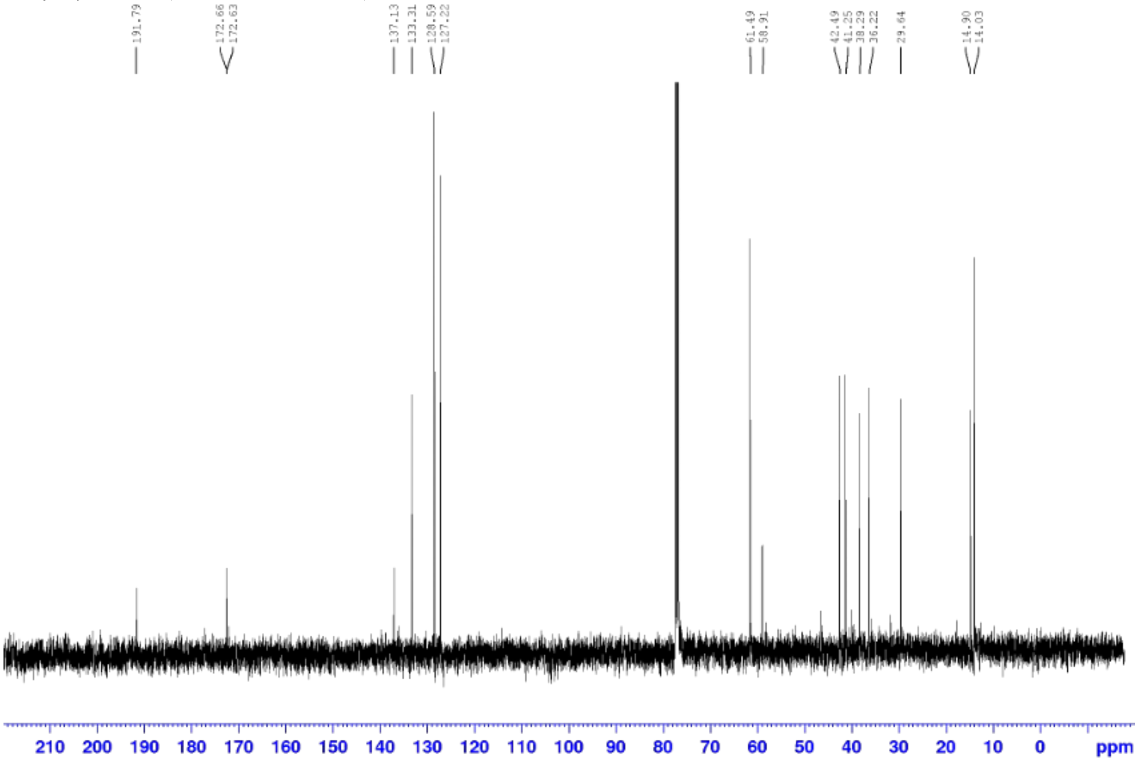

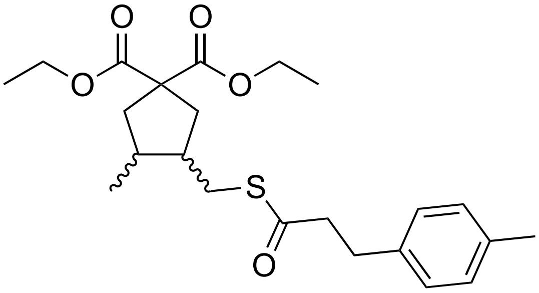

3d ^{1}H NMR (400 MHz, CDCl$_3$)

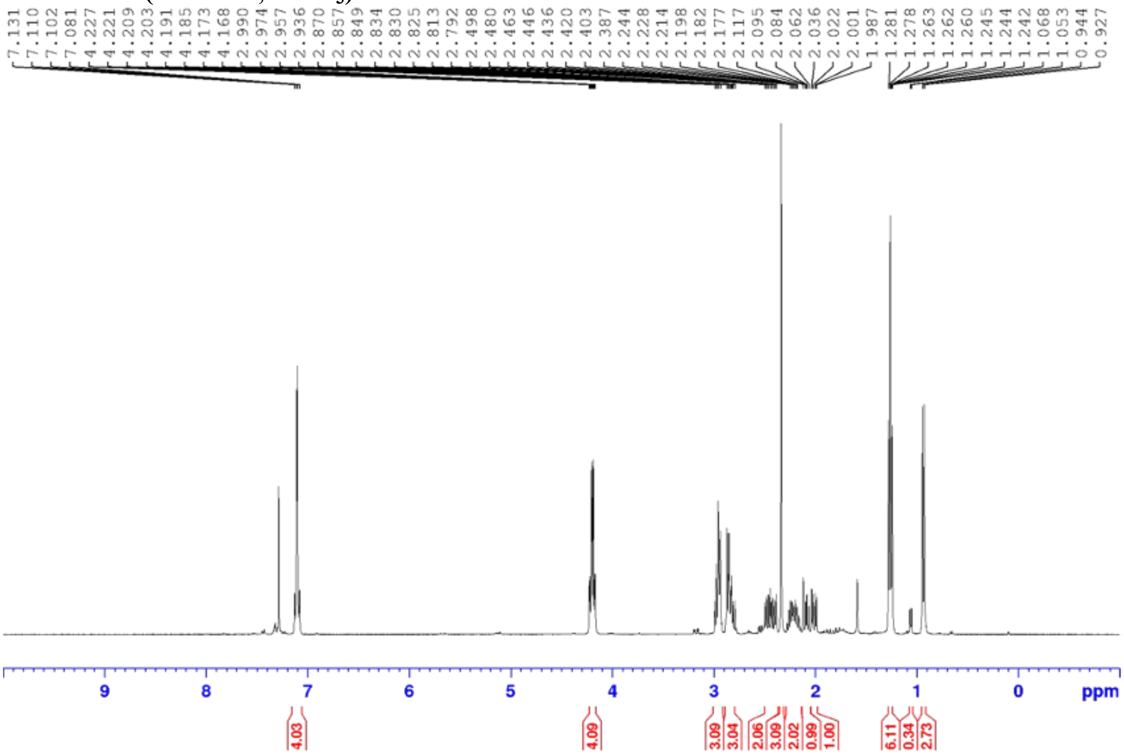

3d ^{13}C{^{1}H} NMR (151 MHz, CDCl$_3$)

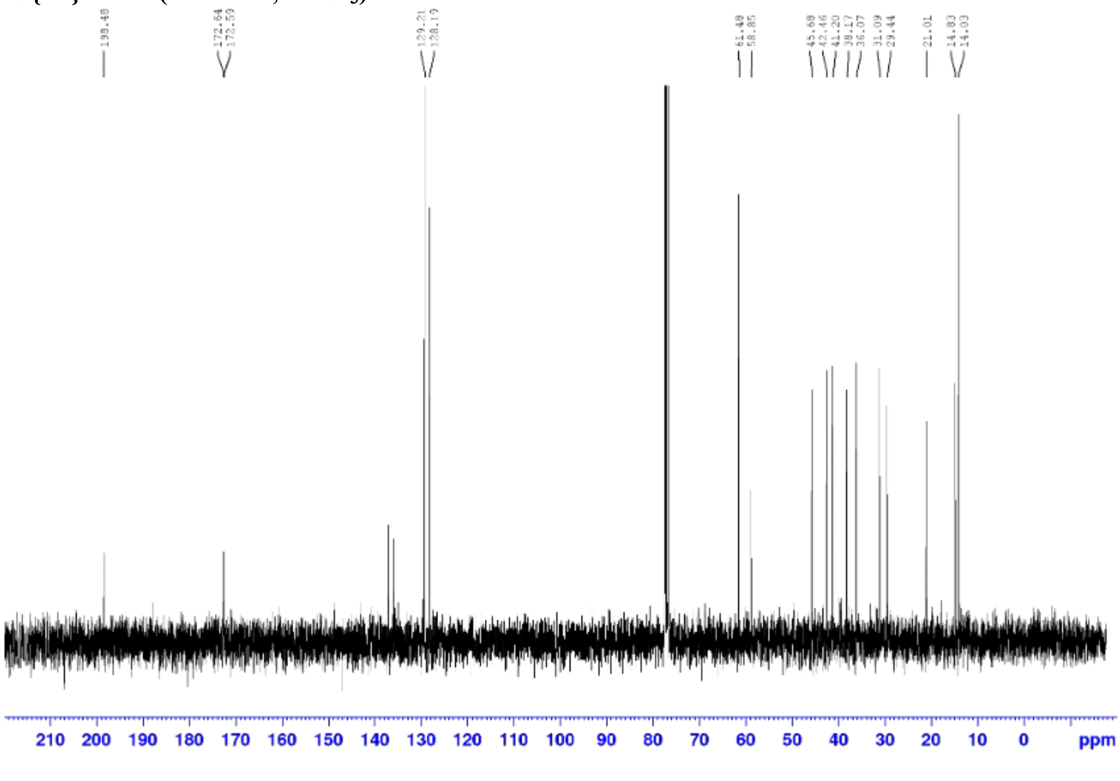

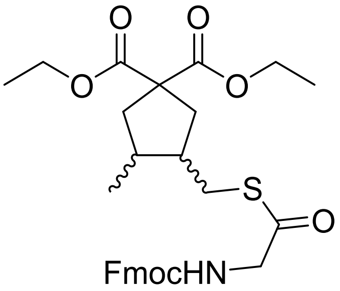

3e ^{1}H NMR (400 MHz, $CDCl_3$)

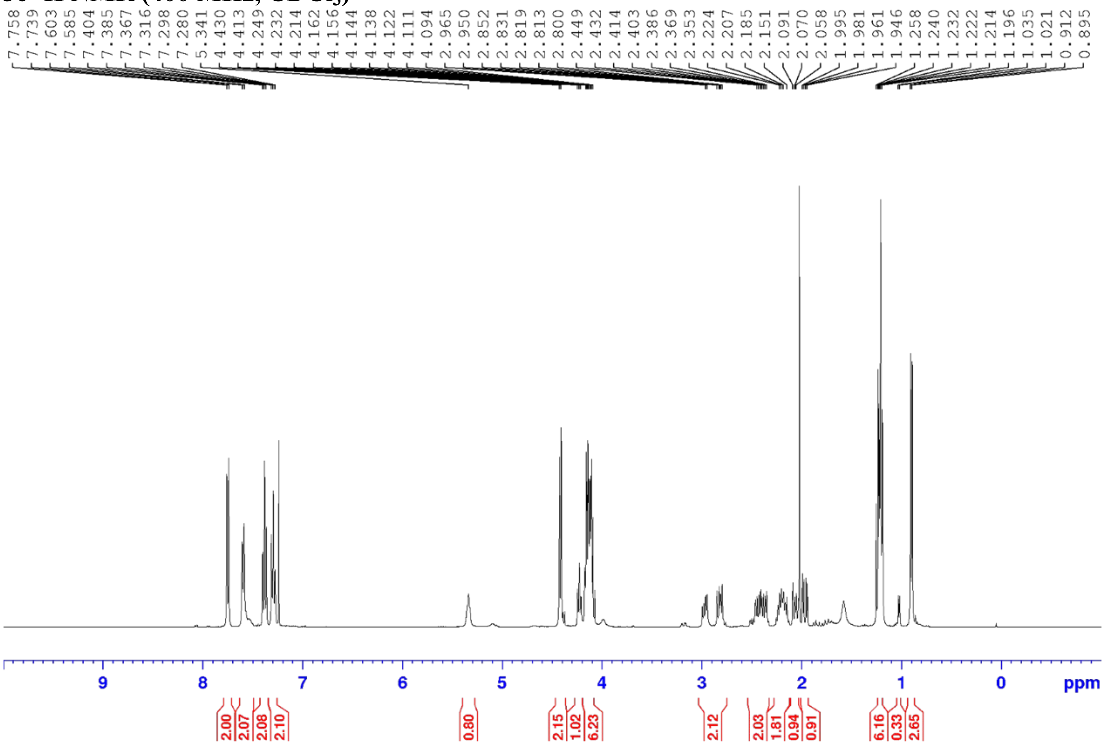

3e $^{13}C\{^{1}H\}$ NMR (151 MHz, $CDCl_3$)

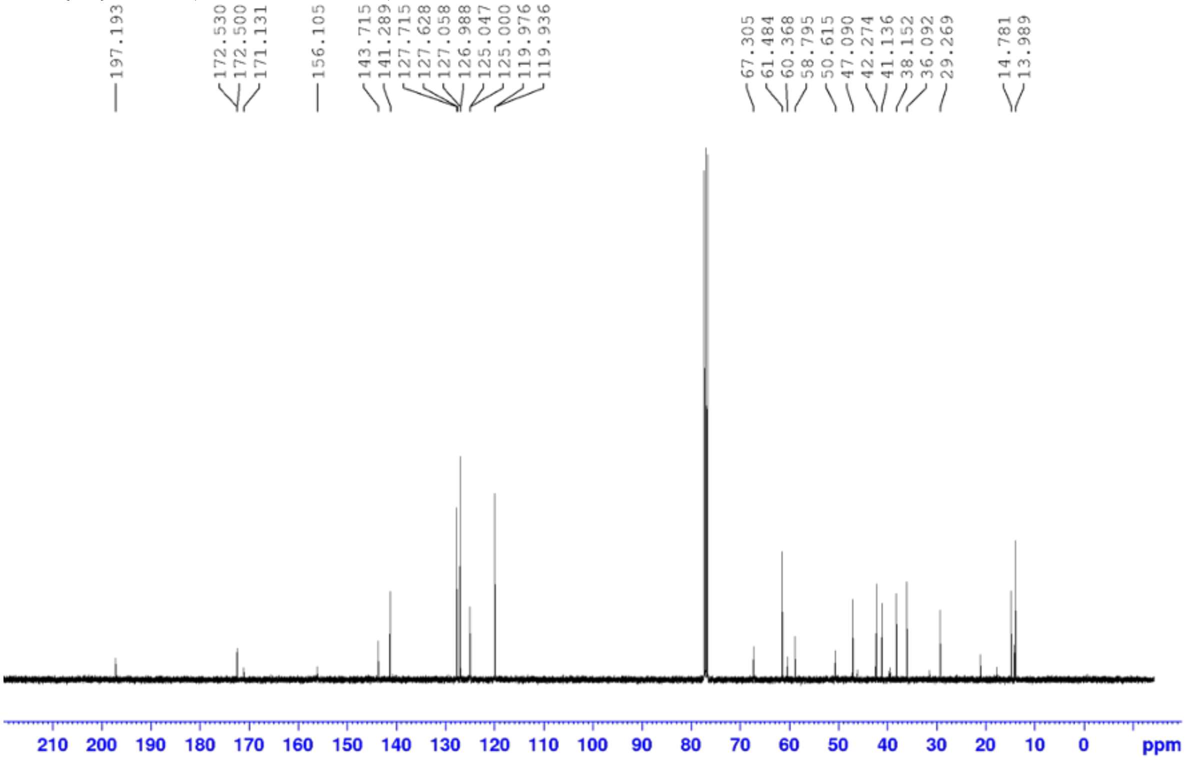

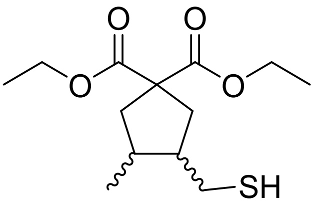

8 ¹H NMR (400 MHz, $CDCl_3$)

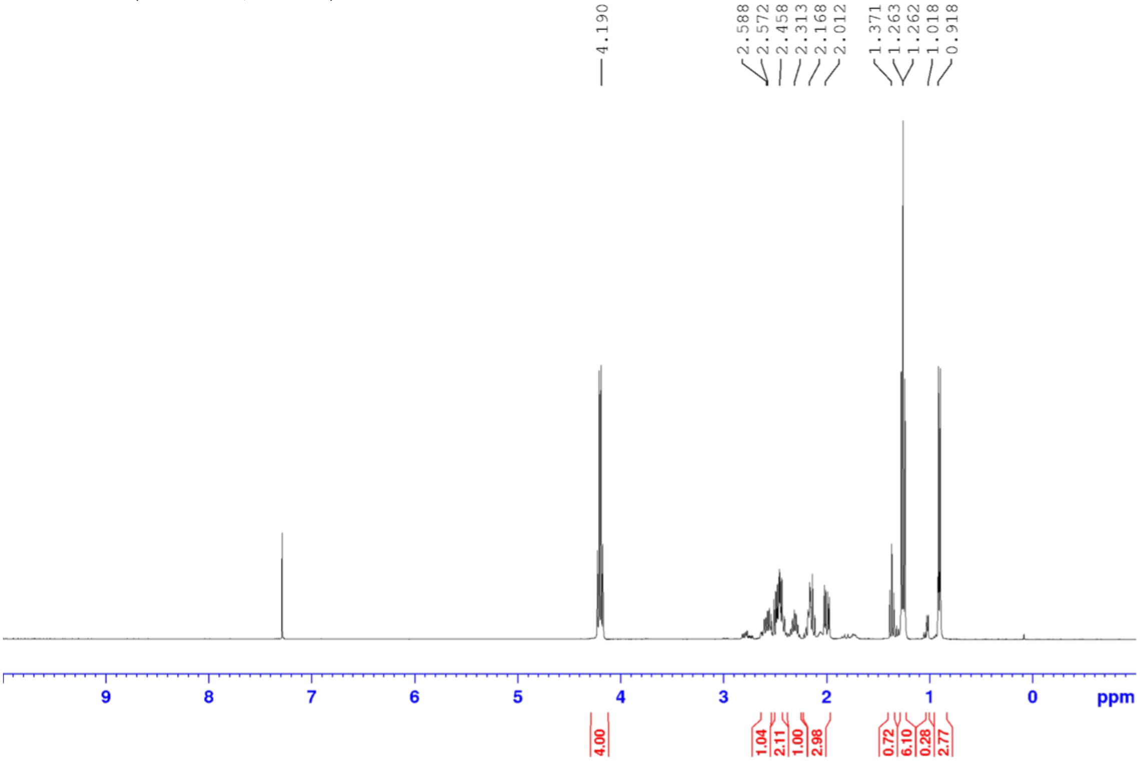

8 $^{13}C\{^1H\}$ NMR (151 MHz, $CDCl_3$)

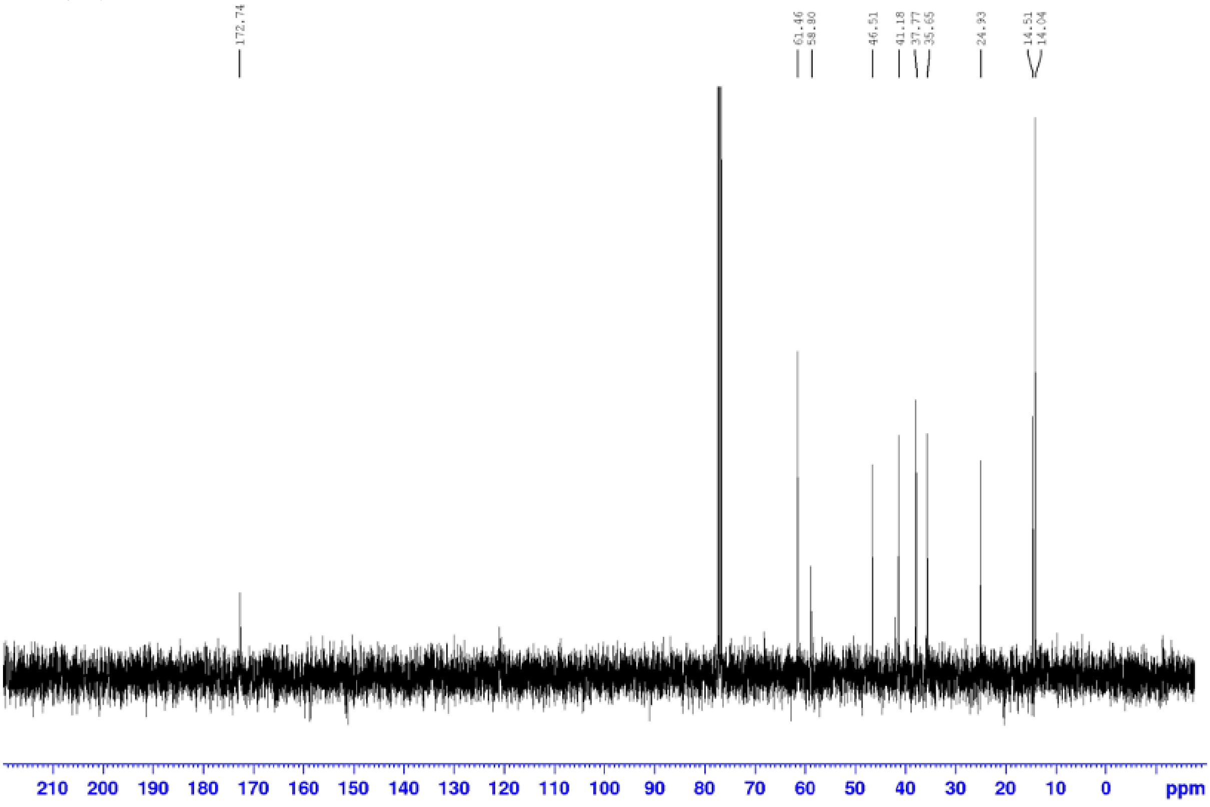

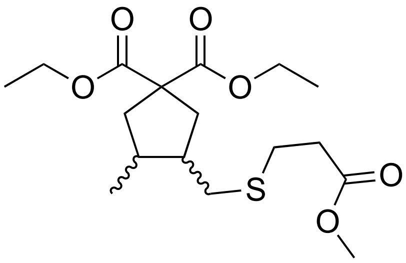

10a ^{1}H NMR (400 MHz, CDCl$_3$)

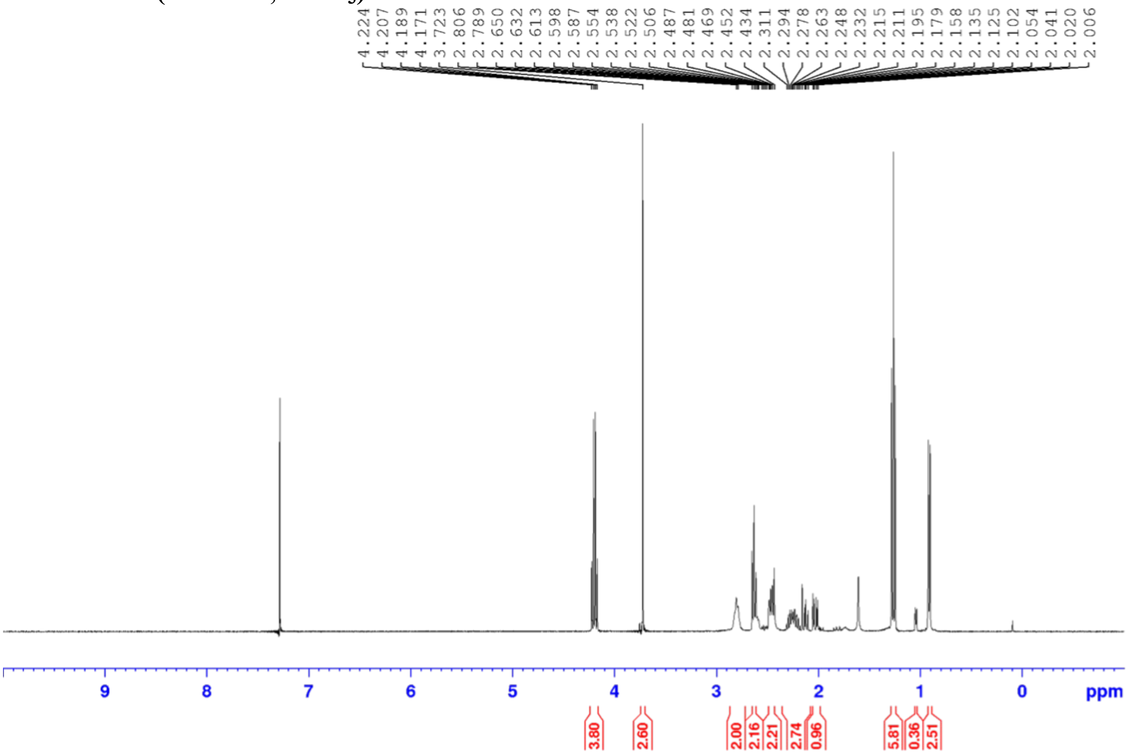

10a ^{13}C{^{1}H} NMR (151 MHz, CDCl$_3$)

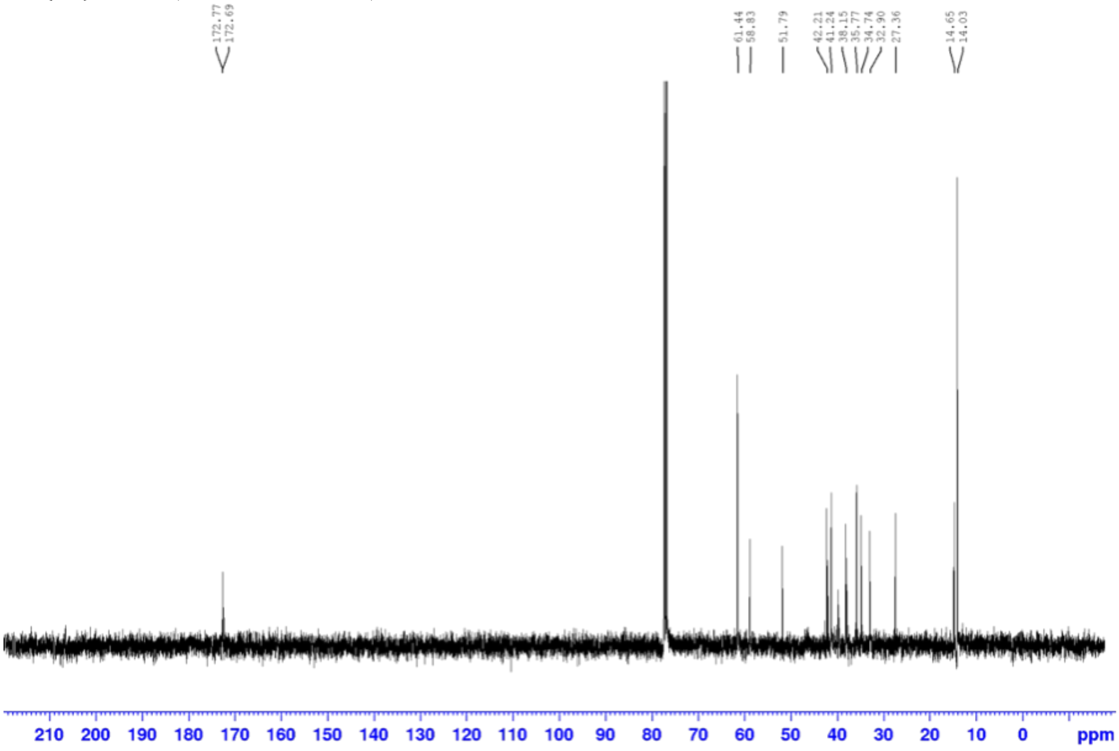

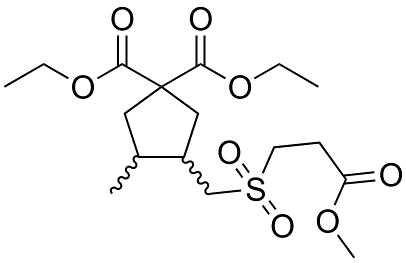

11 ¹H NMR (400 MHz, CDCl₃)

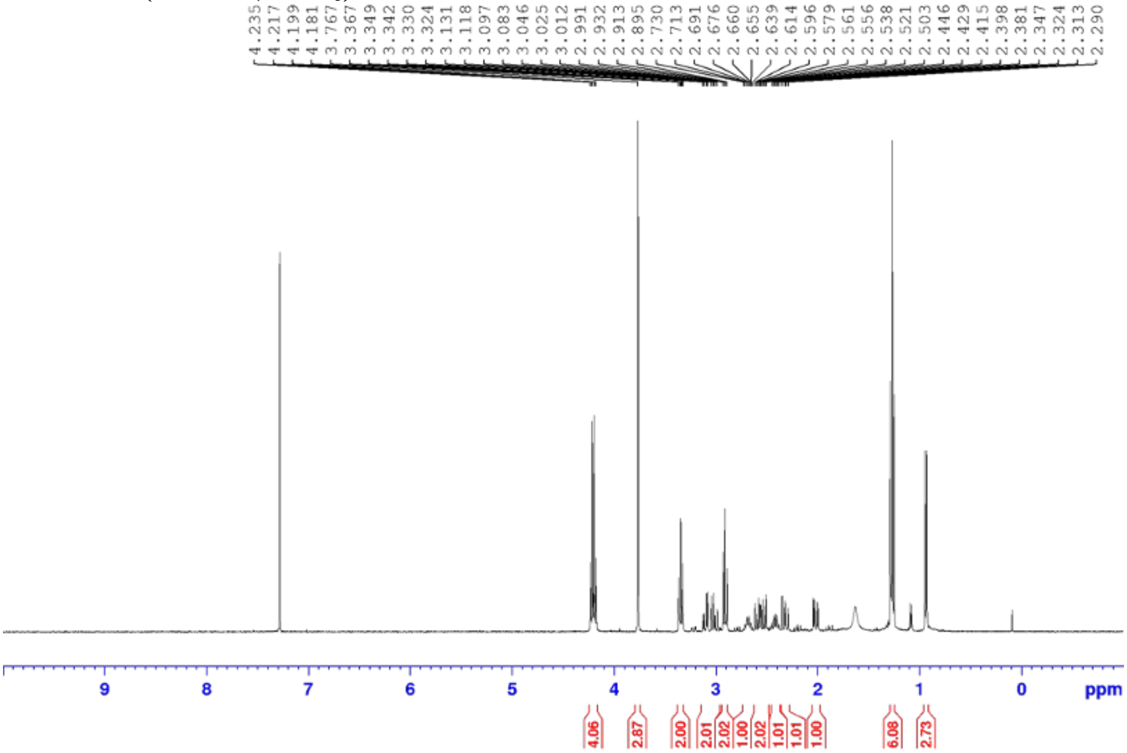

11 ¹³C{¹H} NMR (151 MHz, CDCl₃)

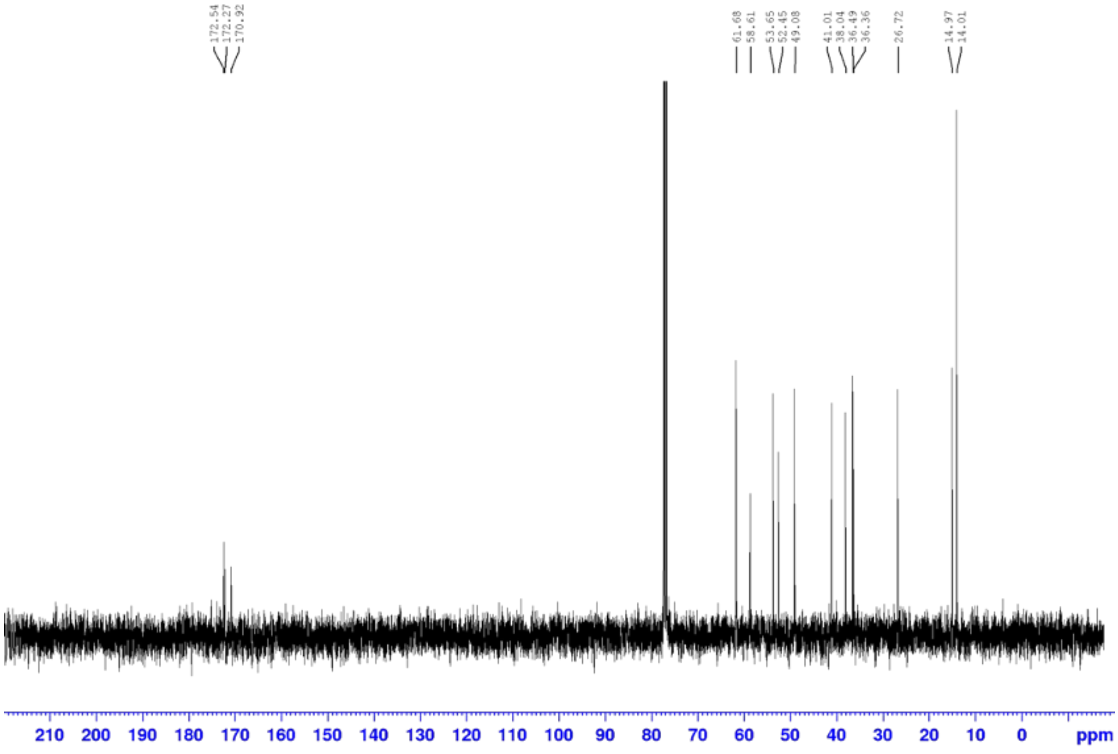

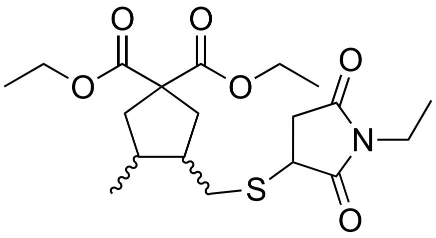

10b ^{1}H NMR (400 MHz, $CDCl_3$)

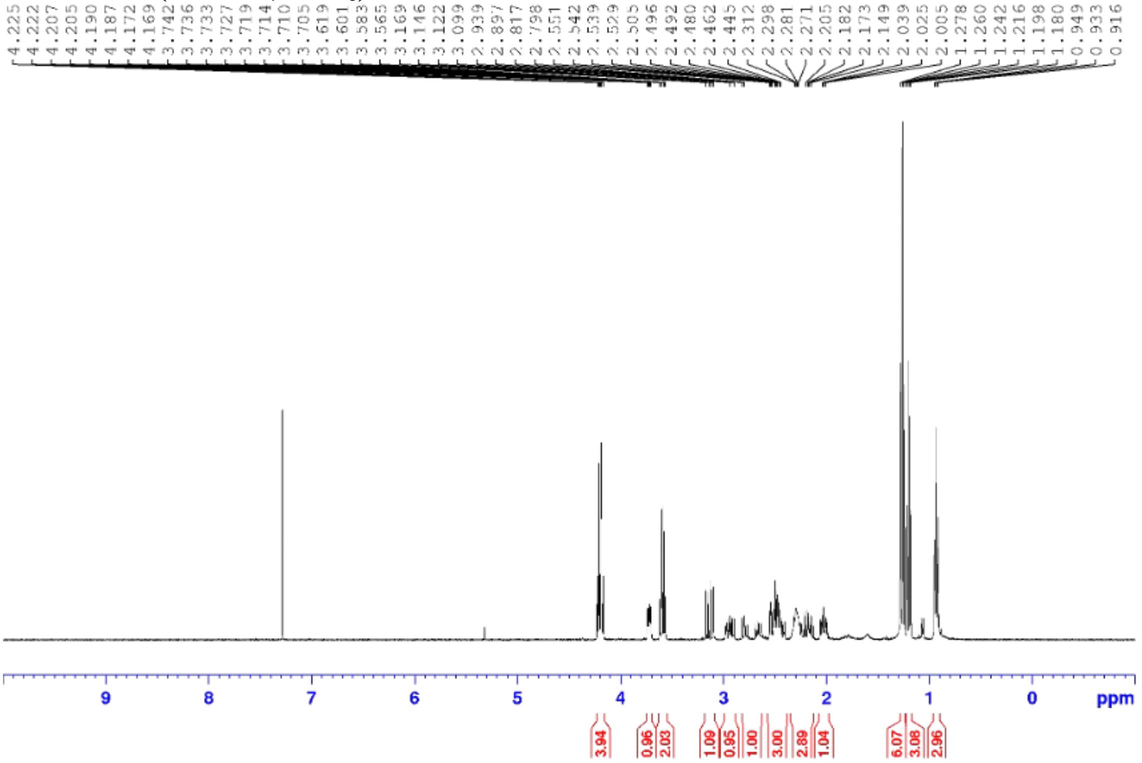

10b $^{13}C\{^{1}H\}$ NMR (151 MHz, $CDCl_3$)

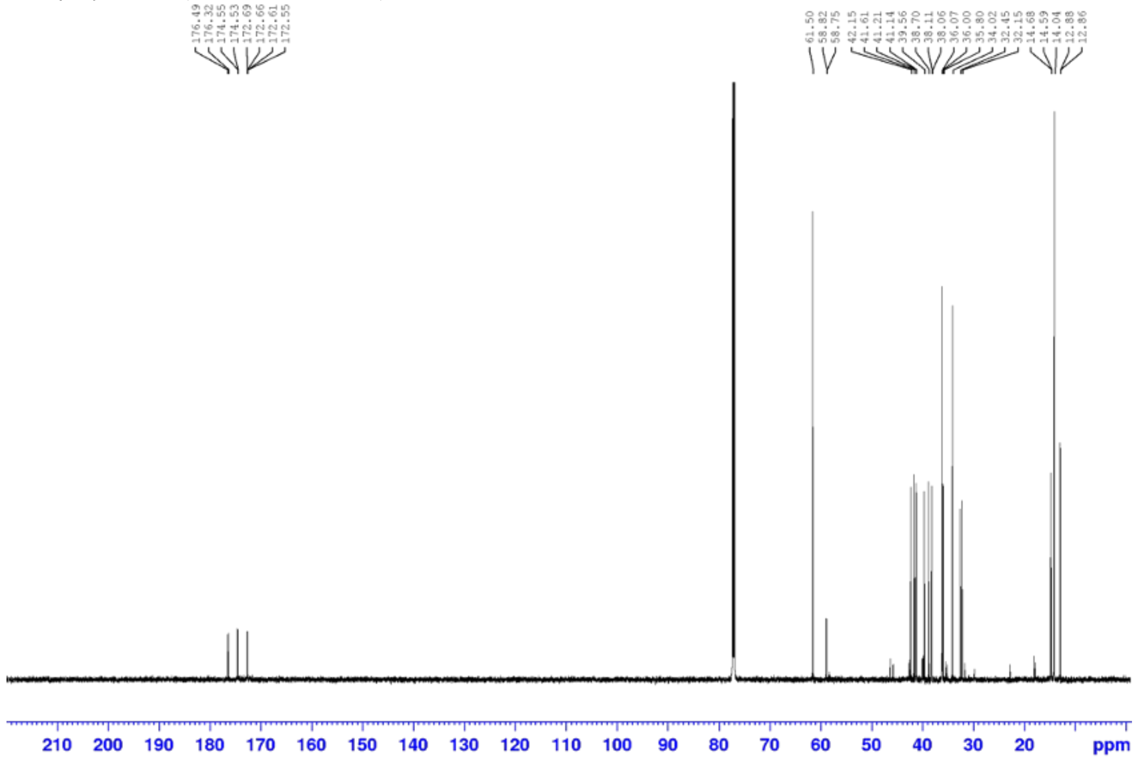

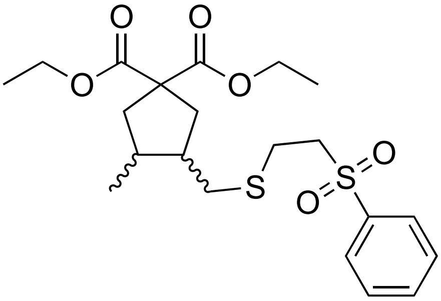

10c ^{1}H NMR (400 MHz, $CDCl_3$)

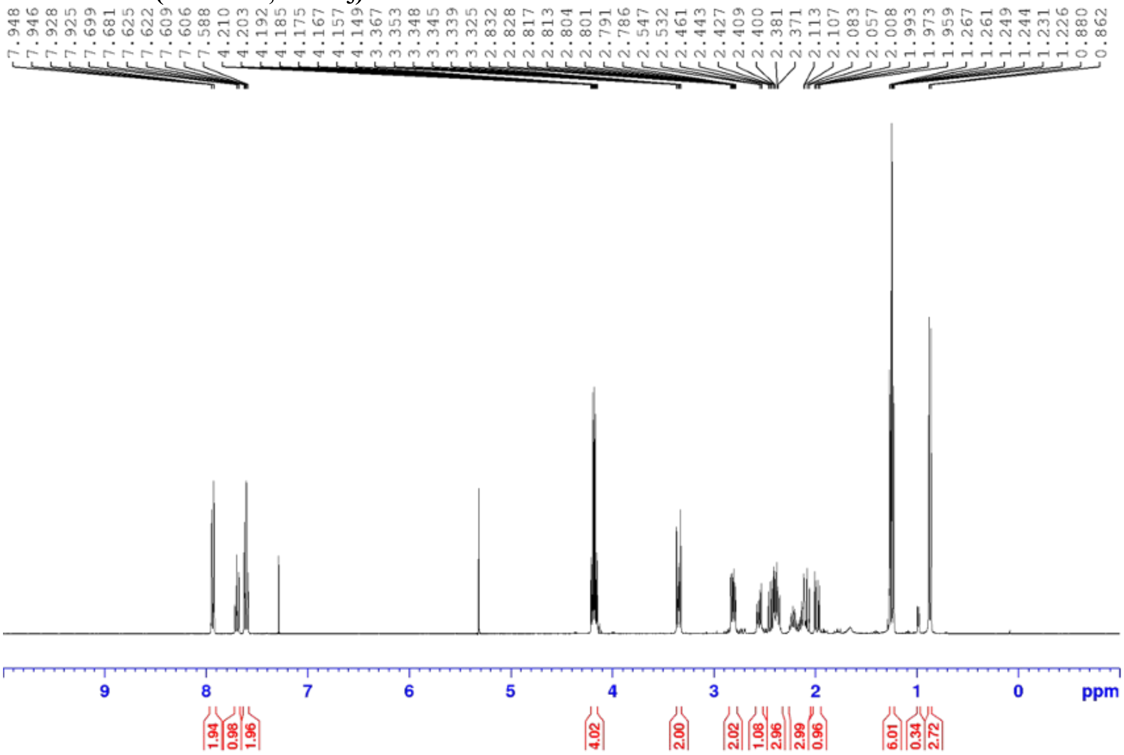

10c $^{13}C\{^{1}H\}$ NMR (151 MHz, $CDCl_3$)

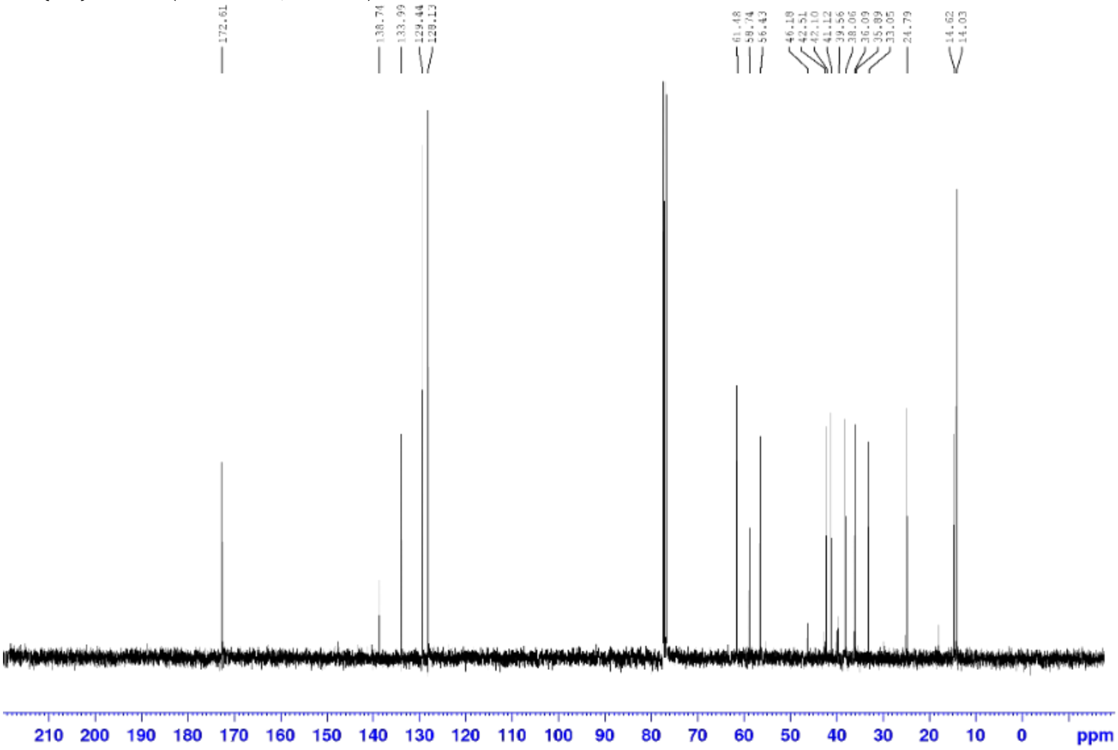

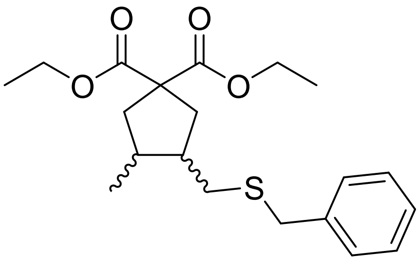

12 ^{1}H NMR (400 MHz, $CDCl_3$)

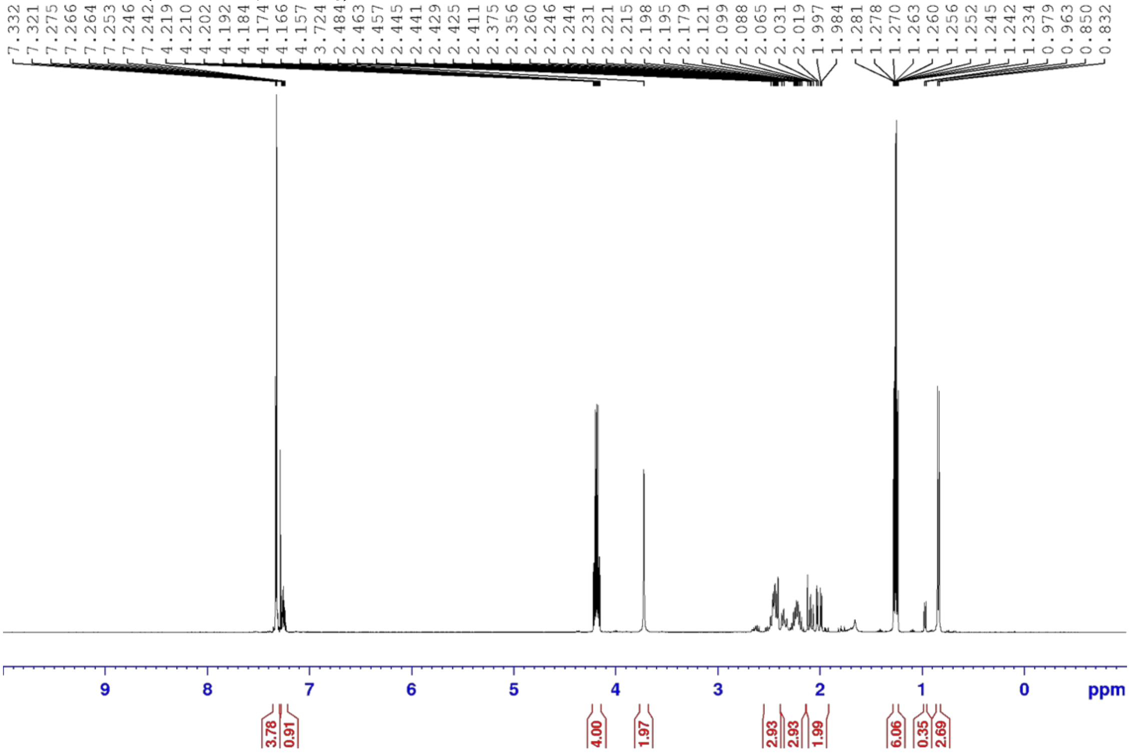

12 ^{13}C{^{1}H} NMR (151 MHz, $CDCl_3$)

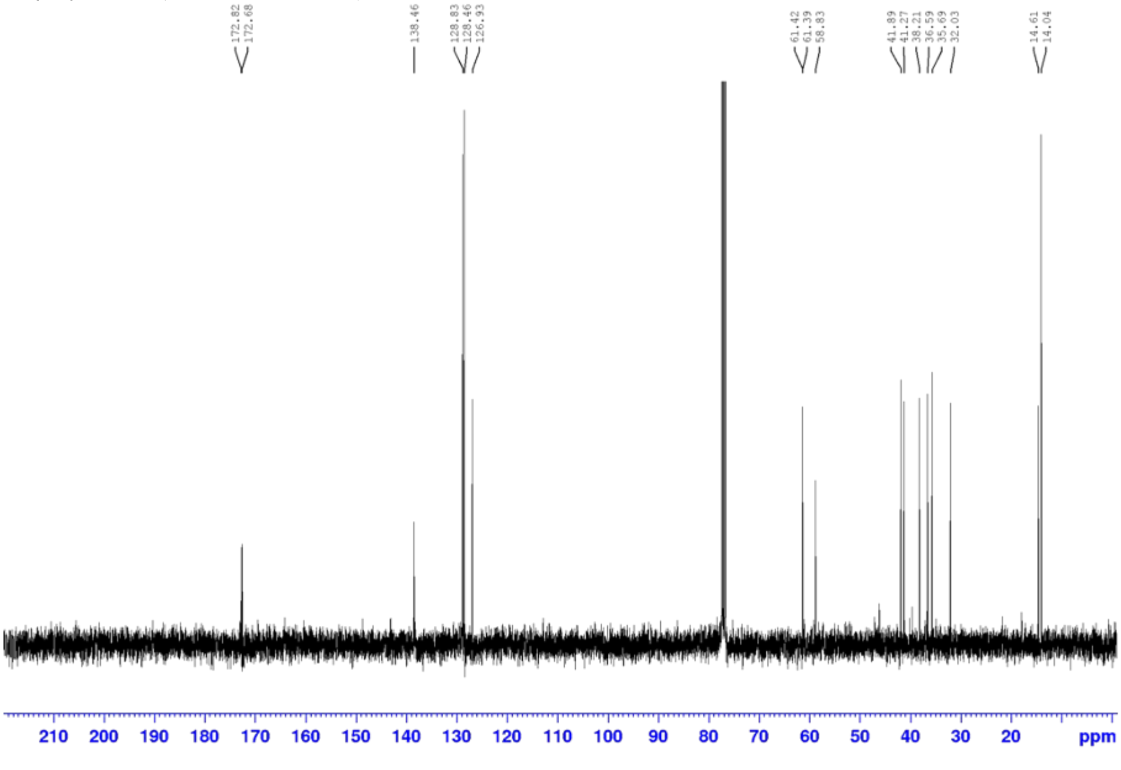

Supplement: Supplementary file 1 — jo3c00824_si_001.pdf [file jo3c00824_si_001.pdf]
